# Synthesis of model heterojunction interfaces reveals molecular-configuration-dependent photoinduced charge transfer

**DOI:** 10.1038/s41557-024-01578-x

**Published:** 2024-08-20

**Authors:** Jeroen Royakkers, Hanbo Yang, Alexander J. Gillett, Flurin Eisner, Pratyush Ghosh, Daniel G. Congrave, Mohammed Azzouzi, Zahra Andaji-Garmaroudi, Anastasia Leventis, Akshay Rao, Jarvist Moore Frost, Jenny Nelson, Hugo Bronstein

**Affiliations:** 1 Yusuf Hamied Department of Chemistry, University of Cambridge, Cambridge, UK; 2 Department of Physics, Imperial College London, London, UK; 3 Cavendish Laboratory, University of Cambridge, Cambridge, UK; 4 School of Materials Science and Engineering, Queen Mary University of London, London, UK; 5 Department of Chemistry, Imperial College London, London, UK

**Keywords:** Excited states, Conjugated polymers, Optical materials, Solar cells, Energy transfer

## Abstract

Control of the molecular configuration at the interface of an organic heterojunction is key to the development of efficient optoelectronic devices. Due to the difficulty in characterizing these buried and (probably) disordered heterointerfaces, the interfacial structure in most systems remains a mystery. Here we demonstrate a synthetic strategy to design and control model interfaces, enabling their detailed study in isolation from the bulk material. This is achieved by the synthesis of a polymer in which a non-fullerene acceptor moiety is covalently bonded to a donor polymer backbone using dual alkyl chain links, constraining the acceptor and donor units in a through space co-facial arrangement. The constrained geometry of the acceptor relative to the electron-rich and -poor moieties in the polymer backbone can be tuned to control the kinetics of charge separation and the energy of the resultant charge-transfer state giving insight into factors that govern charge generation at organic heterojunctions.

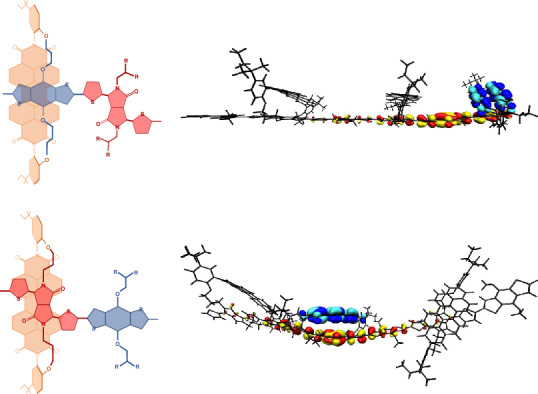

## Abstract (web summary)

The molecular interactions at an organic heterointerface govern the performance of many optoelectronic devices. Through a combination of synthesis, spectroscopy and modelling, it has now been shown that specific molecular interactions result in improved formation of the charge-transfer state.

## Main

The operation of organic semiconductor devices such as organic photovoltaics (OPVs) and organic photodetectors (OPDs) relies on photoinduced charge transfer (CT) at a heterojunction between electron-accepting and -donating components. The evolution of the excited state into separated charges via an intermediate CT state and the recombination of charge pairs back to ground are both dependent on the configuration of donor and acceptor units at the heterojunction, the energies of the states involved, and their associated electronic and vibrational interactions^1–3^. In OPVs, the best-performing conjugated polymer donors are structurally complex push–pull systems. Charge-transfer dynamics between such polymers and an electron acceptor are likely to depend, for example, on the position of the acceptor relative to the polymer’s electron-rich (push) or -poor (pull) moieties^4–10^. However these arrangements are difficult to control in solution- or vacuum-processed molecular heterojunctions, and difficult to probe experimentally, requiring specialized methods such as solid-state NMR^10–13^. Thus, despite its central importance, the relationship between CT dynamics and molecular configuration at organic heterojunctions has been difficult to establish.

Various methods have been applied to try to control and interrogate molecular orientation at donor–acceptor interfaces. In multiple component systems, such methods include the control of evaporated molecular bilayers through templating, the control of polymer face/edge-on orientations in solution-processed polymers (small-molecule blends through processing techniques; solvent, annealing, substrate) and control of polymer interchain contract by changing the side-chain positions^11,13,14^. Other attempts have used single macromolecular components where donor and acceptor units are chemically bound, including D–A dyad molecules or self-assembled amphiphilic molecules in structured interfaces^15–18^, polymers with pendant donor or acceptors^19–22^, and block–copolymers^23–25^. Despite the built-in linkages between donor and acceptor units, the range of possible donor–acceptor configurations in such systems is still large. Although computational modelling can be a powerful tool in describing molecular excited states and interactions, the large conformational space for these intrinsically disordered (and generally poorly structurally validated) systems makes it difficult to validate theories or optimize the design of interface structures. So far there are few strategies to construct model interfaces for donor–acceptor bulk heterojunctions in isolation allowing the effect of molecular parameters can be studied with precision.

Here we demonstrate a synthetic strategy to design and structurally control through-space model interfaces with a constrained geometry. We synthesize conjugated polymer donors that are covalently linked to an electron-accepting unit at a predetermined distance, position and orientation, as models for a bulk–heterojunction interface. The conformational phase space for the D–A interface is small enough that the properties of the interfacial states can be modelled. This also allows the CT dynamics and state energies to be studied as a function of the location of electron-rich and -poor units. We stress that the aim of this investigation is to study the processes at the interface that control the initial charge-separation process, rather than to achieve high power conversion efficiency in organic photovoltaic devices. Efficient photocurrent generation would require the through-space structures to be connected with transport pathways to extract the generated charge carriers away from the donor–acceptor interfaces and reduce recombination at the interface. These chemical requirements are beyond the scope of the current study.

With this synthetic approach, the key interfacial parameters that have been suggested to affect OPV performance (donor–acceptor separation, orientation, rigidity, position) can be studied systematically, in different formats (in solution, in dense films and dispersed in film), and in isolation from other intermolecular interactions. To demonstrate the power of this approach, we report a series of polymers in which the exact position of the acceptor relative to either the electron-rich or -poor section of a conjugated polymer is controlled, and the properties of the resulting interfacial CT states can be interrogated. We synthesize the desired macrocyclic through-space donor–acceptor monomer, and then co-polymerize. We target benzodithiophene- (BDT) and diketopyrrolopyrrole- (DPP) based conjugated polymers because they are the most common and successful family of materials in OPV^26^. We chose a perylene diimide (PDI) derivative as the acceptor because it is a commonly used chemical motif in acceptor materials, and is symmetric^27,28^.

## Results

**Fig. 1:**
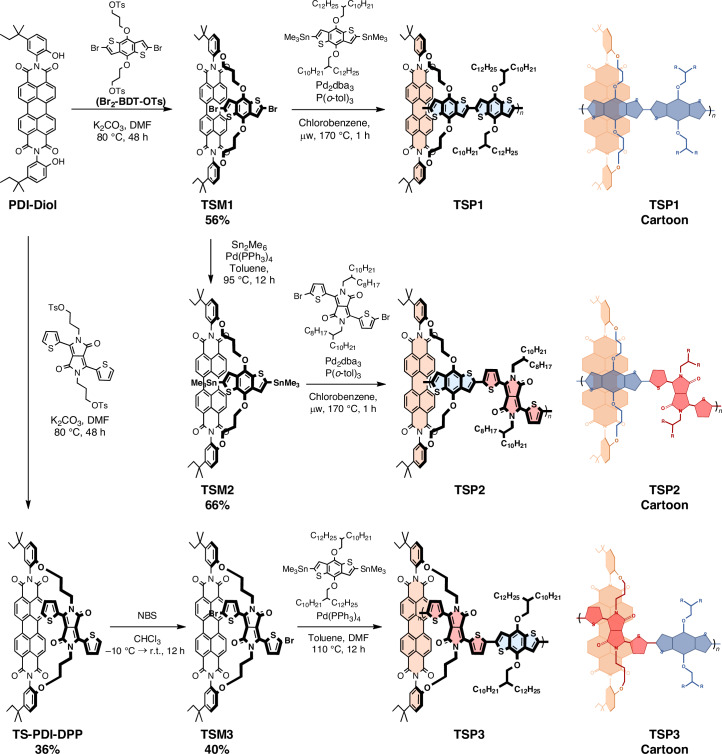
Synthetic routes to model-interface polymers TSP1, TSP2 and TSP3. Here we show the key synthetic macrocycle formation and polymerization steps to afford the through-space polymers. Top-down representations are also shown for clarity. dba, dibenzylideneacetone; Ts, tosyl; NBS, *N*-bromosuccinimide; DMF, dimethylformamide; tol, toluene; BDT, benzodithiopehene. Source data

The three synthesized through-space polymers—TSP1, TSP2 and TSP3—are shown in Fig. 1. TSP1 is a through-space polymer comprising a PDI unit connected to a BDT–BDT unit, whereas TSP2 and TSP3 have the PDI unit connected to a BDT–DPP unit. The PDI is attached face-on to the BDT unit for TSP2 and to the DPP for TSP3. The molecular weights of the polymers are as follows: TSP1 (*M*_*n*_ = 19.7 kDa, polydispersity index = 2.3), TSP2 (*M*_*n*_ = 11.6 kDa, polydispersity index = 1.7) and TSP3 (*M*_*n*_ = 72.5 kDa, polydispersity index = 2.3), with the slightly lower molecular weight of TSP2 reflecting the difficulty in purifying the TSM2 monomer. Furthermore, the reference polymers Ref-P1, a BDT–BDT polymer with no tethered PDI (*M*_*n*_ = 63.1 kDa, polydispersity index = 3.68), and Ref-P2, a BDT–DPP copolymer with no tethered PDI (*M*_*n*_ = 58.2 kDa, polydispersity index = 3.78), were made.

### Experimental characterization of excited states

**Fig. 2:**
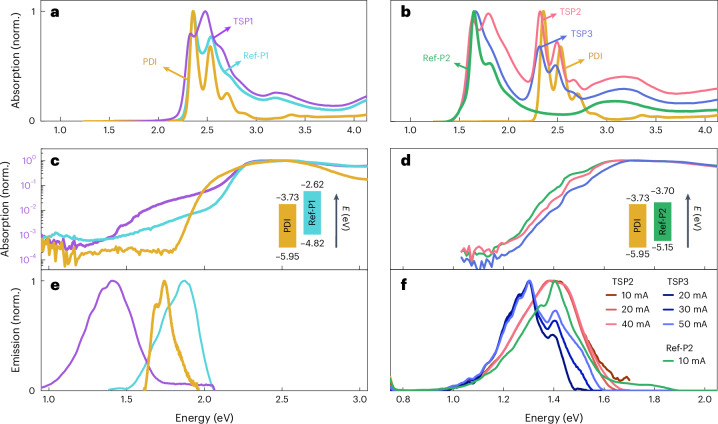
Optoelectronic characterization. **a**,**b**, Absorption spectra in dilute chloroform solution of Ref-P1, PDI and TSP1 (**a**), as well as Ref-P2, PDI, TSP2 and TSP3 (**b**). **c**,**d**, Photothermal deflection spectroscopy spectra of Ref-P1 (cyan), PDI (gold) and TSP1 (purple) (**c**), as well as Ref-P2 (green), TSP2 (pink) and TSP3 (blue) thin films (**d**). **e**,**f**, Electroluminescence spectra of Ref-P1 (cyan), PDI (gold) and TSP1 (purple) (**e**) at an injection current of 100 mA cm^–2^, as well as TSP2, TSP3 and Ref-P2 (**f**) as a function of the injection current. The insets show energy level diagrams of PDI and Ref-P1 (**c**), and PDI and Ref-P2 (**d**). The HOMOs are obtained from ionization potentials of solid films using air photoemission spectroscopy, whereas the LUMOs are from those HOMO energies plus the corresponding optical bandgaps. Source data

The absorption spectra of all three through-space polymers—along with the two reference polymers and a relevant PDI—in chloroform solution are shown in Fig. 2a,b. The maximum absorption peak of TSP1 (Fig. 2a) is slightly blue-shifted compared with Ref-P1, with a ${\lambda }_{\max }$ at 495 nm (2.5 eV), whereas the absorption onset is red-shifted by 0.1 eV. The absorption spectrum shows clear features of both the PDI and Ref-P1 polymer units, and strong photoluminescence quenching was observed compared with Ref-P1 (Supplementary Fig. 1 and Supplementary Table 1). For TSP2 and TSP3 (Fig. 2b), the spectrum can be divided into the 450–570 nm (2.2–2.7 eV) region, where the PDI chromophore absorbs, and the 570–800 nm (1.5–2.2 eV) region, which corresponds with absorption from the conjugated polymer backbone. Both Ref-P2 and TSP3 have absorption maxima at ~760 nm (~1.63 eV) with a vibronic shoulder at ~690 nm (~1.80 eV). The absorption spectrum of TSP2 shows an inversion of the 0–0 and 0–1 intensities, such that the maximum is found at ~690 nm with a smaller peak at ~760 nm.

We performed sensitive photothermal deflection spectroscopy (PDS) (Fig. 2c) and electroluminescence (Fig. 2e) on TSP1 films to experimentally detect the presence of CT states. In PDS, a clear absorption signal (Fig. 2c, purple line) below the onset of the strong polymer absorption (cyan line) is observed, at an energy (1.4–1.8 eV) that is lower than the optical gap of either pure component and consistent with the difference between the measured HOMO and LUMO energies of the polymer and PDI, respectively (Fig. 2c inset diagrams). Furthermore, the electroluminescence spectrum of TSP1 shows emission from a state at a lower photon energy (Fig. 2e, purple) than that of any locally excited states in PDI or Ref-P1 (Fig. 2a). The narrow band-gap Ref-P2, TSP2 and TSP3 polymers do not exhibit any substantial differences when using PDS (Fig. 2d), possibly due to the fact that the formed CT states lie closer in energy to the locally excited states. However, injection-dependent electroluminescence measurements (Fig. 2f) show the presence of distinguishable low-energy states in TSP3 but not TSP2, demonstrating that the regioisomeric manipulation of the through-space donor–acceptor interaction has been successful.

### Modelling and analysis of excited states

We first calculate and analyse the excited-state structures of these materials to aid in interpreting their response to photoexcitation. This is uniquely possible in this model system due to the constrained molecular conformation, allowing us to model a reduced parameter space compared with conventional (non-bonded) donor–acceptor systems, where the large number of possible donor–acceptor configurations^29^ makes modelling of excited states at interfaces extremely challenging. To represent the interface at room temperature, we sample a thermodynamic ensemble of conformers using a high-throughput tight-binding molecular dynamics method—which will include the stiffening effect of conjugation—rather than use an athermal (frozen) model^30^. Statistical analysis of the geometries of the PDI acceptor unit relative to the polymer backbone from these ensembles shows that the PDI is oriented almost co-facially (±20°) to the polymer backbone and is constrained to sit within a tight seperation range (3–3.5 Å away; Supplementary Fig. 7). Excited-state calculations using time-dependent density functional theory (TDDFT)^31–33^ were performed on samples from the thermal ensemble after a few steps of DFT optimization (to get consistent bond lengths) using the B3LYP/6-31G* functional and basis set. The ensemble was used to efficiently find the global minimum by further DFT geometry optimization of five sampled conformers from the ensemble, with the most energetically stable conformation then selected for detailed calculations and characterization of the excited state.

**Fig. 3:**
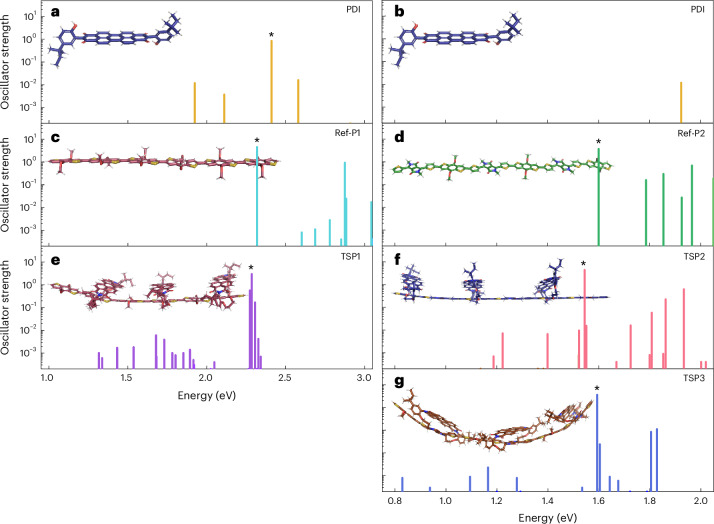
Modelling of excited states. **a**–**g**, Calculated excited-state spectra of PDI (**a**,**b**) shown twice on different energy scales to aid in vertical comparison, and trimers of Ref-P1 (**c**), Ref-P2 (**d**), TSP1 (**e**), TSP2 (**f**) and TSP3 (**g**). The asterisks indicate the lowest excited local excitonic state in each molecule.

Beginning with an analysis of TSP1, the absorption spectra of the first 28 excited singlet states of the optimized PDI, Ref-P1 and TSP1 structures are shown in Fig. 3a–c. In TSP1, additional states with CT character and low oscillator strengths are present at lower energies than the lowest states in either Ref-P1 or PDI. Together with the experimental results, this confirms the presence of an interfacial state of CT character lying below the local exciton of either the PDI or BDT polymer, with good agreement between the experimental and modelling results. This demonstrates the capability of our synthetic approach to build a donor–acceptor heterojunction.

We now turn to TSP2 and TSP3 to see whether changing the position of the polymer co-monomer (BDT or DPP) relative to the acceptor affects the excited-state properties of the interface. First, we note that Ref-P2, TSP2 and TSP3 (Fig. 3d,f,g) all have lower-energy locally excited states compared with TSP1 (Fig. 3e) and Ref-P1 (Fig. 3c). This is due to the push–pull nature of Ref-P2, which leads to a lowered LUMO energy that in turn reduces the energy offset between the polymer donor and PDI acceptor in TSP2 and TSP3 compared with TSP1. We believe that the close energetic proximity of the weakly absorbing CT states to the strongly absorbing excitonic states made them indistinguishable in the PDS spectra (Fig. 2d). The experimentally measured electroluminescence (Fig. 2f) is in agreement with the lower-energy CT states seen in TSP3 compared with those seen in TSP2 (Fig. 3f,g and Supplementary Fig. 9). Furthermore, we note that the oscillator strengths of CT states calculated for TSP2 are generally greater than those of TSP3, which is in agreement with the experimentally measured electroluminescence spectra (Supplementary Fig 10), in which emission from TSP2 is more than an order of magnitude brighter than TSP3 at the same injection current with the same device architecture. The brighter CT emission in TSP2 may result from intensity borrowing enabled by the small energy gap between the CT state and closest-lying excitonic state^34^.

We acknowledge here that as the experimental validation of CT states (EL and PDS) was performed on thin-film samples, they therefore may include contributions from intermolecular CT states. However, given the strong agreement with the modelled intramolecular CT states—as well as the strong intramolecular coupling due the face-on orientation between the PDI and polymer moiety—we believe that the CT state emission and absorption are probably dominated by intramolecular interactions, even in thin films.

The above findings demonstrate that the constrained relative conformation of the donor and acceptor units in the synthesized molecules allows for accurate modelling of excited states of both singlet and CT character, with theoretical state distributions reproduced well by experimental characterization. The results show that the different molecular structures of the three TS polymers have a substantial impact on the interfacial CT state energies and brightnesses. We now continue our analysis focusing on the effect of donor moieties (BDT and DPP) on the acceptor (PDI).

### Electron–hole distributions

To understand how the differences in the arrangement of molecular components relate to the observed state energy and brightness, we analysed our TDDFT calculations using the TheoDORE package^35^ to extract the CT character and charge distributions of the states. Fragment-based analysis allows us to study the electron and hole distributions of the excited states on individual moieties of the through-space polymers.

**Fig. 4:**
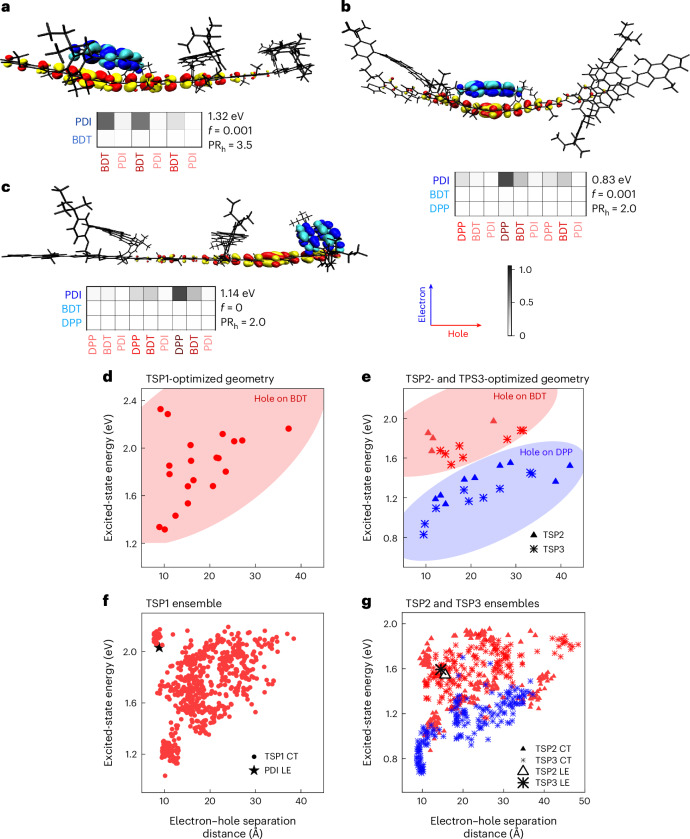
Modelling excited-state charge distribution. **a**–**c**, Charge distribution of lowest calculated excited state for trimers of TSP1 (**a**), TSP2 (**b**) and TSP3 (**c**). Their oscillator strengths (*f*) and participation ratios (PR), which indicate the delocalization of the hole, are shown. **d**,**e**, The excited-state energy versus the electron–hole separation distance of calculated states of optimized trimers TSP1 (**d**), and TSP2 and TSP3 (**e**), are shown in the middle row. Blue symbols indicate the hole is on DPP, whereas red symbols indicate the hole is on BDT. The electron is always on the PDI. **f**,**g**, The excited-state energy versus electron–hole separation distance of all calculated states of the thermodynamic ensemble of conformers for trimers of TSP1 (**f**), and TSP2 and TSP3 (**g**). Blue symbols indicate CT states where the hole is on DPP, whereas red symbols indicate CT states where the hole is on BDT. The black symbols represent the averages of the calculated locally excited excitonic states.

The calculated electron–hole distributions of the lowest excited state for all three TS polymers (trimers) are visualized through the natural transition orbital decomposition, as well as electron–hole correlation plots indicating the location of the hole (*x*-axis) and electron (*y*-axis) on the polymer backbone and PDI (Fig. 4a–c). Correlation plots and natural transition orbital decompositions for higher-energy states are shown in Supplementary Figs. 12–14. In all TS polymers, the lowest excited state showed CT character, with the electron strongly localized on a single PDI moiety. For TSP1, the hole is delocalized (participation ratio, ${\rm{P{R}}}_{\rm{h}}=3.5$) along the BDT–BDT backbone while the electron lies on the adjacent PDI, suggesting strong interactions between the active PDI and BDT moieties. For both TSP2 and TSP3, the hole is more localized (${\rm{P{R}}}_{\rm{h}}\approx \,2$) on the DPP unit, leading to a larger average separation between electron and hole in TSP2 than in TSP3.

To determine whether this picture is consistent, we plot the energy of all excited states in TSP1, TSP2 and TSP3—all modelled as trimers—as a function of the electron–hole separation (Fig. 4d,e)^36^. For TSP1 (Fig. 4d), the CT states generally increase in energy with increasing electron–hole separation, which can be explained by the reduction of the Coulombic interaction. We can distinguish two families of CT states in TSP2 and TSP3 (Fig. 4e): states with a stronger localization of the hole on the DPP moieties (blue symbols), and states with the hole more localized on the BDT moieties (red symbols). At the same average electron–hole separation distance, the states where the hole is localized on the DPP are lower in energy than those where it is localized on the BDT fragments, and this is true for either relative position of the PDI. The CT states in TSP3 therefore have generally lower energies than TSP2, as the electron–hole separation is generally shorter due to the proximity of the DPP moiety to PDI in TSP3. The observations made above for the optimized structures are also visible in the collective CT states generated by the full ensemble of conformers (Fig. 4f,g). In the TSP1 ensemble, the excited-state energies are spread across a wider range at the same electron–hole separation. This is consistent with the large delocalization of holes in Ref-P1 and the configuration space explored by the ensemble. Furthermore, TSP2 showed a narrower spread of CT energies than TSP3, despite having similar delocalization characteristics.

### Kinetics of excited states

**Fig. 5:**
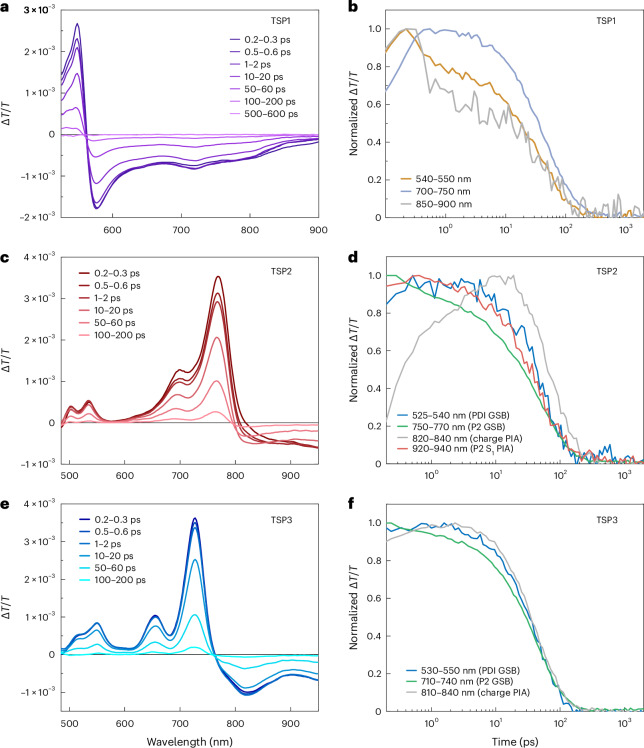
Transient absorption spectroscopy. **a**–**f**, Transient absorption spectra (**a**,**c**,**e**) and extracted kinetics (**b**,**d**,**f**) of TSP1 (**a**,**b**), TSP2 (**c**,**d**) and TSP3 (**e**,**f**) dissolved in toluene. TSP1 was excited at 410 nm, whereas TSP2 and TSP3 were excited at 700 nm.

The predicted differences in the energies and electron–hole interactions of the first excited states of TSP2 and TSP3 can be expected to influence excited-state dynamics. To investigate this, we performed transient-absorption spectroscopy measurements on all three TS polymers in solution (Fig. 5a,c,e), as well as Ref-P1, Ref-P2 and PDI (Supplementary Figs. 18–20) with the extracted kinetics of the three TS polymers shown in (Fig. 5b,d,f).

Figure 5a,b shows the transient absorption spectrum and kinetics of TSP1 in toluene solution (excited at 410 nm), where the conjugated polymer backbone absorbs more strongly than the PDI. The polymer ground-state bleach (GSB) is observed between 520 and 560 nm, with several photoinduced absorption (PIA) bands also present. Although difficult to assign, the PIA bands are distinct from those of Ref-P1, probably indicating the formation of a CT state faster than the 200 fs instrument response of our transient absorption spectroscopy set-up. The CT state then decays rapidly (within approximately hundreds of picoseconds), in clear contrast to the approximately nanosecond lifetime of PDI and Ref-P1 excitons (Supplementary Figs. 18 and 19).

Compared with TSP1, the distinct absorption bands of the polymer donor and PDI acceptor in TSP2 and TSP3 allow for the preferential excitation of either component (PDI excitation is shown in Supplementary Figs. 21 and 22). In the case of TSP3 (Fig. 5e,f), we selectively excite the polymer donor (700 nm pump) to induce electron transfer from the polymer to the PDI. We observe clear GSB signatures for the PDI acceptor (500–570 nm) and polymer donor (620–760 nm), with a new PIA band at 820 nm that is not present in the transient absorption spectra of the individual PDI or Ref-P2 components (Supplementary Figs. 19 and 20). Perylene diimide anions possess absorption bands between 650–850 nm and we thus assign the 820 nm PIA to a negatively charged PDI (referred to here as PIA anion) overlapping with the polymer GSB^37^. There is minimal spectral evolution within the first picosecond (Fig. 5f), and we conclude that, like TSP1, electron transfer has been largely completed within the 200 fs instrument response of our set-up. After a few picoseconds, the observed transient absorption spectrum decays uniformly, with excited-state recombination completed by around 200 ps. Turning to TSP2, with donor excitation at 700 nm (Fig. 5c,d), there are substantial spectral evolutions within the first 10 ps. At 0.2–0.3 ps, we observe the PDI GSB between 500–570 nm and the polymer GSB between 650–800 nm. However, we do not observe a clear PDI anion PIA at this early time, but instead the Ref-P2 singlet (S_1_) exciton PIA at 950 nm (Supplementary Fig. 20). Over picosecond timescales, the polymer S_1_ PIA is lost and the PDI anion PIA at 820 nm grows in. Thus, it seems that electron transfer is much slower in TSP2 than TSP3. Recombination to the ground state then occurs on similar timescales to TSP3.

In TSP2, despite the growth of the PDI anion PIA in the first 10 ps, we note there is no change in the intensity of the PDI GSB. This is surprising because if the electronic ground states of the polymer chain and PDI were electronically decoupled, we would expect the PDI GSB to mirror the rise of the corresponding anion PIA. This suggests that there are sufficiently strong electronic interactions between the polymer electron donor and PDI acceptor for the ground state optical transitions to be directly coupled; pumping one transition directly bleaches the other, similar to how the higher energy local *π*–π* transitions in donor–acceptor co-polymers are bleached when exciting the lowest energy donor to acceptor electronic transition^38^. We propose that this is enabled by the proximity of CT state energies to the local exciton in TSP2, which may enable mixing of the different excited-state wavefunctions. By contrast, the ultrafast appearance of the PDI GSB in TSP3 is consistent with the much faster rate of CT in this material. Thus, it is not possible to separate the ultrafast CT process from any potential coupling of the PDI and polymer ground-state optical transitions for TSP3.

The lifetime of the formed excited state is very short in all through-space polymers, with all excited state species recombining by a few hundred picoseconds. For TSP1, the excited states decay considerably faster than in Ref-P1 and the pristine PDI (Supplementary Figs. 18 and 19). The formed CT state in TSP1 is lower in energy than the excitons in Ref-P1 and PDI, and therefore its faster decay to ground is consistent with the energy gap law^39^. On the other hand, for TSP2 and TSP3, the states that formed after photoexcitation decay on a similar timescale to those of the reference polymer Ref-P2 (Supplementary Fig. 20), which, being a low-gap D–A copolymer, has a shorter excited state lifetime than Ref-P1 but is similar to other D–A polymers in literature^40^. We conclude that the fast excited state recombination in all three through-space polymers results from the inability of charges to separate in the isolated polymer chain and the strong donor–acceptor electronic interaction due to the face-on polymer-PDI orientation. Indeed, this may even represent the intrinsic recombination timescales for spin-singlet CT states strongly localized at the donor:acceptor interface in OPV blends which could previously not be determined as the CT state can obtain longer-range electron-hole separation in a real OPV blend.

## Discussion

The questions of 'what controls interfacial charge transfer?' and, in particular, 'what controls the properties of CT states at a molecular heterojunction?' are of central importance in organic heterojunction devices. It has previously been observed that the molecular orientation of molecules at heterojunctions has a strong impact on interfacial electrostatic interactions and the properties of the CT state^9,12^. In accordance with these results, we find that the energy of the lowest excited CT state strongly depends on which donor moiety is situated closest to the acceptor. Interestingly, we find that the hole preferentially localizes on the DPP moiety as opposed to the BDT moiety, leading to more spatially extended CT states when the DPP moiety is located further away from the PDI. The different spatial distribution of the CT state is expected to lead to different degrees of coupling between CT and the polymer locally excited state, and impact the properties of interfacial CT states.

This picture can be readily reconciled with our kinetic measurements, where relative distance between the electron-withdrawing moiety (DPP) on the polymer and the electron-accepting PDI showed a considerable impact on electron-transfer dynamics. Using a simple kinetic model comprising a ground state, the locally excited and CT states show that increasing the electronic coupling between CT and locally excited states leads both to faster CT state formation and recombination (Supplementary Fig. 23), in agreement with the measured transient absorption spectroscopy results. For example, in TSP3, where the DPP moiety sits directly below the PDI, the electron transfer to a CT state is very rapid (<200 fs). By contrast, TSP2 has BDT directly attached to the PDI, increasing the distance between PDI and DPP, which is associated with a slower (up to 10 ps) forward electron transfer and slower recombination. Although recombination (and separation) are extremely fast in both studied systems, the trend clearly suggests that even greater spatial separation of donor and acceptor units than what was achieved with the current materials could slow down recombination to a useful extent without penalizing charge separation (Supplementary Fig. 23). These results demonstrate that tuning the relative position of the donor and the acceptor moiety can control the kinetics of the electron transfer processes.

In summary, we have demonstrated a synthetic strategy to design and control model interfaces that have allowed us to gain insight into the molecular properties that control CT at molecular donor–acceptor interfaces. Weak coupling between locally excited and CT states can benefit net separation efficiency by reducing both separation and recombination rate constants, so long as the separation rate is fast enough compared with the excited-state lifetime. Optimizing the energy of the CT state to balance those two things can be achieved through manipulating the electron–hole distance via choice of molecular group, and understanding the effect by simulating the ensemble and characterizing the excited states as we describe in this work. Therefore, identifying the preferred localization of the resultant hole and ensuring that is as far away from the acceptor as possible is a design strategy that could result in supressed losses and higher efficiency photon to electric energy conversion in organic heterojunction based optoelectronic devices.

## Methods

### Experimental

#### Characterization and techniques

NMR spectra were recorded on Bruker Avance 400 MHz (^1^H, 400 MHz; ^13^C, 100 MHz) and 600 MHz (^1^H, 600 MHz; ^13^C, 150 MHz) spectrometers. Chemical shifts are reported in *δ* (ppm) relative to the solvent peak: chloroform-*d* (CDCl_3_: ^1^H, 7.26 ppm; ^13^C, 77.16 ppm), tetrahydrofuan-*d*_8_ (THF-*d*_8_: ^1^H, 3.58, 1.72 ppm; ^13^C, 67.21, 25.31 ppm) and dimethyl sulfoxide-*d*_6_ (DMSO-*d*_6_: ^1^H, 2.50 ppm; ^13^C, 39.52 ppm). Mass spectra were obtained using a Waters Xevo G2-S benchtop quadrupole time-of-flight mass spectrometer (equipped with an atmospheric solids analysis probe), a Thermo Finnigan Orbitrap Classic mass spectrometer (using direct injection) or a Bruker UltrafleXtreme matrix-assisted laser desorption/ionization time-of-flight/time-of-flight mass spectrometer at the Yusuf Hamied Department of Chemistry, University of Cambridge. Carbon, hydrogen and nitrogen combustion elemental analyses were obtained on an Exeter Analytical CE-440 elemental analyser, and the results are reported as an average of two samples. Gel permeation chromatography (GPC) was performed on an Agilent Infinity II high-temperature system equipped with an Agilent Olexis Guard column and 2 Agilent Olexis analytical columns. Samples were solubilized in dichlorobenzene at 140 °C using a high-temperature shaking mixer. Samples were filtered through a 10 μm stainless steel frit before injection. Samples were run at 140 °C at 1 ml min^–1^. The instrument was calibrated using Agilent Polystyrene EasiVials and molecular weight averages and dispersity were calculated using Agilent GPC/SEC software. Thermogravimetric analysis was operated under N_2_ atmosphere at a heating rate of 10 °C min^–1^ using a Mettler Toledo TGA/DSC 2 instrument at a gas flow of 125 cm^3^ min^–1^. Flash chromatography was performed using Biotage Isolera Four System and Biotage SNAP/Sfär Silica flash cartridges.

### Materials

Indium tin oxide (ITO) glass substrates were purchased from Kintec. Chloroform and bathocuproine were purchased from Merck and used as received. Poly(3,4-ethylenedioxythiophene) polystyrene sulfonate (PEDOT:PSS; Al4084) was purchased from Ossila. For solution absorption and luminescence measurements, the materials (reference polymers, PDI and through-space polymers) were dissolved in chloroform at 0.2 mg ml^–1^. For thin-film measurements, all materials were dissolved in chloroform at 10 mg ml^–1^.

### Thin film and device fabrication

Indium tin oxide or glass substrates were cleaned by sequential sonication in soapy deionized water, deionized water, acetone and isopropanol. For thin film (photoluminescence, PDS) measurements, the dissolved molecules were spin cast onto the cleaned substrates at 3,000 r.p.m. inside of a glovebox. For electroluminescence measurements, PEDOT:PSS was spin cast at 4,000 r.p.m. onto cleaned ITO substrates and annealed for 15 min at 150 °C; this was followed by the deposition of the dissolved molecules at 3,000 r.p.m. inside of a glovebox. The films were then dried at 80 °C on a hot plate, and bathocuproine (10 nm) and silver (100 nm) were deposited in a Kurt–Lesker evaporator under low pressure (1 × 10^–6^ torr) through shadow masks with 5 mm^2^ area.

### Absorption measurements

Transmission measurements of the solution and thin-films were performed using a Shimadzu UV-2600 spectrophotometer equipped with an ISR-2600Plus integrating sphere. Transmittance spectra were recorded in the 220–1,400 nm wavelength range, and the absorbance was calculated from the logarithm (base ten) of the spectral data.

### Photothermal deflection spectroscopy measurements

Photothermal deflection spectroscopy sensitively measures absorption directly by probing the heating effect in samples upon absorption of light. Films were coated on a Spectrosil fused silica substrate and were immersed in an inert liquid FC-72 Fluorinert. They were then excited with a modulated monochromated light beam perpendicular to the plane of the sample. A combination of a Light Support MKII 100 W Xenon arc source and a CVI DK240 monochromator was used to produce a modulated monochromated light beam. The PDS measurements were acquired by monitoring the deflection of a fixed wavelength (670 nm) diode laser probe beam following absorption of each monochromatic pump wavelength.

### Ambient photoemission spectroscopy measurements

The HOMO energy level of the pristine films was measured by ambient photoemission spectroscopy using an APS04 from KP Technology. The gold probe tip was calibrated using an silver reference sample where the absolute work function of the tip was calculated with respect to the vacuum. The cube root of the photoemission sample was linearly fitted to derive the HOMO energy level of each sample.

### Electroluminescence and photoluminescence measurements

Electroluminescence and photoluminescence spectra were recorded using a Shamrock 303 spectrograph combined with a iDUS InGaAs array detector, which was cooled to –90 °C. The obtained electroluminescence intensity spectra were calibrated with the spectrum from a calibrated halogen lamp. A 473 nm diode laser was used as the excitation source for the photoluminescence spectra, which were measured using the same spectrograph and detector system as for the electroluminescence measurements. Solution absorption spectra were recorded on a Shimadzu UV-1800. Photoluminescence spectra were recorded on an Edinburgh instruments FS5. Photoluminescence quantum yields were determined using an integrating sphere.

### Transient absorption measurements

For the transient absorption measurements, a ytterbium amplifier (PHAROS, Light Conversion), operating at 38 kHz and generating 200 fs pulses centred at 1,030 nm with an output of 14.5 W was used. The pump pulse was provided by an ORPHEUS optical parametric amplifier. The probe is provided by a WL supercontinuum generated in a YAG crystal. After passing through the sample, the probe is imaged using a silicon photodiode array (Stresing S11490).

### Simulations

The model oligomers were generated from concatenated SMILES strings, an initial 3D geometry was generated with ‘smi2sdf’ and then energy minimized with a universal force field (GFN-FF), followed by the tight-binding Hamiltonian (GFN2-xTB) in the ‘xtb’ package^30^. Using the same package, a thermodynamic ensemble (40 configurations) was generated with Langevin (frictional, implicit solvent) molecular dynamics at 300 K using 2 fs time steps with a renormalized hydrogen mass of 4 amu. Dynamics were undertaken for 10 ps, the first 2 ps was discarded as equilibration; then, 40 samples (every 200 fs) were taken from the remaining 8 ps of dynamics. Gaussian 16 (ref. ^41^) was then used to take three gradient geometry optimization steps with density functional theory (B3LYP/3-21G) to get consistent bond lengths, before doing a linear response time-dependent density functional theory (with B3LYP/6-31G*) on the ensembles. Excited-states analysis was performed by using TheoDORE^35^ on the transition density matrices from these linear response TDDFT calculations (Fig. 3f,g). VMD^42^ was used for visualizing the natural transition orbitals, which were generated in Gaussian 16. The global minima structures were those oligomers with minimum energy after a full energy minimization using DFT (B3LYP/6-31G*), starting with seven 1 ps separated samples from the molecular dynamics (to attempt to sample differing conformers). Calculations were undertaken on the Imperial College Research Computing Service.

### Electrochemical measurements

Cyclic voltammetry experiments were recorded using a PalmSens EmStat4S electrochemical analyser. A three-electrode system consisting of a glassy carbon disk (diameter = 1.8 mm) as the working electrode, a platinum wire as an auxiliary electrode and a Ag/AgCl wire as a quasi-reference electrode was used. Cyclic voltammetry experiments were conducted at a scan rate of 100 mV s^–1^. The cyclic voltammogram of the PDI reference molecule was obtained on a solution in dry, degassed THF with *n*-Bu_4_NPF_6_ (0.1 M) as the supporting electrolyte. The cyclic voltammograms of the polymers were obtained on films drop-cast from CHCl_3_ onto the working electrode. Dry degassed MeCN was used, with n-Bu_4_NPF_6_ (0.1 M) as the supporting electrolyte. All experiments were referenced externally to ferrocene.

## Online content

Any methods, additional references, Nature Portfolio reporting summaries, source data, extended data, supplementary information, acknowledgements, peer review information; details of author contributions and competing interests; and statements of data and code availability are available at 10.1038/s41557-024-01578-x.

## Supplementary information

Supplementary Information Supplementary Figs. 1–30, Tables 1–5 and detailed synthetic procedures.

## Source data

Source Data Fig. 1 Source data.

Source Data Fig. 2 Source data.

## Data Availability

The data supporting the findings of this study are available at 10.5281/zenodo.11401564 (ref. ^43^). Source Data are provided with this paper.

## Code availability

All code for data generating and analysis associated with the article is available at 10.5281/zenodo.11401564 (ref. ^43^).
