# Supplementary material for: Synthesis of model heterojunction interfaces reveals molecular-configuration-dependent photoinduced charge transfer
Source: Nat Chem. 2024 Aug 20;16(9):1453–61. doi: 10.1038/s41557-024-01578-x (PMC11374675; doi:10.1038/s41557-024-01578-x)
Supplement: Supplementary file 1 — Supplementary Figs. 1–30, Tables 1–5 and detailed synthetic procedures. [file 41557_2024_1578_MOESM1_ESM.pdf]

nature chemistry

Supplementary information

https://doi.org/10.1038/s41557-024-01578-x

Synthesis of model heterojunction interfaces reveals molecular-configuration-dependent photoinduced charge transfer

In the format provided by the authors and unedited

Table of Contents

1. Synthesis.......... 2

2. NMR Spectra.......... 18

3. Additional optical measurements.......... 36

4. Comparison of experimental and calculated absorption spectra.......... 38

5. Analysis of D:A orientations... 40

6. Thin-film photo and electroluminescence.......... 41

7. Excited state analysis.......... 43

8. Additional transient absorption measurements.......... 52

9. Effect of geometry relaxation on excited state properties.......... 54

10. Kinetic modelling.......... 55

11. Ground state dipole moments.......... 56

12. Frontier molecular orbital energy levels.......... 57

1. Synthesis

Synthesis of Through-Space Monomer 1 & 2 (TS-M1 & TS-M2)

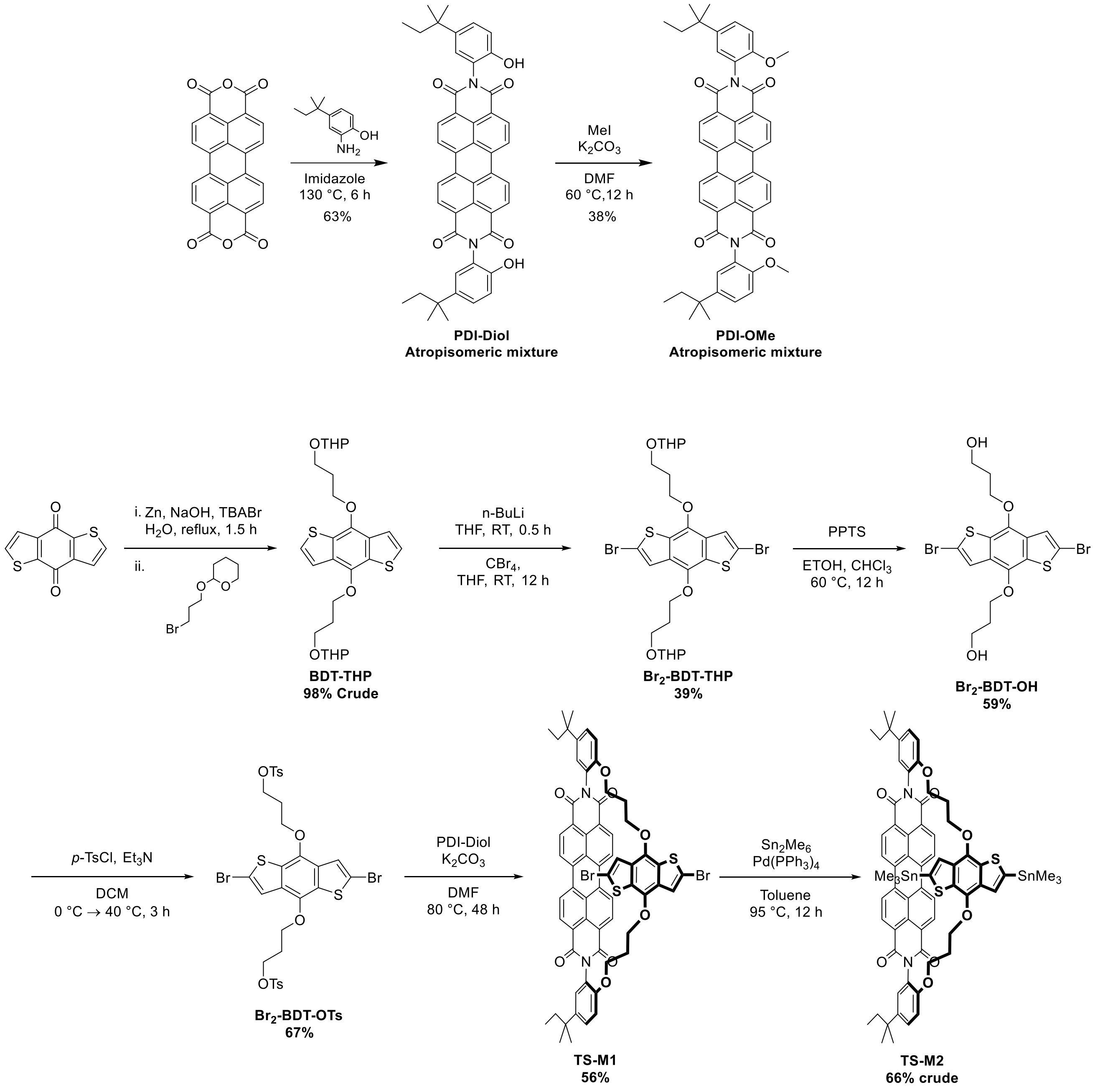

2,9-bis(2-hydroxy-5-(tert-pentyl)phenyl)perylene-3,4,9,10-tetracarboxylic diimide (PDI-Diol)

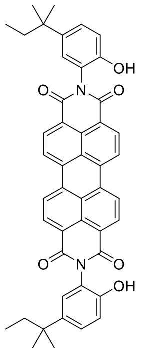

Perylene-3,4,9,10-tetracarboxylic dianhydride (3.00 g, 7.6468 mmol), 2-amino-4-tert-amylphenol (3.43 g, 19.1170 mmol) and imidazole (25 g) were placed in a flask under argon and heated to 130 ºC for 6 h. Next, ethanol (20-30 mL) was added, and it was heated to 90 ºC for 12 h. The reaction mixture was poured into 1M HCl solution. The solids were collected by filtration and washed with 1M HCl (3x). The solids were then dissolved (mostly) in ethyl acetate, dried over $MgSO_4$ and concentrated in vacuo. Next, the residue was sonicated in MeOH, filtered, and washed with Et_2O to afford the product as an atropisomeric mixture and as a fine dark red powder (3.43 g, 4.7984 mmol, 63%). The product was used in the next step without further purification.

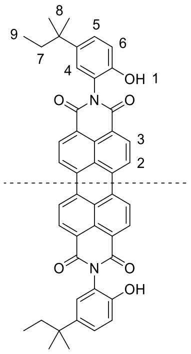

^{1}H NMR (400 MHz, DMSO) δ 9.45 (s, 2H', #1), 9.34 (s, 2H, #1), 8.84 (s, 4H', #2), 8.65 (s, 4H, #2), 8.52 (d, J = 7.9 Hz, 4H', #3), 8.43 (d, J = 7.5 Hz, 4H, #3), 7.33 (s, 2H', #4), 7.28 (d, 2H', #5), 7.24 (d, 2H, #5), 7.22 (s, 2H, #4), 6.95 (d, J = 8.7 Hz, 2H', #6), 6.91 (d, J = 8.4 Hz, 2H, #6), 1.66 – 1.52 (m, 4H'+4H, #7), 1.25 (s, 12H', #8), 1.21 (s, 12H, #8), 0.70 (m, J = 7.0 Hz, 6H'+6H, #9). Minor atropisomer'. **^{13}C NMR{H}** (100 MHz, DMF) δ 163.98, 163.82, 152.65, 152.37, 141.51, 141.28, 135.28, 134.95, 131.71, 131.44, 130.27, 129.76, 129.56, 129.02, 128.12, 127.98, 127.96, 127.07, 126.61, 124.82, 124.76, 124.48, 123.75, 123.54, 117.15, 116.74, 38.20, 38.18, 37.88, 37.82, 29.25, 29.16, 9.97, 9.89. **HRMS** (ASAP-TOF): Calculated for $C_{46}H_{39}N_2O_6^+$: 715.2808. Found *m/z*: 715.2817 $[M+H]^+$.

2,9-bis(2-methoxy-5-(tert-pentyl)phenyl)perylene-3,4,9,10-tetracarboxylic diimide (PDI-OMe)

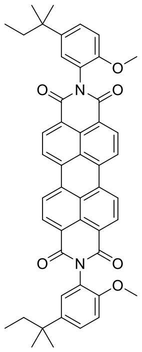

Under argon, PDI-Diol (400 mg, 0.5596 mmol) and K_2CO_3 (309.4 mg, 2.2383 mmol) were stirred in Dry DMF (10 mL) at 80 ºC for 1 h. Next, MeI (0.4 mL, 6.4257 mmol) was added and the reaction was left stirring at 60 ºC for 12 h**.** After cooling to room temperature, the reaction mixture was poured into water (~150 mL) and filtered. The solids were then sonicated in MeOH and collected by filtration again. Next, the crude was purified via silica column chromatography using DCM and EtOAc (slow column first using 100% DCM and then 1-2% EtOAc). The product eluted with 2% EtOAc, concentrated *in vacuo*, sonicated in MeOH, and collected by filtration to afford a red solid material (160 mg, 0.2154 mmol, 38%).

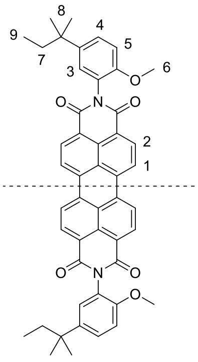

^{1}H NMR (400 MHz, $CDCl_3$) δ 8.75 (d, *J* = 7.9 Hz, 4H, #1), 8.68 (d, *J* = 8.1 Hz, 4H, #2), 7.43 (d, *J* = 8.5 Hz, 2H, #3), 7.23 (d, *J* = 5.1 Hz, 2H, #4), 7.05 (d, *J* = 8.7 Hz, 2H, #5), 3.79 (s, 6H, #6), 1.66 (q, *J* = 7.4 Hz, 4H, #7), 1.31 (s, 12H, #8), 0.78 (t, *J* = 7.3 Hz, 6H, #9). **HRMS** (ASAP-TOF): Calculated for $C_{48}H_{42}N_2O_6^+$: 742.3043. Found *m/z*: 742.3062 $[M]^+$. **^{13}C NMR{H}** (176 MHz, $CDCl_3$) δ 163.39, 163.37, 152.74, 152.70, 142.41, 142.40, 134.98, 134.94, 131.85, 131.81, 130.13, 130.09, 127.87, 127.84, 127.82, 127.75, 126.86, 126.82, 123.87, 123.26, 111.62, 111.58, 56.03, 56.01, 37.58, 37.23, 28.60, 28.59, 9.37. **Anal. Calcd.** for $C_{48}H_{42}N_2O_6$: C, 77.61; H, 5.70; N, 3.77; O, 12.92. Found: C, 76.76; H, 5.50; N, 3.65. (average of two runs).

4,8-bis(2-((oxy)propoxy)tetrahydro-2H-pyran)benzo[1,2-b:4,5-b']dithiophene (BDT-THP)

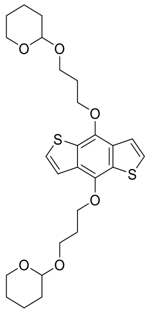

Benzo[1,2-b:4,5-b′]dithiophene-4,8-dione (500 mg, 2.27 mmol), zinc powder (390 mg, 5.90 mmol), sodium hydroxide pellets (1.36 g, 34.30 mmol) and TBABr (380 mg, 0.70 mmol) were combined in a round-bottomed flask, which was subsequently evacuated and backfilled with argon 5 times. Next, degassed water (7 mL) was added to the mixture under argon, leading to a yellow suspension (a slight condensation can be observed on the flask walls). Next, the mixture was refluxed for 1.5 h. This first resulted in a colour change to a deep red suspension and later the mixture became biphasic (dark brown upper layer, faint orange/yellow bottom layer). Then, 2-(3-Bromopropoxy)tetrahydro-2H-pyran (2.08 g, 9.32 mmol) was added in one portion to the mixture, causing the reaction to turn black in colour. The reaction mixture was left refluxing overnight (the dark colour slowly dissipates, and the mixture becomes biphasic again). Next, the mixture was cooled to room temperature, diluted with water and extracted with DCM (2 x). The combined organic layer was then dried over $MgSO_4$ and the solvent was removed under reduced pressure. The THP-protected product was obtained as a transparent, slightly yellow oil (1.13 g, 2.23 mmol, 98% crude) and used as a crude in the subsequent reaction.

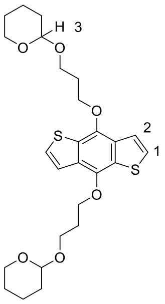

^{1}H NMR (400 MHz, $CDCl_3$) δ 7.51 (d, *J* = 5.5 Hz, 2H, #1), 7.36 (d, *J* = 5.5 Hz, 2H, #2), 4.68 – 4.62 (m, 2H, #3), 4.45 – 4.37 (m, 4H), 4.03 (dt, *J* = 9.8, 6.2 Hz, 2H), 3.87 (ddd, *J* = 11.1, 8.0, 3.2 Hz, 2H), 3.72 (dt, *J* = 9.8, 6.4 Hz, 2H), 3.55 – 3.46 (m, 2H), 2.21 – 2.11 (m, 4H), 1.88 – 1.79 (m, 2H), 1.77 – 1.68 (m, 2H), 1.62 – 1.54 (m, 8H). **HRMS** (ASAP-TOF): Calculated for $C_{26}H_{34}O_6{}^{23}Na_1{}^{32}S_2{}^+$: 529.1689. Found *m/z*: 529.1686 $[M+Na]^+$.

2,6-dibromo(4,8-bis(2-((oxy)propoxy)tetrahydro-2H-pyran))benzo[1,2-b:4,5-b']dithiophene (Br_2-BDT-THP)

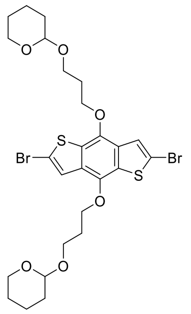

Under argon at -10 ºC, n-BuLi (2 mL, 3.2000 mmol) was added dropwise into a solution of **BDT-THP** (600 mg, 1.1842 mmol) in anhydrous THF (8 mL). The mixture changed colour going from transparent to yellow, green and eventually blue/purple. After addition of n-BuLi, the mixture became thicker and more THF (2 mL) was added to keep the mixture stirring. It was stirred at room temperature for 30 minutes, after which a solution of CBr_4 (1.3352 mg, 4.0262 mmol) in anhydrous THF (1.2 mL) was added in one portion. The reaction mixture was left stirring overnight at room temperature. Next, the reaction mixture was washed with water and brine and re-extracted with diethyl ether (2 x). The combined organic layers were concentrated *in vacuo* and absorbed on silica and a very small amount of K_2CO_3. The crude was purified with silica column chromatography using hexane and DCM (stepwise column; from hexane to 25%, 50% and finally 100% DCM). The product was eluted in the first few fractions, which were concentrated *in vacuo* to afford the product as a transparent oil (306.6 mg, 0.4618 mmol, 39%). The product was used in the subsequent reaction without further purification.

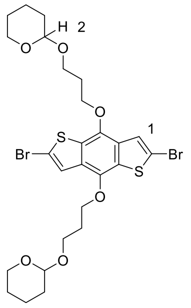

^{1}H NMR (400 MHz, $CDCl_3$) δ 7.50 (s, 2 H, #1), 4.68 – 4.63 (m, 2H, #2), 4.32 (ddd, *J* = 8.9, 6.1, 2.9 Hz, 4H), 4.02 (dt, *J* = 9.9, 6.0 Hz, 2H), 3.89 (ddd, *J* = 11.0, 7.4, 3.5 Hz, 2H), 3.71 – 3.64 (m, 2H), 3.56 – 3.50 (m, 2H), 2.19 – 2.11 (m, 4H), 1.85 (dt, *J* = 9.8, 5.7 Hz, 2H), 1.76 (ddd, *J* = 12.3, 7.6, 4.4 Hz, 2H), 1.66 – 1.55 (m, 8H). **HRMS** (ASAP-TOF): Calculated for $C_{26}H_{33}Br_2O_6S_2^+$: 663.0085. Found *m/z*: 663.0052 $[M+H]^+$.

2,6-dibromo(4,8-bis((oxy)propan-1-ol)tetrahydro-2H-pyran)benzo[1,2-b:4,5-b']dithiophene (Br_2-BDT-OH)

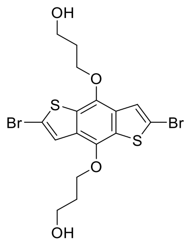

Under argon, Conc. HCl (2 drops) was added to a solution of **Br_2-BDT-THP** (1.3360 g, 2.0106 mmol) and PPTS (1.40 g, 5.5710 mmol) in a mixture of ethanol (50 mL) and $CHCl_3$ (10 mL). The solution was heated to 60 ºC and left stirring for 12 h. Next, the reaction mixture was concentrated *in vacuo* and absorbed onto silica. The crude was purified with silica column chromatography using hexane, DCM and EtOAc (gradual column going very quickly from hexane to 50% and later 100% DCM, and then stepwise to 5%, 10% and 30% EtOAc. The solvents contained a small percentage of Et_3N during column). The product fraction eluted with 30% EtOAc and was concentrated *in vacuo*. Next, it was sonicated in DCM and filtered while cold to afford the product as a white/beige solid (585.2 mg, 1.1793 mmol, 59%).

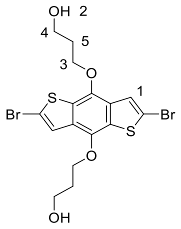

^{1}H NMR (400 MHz, DMSO) δ 7.77 (s, 2H, #1), 4.61 (s, 2H, #2), 4.30 (t, *J* = 6.3 Hz, 4H, #3), 3.64 (m, 4H, #4), 1.91 (p, *J* = 6.2 Hz, 4H, #5). **^{13}C NMR{H}** (101 MHz, DMSO) δ 142.09, 130.80, 130.00, 123.63, 114.87, 71.14, 57.12, 33.10. **HRMS** (ASAP-TOF): Calculated for $C_{16}H_{16}O_4Br_2{}^{32}S_2{}^{+}$: 493.8857. Found *m/z*: 493.8877 $[M]^+$.

2,6-dibromo(4,8-bis(oxy)propyl(*p*-toluenesulfonate))benzo[1,2-b:4,5-b']dithiophene (Br_2-BDT-OTs)

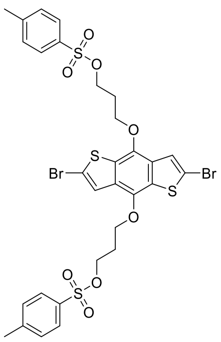

Under argon at -10 ºC, degassed Et_3N (0.16 mL, 1.1587 mmol) was added dropwise into a mixture of **Br_2-BDT-OH** (360 mg, 0.7256 mmol) and *p*-TsCl (318.1 mg, 1.6685 mmol) in anhydrous DCM (6 mL). The mixture allowed to warm to room temperature and sonicated for 2 minutes, after which it was heated for 12 h at 50 ºC (the reaction mixture goes into solution after ~ 1 h). Next, the reaction mixture was concentrated *in vacuo* and absorbed onto silica. The crude was purified with silica column chromatography using hexane and DCM as solvents (stepwise column going from hexane to 50%, 70% and finally 100% DCM). The product fractions were concentrated *in vacuo*, sonicated in MeOH and collected by filtration affording the product as white/beige solids (388.9 mg, 0.4833 mmol, 67%).

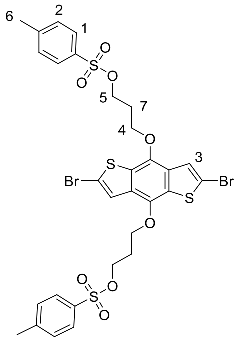

R$_f$ = 0.68 (DCM). **^{1}H NMR** (500 MHz, $CDCl_3$) δ 7.86 – 7.83 (m, 4H, #1), 7.37 (s, 2H, #2), 7.36 (dd, *J* = 8.6, 0.6 Hz, 4H, #3), 4.37 (t, *J* = 6.0 Hz, 4H, #4), 4.26 (t, *J* = 5.9 Hz, 4H, #5), 2.45 (s, 6H, #6), 2.21 (dd, *J* = 12.0, 6.0 Hz, 4H, #7). **^{13}C NMR{H}** (126 MHz, $CDCl_3$) δ 145.11, 142.08, 132.98, 131.17, 130.60, 130.08, 128.07, 123.01, 115.54, 69.31, 66.88, 30.10, 21.83. **HRMS** (ASAP-TOF): Calculated for $C_{30}H_{29}Br_2O_8S_4^+$: 802.9107. Found *m/z*: 802.9112 $[M+H]^+$. **Anal. Calcd.** for $C_{30}H_{28}Br_2O_8S_4$: C, 44.78; H, 3.51; Br, 19.86; O, 15.91; S, 15.94. Found: C, 44.51; H, 3.39; N, <0.2. (average of two runs).

Through-Space Monomer 1 (TS-M1)

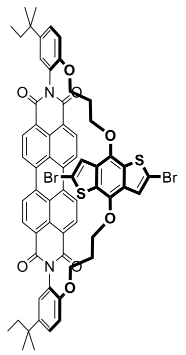

Under argon, K_2CO_3 (280.3 mg, 2.0280 mmol) was added in one portion to a stirring solution of PDI-Diol (271.6 mg, 0.3800 mmol) and Br_2-BDT-OTs (327 mg, 0.4078 mmol) in anhydrous DMF (140 mL). Next, the mixture was heated to 80 ºC and left stirring for 48 h. The reaction mixture was concentrated *in vacuo*, absorbed onto a minimal amount of silica, and purified on silica column chromatography using hexane and DCM (stepwise column going from hexane to 50%, 80% and finally 100% DCM). The product fractions were concentrated *in vacuo*. Next it was sonicated in MeOH and collected by filtration to afford the product as a dark red/pink solid (249 mg, 0.2124 mmol, 56%).

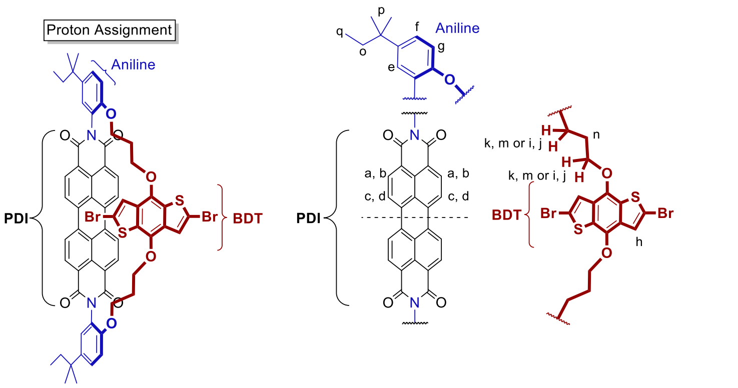

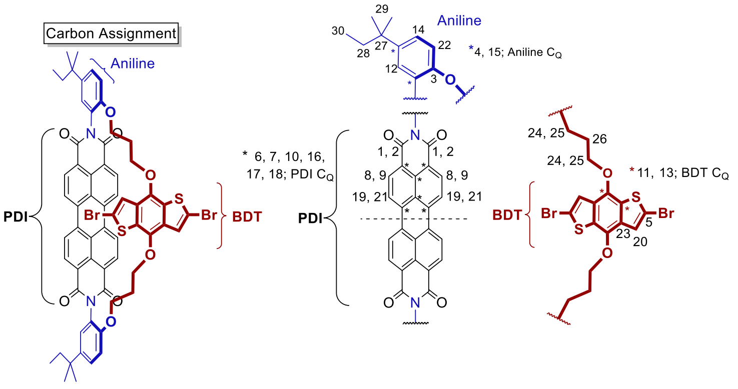

^{1}H NMR (500 MHz, $CDCl_3$) δ 8.66 (d, J = 7.9 Hz, 2H, #a), 8.60 (d, J = 7.9 Hz, 2H, #b), 8.32 (d, J = 8.2 Hz, 2H, #c), 8.23 (d, J = 8.2 Hz, 2H, #d), 7.46 (d, J = 2.4 Hz, 2H, #e), 7.43 (dd, J = 8.5, 2.4 Hz, 2H, #f), 7.06 (d, J = 8.5 Hz, 2H, #g), 6.55 (s, 2H, #h), 4.09 (dt, J = 5.9, 4.9 Hz, 2H, #i), 4.03 (ddd, J = 9.5, 7.2, 4.3 Hz, 2H, #j), 3.76 (dt, J = 8.8, 6.4 Hz, 2H, #k), 3.67 (dt, J = 8.8, 6.4 Hz, 2H, #m), 2.01 – 1.88 (m, 4H, #n), 1.72 (q, J = 7.4 Hz, 4H, #o), 1.37 (s, 12H, #p), 0.82 (t, J = 7.4 Hz, 6H, #q). **^{13}C NMR{H}**

(126 MHz, $CDCl_3$) δ 164.35 (#1), 164.28 (#2), 151.28 (#3), 143.69 (#4, Aniline C_Q), 141.48 (#5), 134.24 (#6, PDI C_Q), 134.07 (#7, PDI C_Q), 131.71 (#8), 131.16 (#9), 129.78 (#10, PDI C_Q), 129.05 (#11, BDT C_Q), 128.27 (#12), 128.06 (#13, BDT C_Q), 127.79 (#14), 126.15 (#15, Aniline C_Q), 126.00 (#16, PDI C_Q), 123.57 (#17, PDI C_Q), 123.25 (#18, PDI C_Q), 122.85 (#19), 122.77 (#20), 122.24 (#21), 115.08 (#22), 114.39 (#23), 71.06 (#24), 67.70 (#25), 37.79 (#26), 37.32 (#27), 31.48 (#28), 28.66 (#29), 9.37 (#30). **HRMS** (ASAP-TOF): Calculated for $C_{62}H_{50}N_2O_8Br_2{}^{32}S_2^+$: 1172.1375. Found *m/z*: 1172.1366 $[M]^+$.

Synthesis of Through-Space Monomer 2 (TS-M2)

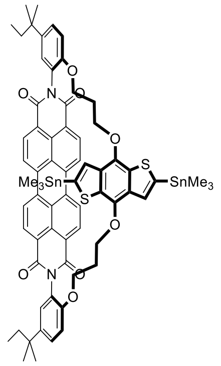

To a microwave vial under argon, dry toluene (12 mL) was added to a mixture of TS-M1 (130 mg, 0.1106 mmol), $Pd(PPh_3)_4$ (12.8 mg, 0.0111 mmol) and hexamethylditin (300 mg, 0.9157 mmol). The reaction mixture was heated to 95 °C and left stirring for 12 h. After cooling the mixture to room temperature, it was poured into stirring MeOH (300 mL) and water (100 mL). The resulting red solids were collected by filtration. Next, the solids were sonicated in hexane and collected by filtration (wash with 50 mL hexane). The crude product (~90% pure by NMR) was obtained as a red solid (98 mg, 0.0730 mmol, 66%) and used in the next step without further purification.

^{1}H NMR (400 MHz, $CDCl_3$) δ 8.54 (d, *J* = 7.5 Hz, 2H), 8.49 (d, *J* = 7.7 Hz, 2H), 8.13 (d, *J* = 8.0 Hz, 2H), 8.07 (d, *J* = 7.9 Hz, 2H), 7.49 – 7.42 (m, 4H), 7.13 (d, *J* = 8.5 Hz, 2H), 6.74 (s, 2H), 4.06 (t, *J* = 5.1 Hz, 4H), 3.89 – 3.70 (m, 4H), 1.99 (dd, *J* = 11.7, 5.8 Hz, 4H), 1.73 (q, *J* = 7.0 Hz, 4H), 1.38 (s, 12H), 0.83 (t, *J* = 7.3 Hz, 6H), 0.50 (s+d, 18H). **HRMS** (ASAP-TOF): Calculated for $C_{68}H_{69}N_2O_8S_2Sn_2^+$: 1343.2528. Found *m/z*: 1343.2479 $[M+H]^+$.

Synthesis of Through-Space Monomer 3 (TS-M3)

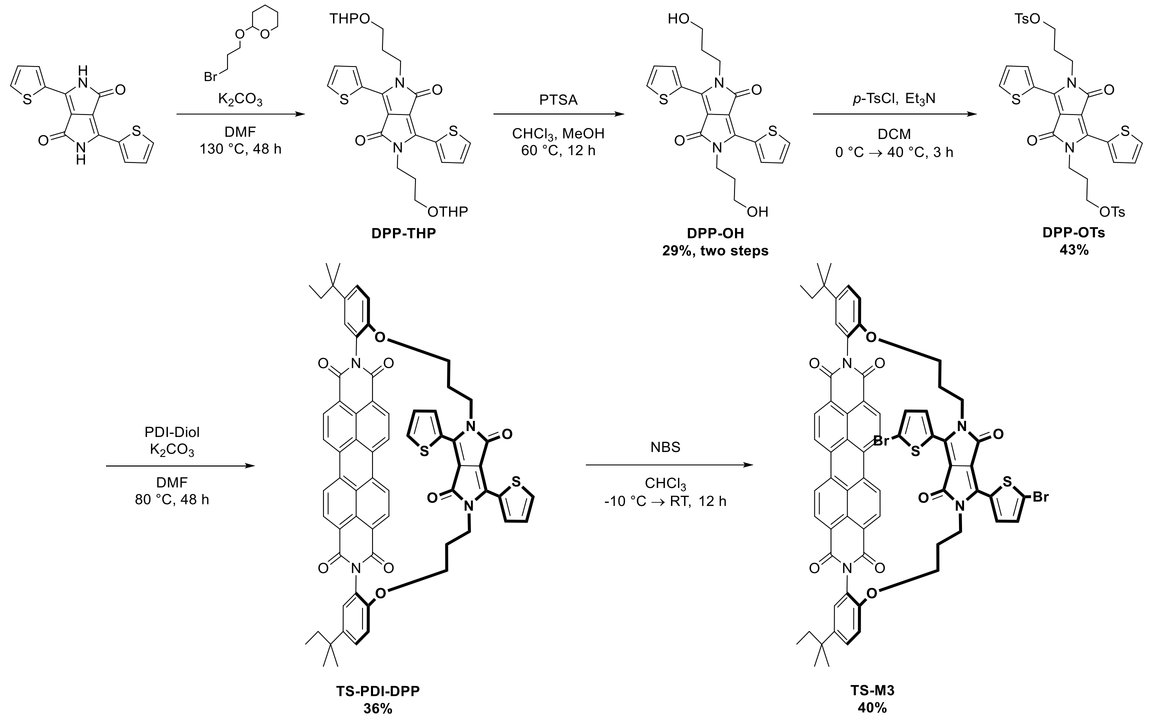

2,5-bis(2-(oxy)propan-1-ol)-3,6-di(thiophen-2-yl)pyrrolo[3,4-c]pyrrole-1,4-dione (DPP-OH)

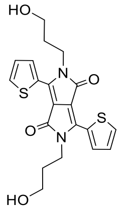

Under argon, a mixture of DPP (1.0 g, 3.3294 mmol), K_2CO_3 (1.4 g, 10.1295 mmol), 2-(3-Bromopropoxy)tetrahydro-2H-pyran (1.69 mL, 10.000 mmol) and dry DMF (20 mL) were stirred at 130 °C for 48 h. The reaction mixture was concentrated *in vacuo*. Water was added to the residue and it was extracted with DCM (3x) and washed with brine (2x). Next, it was dried over $MgSO_4$ and concentrated *in vacuo*. Next, the crude THP-protected product (1.0 g, 1.7101 mmol) and PTSA (1.0 g, 5.8072 mmol) were dissolved into $CHCl_3$ (20 mL) and MeOH (30 mL) and the reaction was left stirring at 45 °C for 4 h under air. The reaction mixture was extracted with DCM (3x) and washed with 1M HCl (4x). The combined organic layer was dried over $MgSO_4$ and concentrated *in vacuo*. The sticky residue was solidified by adding hexane and decanting the liquid (3 x 100 mL). The last step was repeated for diethyl ether (2 x 100 mL). The solids were collected by filtration using a 1:1 mixture of ethyl acetate and diethyl ether. Next, the crude product was purified by trituration from acetonitrile (the solids were dissolved in 500 mL boiling acetonitrile. Charcoal was added, and the solution was filtered through celite. The solution was then reduced to 100 mL and left cooling to room temperature overnight,

allowing solid product to crash out). The product was obtained as a dark pink/red coloured solid (402.1 mg, 0.9655 mmol, 29%).

^{1}H NMR (700 MHz, DMSO) δ 8.78 (d, *J* = 3.3 Hz, 2H), 8.10 (d, *J* = 4.7 Hz, 2H), 7.44 – 7.35 (dd, *J* = 4.7 Hz, 3.3 Hz, 2H), 4.61 (t, *J* = 5.1 Hz, 2H), 4.10 – 4.02 (t, 4H), 3.50 (dd, *J* = 11.5, 6.0 Hz, 4H), 1.86 – 1.74 (p, 4H). **^{13}CNMR** (176 MHz, DMSO) δ 160.86 (C=O), 139.77 (C_Q), 134.90 (CH), 133.36 (CH), 129.63 (C_Q), 129.09 (CH), 107.09 (C_Q), 58.89 (CH_2), 39.75 (CH_2), 33.10(CH_2). **HRMS** (ASAP-TOF): Calculated for $C_{20}H_{21}N_2O_4S_2^+$: 417.0943. Found *m/z*: 417.0953 $[M+H]^+$. **Anal. Calcd.** for $C_{20}H_{20}N_2O_4S_2$: C, 57.67; H, 4.84; N, 6.73; O, 15.36; S, 15.39. Found: C, 56.34; H, 4.89; N, 6.26. (average of two runs).

2,5-bis(2-(oxy)propyl(*p*-toluenesulfonate))-3,6-di(thiophen-2-yl)pyrrolo[3,4-c]pyrrole-1,4-dione (DPP-OTs)

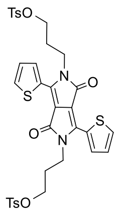

Under argon, Et_3N (0.485 mL) was added dropwise to a mixture of DPP-OH (350 mg, 0.8403 mmol) and *p*-TsCl (352 mg, 1.8487 mmol) in dry DCM (20 mL) and it was stirred for 1 h at room temperature and 3 h at 40 °C. The reaction mixture was extracted with DCM (3x) and washed with water (1x), 1M HCl (1x) and brine (2x). The combined organic layers were dried over $MgSO_4$ and concentrated *in vacuo*. The product was further purified via silica column chromatography (using 5% EtOAc in DCM) and the resulting solids were sonicated in methanol to afford the product as a dark red solid (261.9 mg, 0.3613 mmol, 43% yield).

^{1}H NMR (600 MHz, $CDCl_3$) δ 8.77 (dd, *J* = 3.9, 1.1 Hz, 2H), 7.78 (d, *J* = 8.3 Hz, 4H), 7.66 (dd, *J* = 5.0, 1.1 Hz, 2H), 7.31 (d, *J* = 8.0 Hz, 4H), 7.29 (dd, *J* = 5.0, 3.9 Hz, 2H), 4.16 (t, *J* = 6.2 Hz, 4H), 4.15 – 4.12 (t, 4H), 2.43 (s, 6H), 2.15 – 2.09 (m, 4H). **^{13}C NMR{H}** (176 MHz, CDCl3) δ 161.37, 145.04, 139.91, 135.36, 133.01, 131.32, 130.03, 129.37, 128.96, 128.08, 107.78, 68.02, 39.18, 29.48, 21.82. **HRMS** (ASAP-TOF): Calculated for $C_{34}H_{33}N_2O_8S_4^+$: 725.1120. Found *m/z*: 725.1085 $[M+H]^+$. **Anal. Calcd.** for $C_{34}H_{32}N_2O_8S_4$: C, 56.34; H, 4.45; N, 3.86; O, 17.66; S, 17.69. Found: C, 55.29; H, 4.26; N, 3.73. (average of two runs).

TS-PDI-DPP

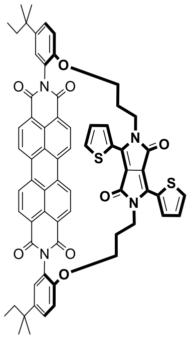

Under argon, K_2CO_3 (259.3 mg, 1.8760 mmol) was added in one portion to a stirring solution of PDI-Diol (165 mg, 0.2308 mmol) and DPP-OTs (170 mg, 0.2345 mmol) in anhydrous DMF (40 mL). Next, the mixture was heated to 80 ºC and left stirring for 48 h. The reaction mixture was concentrated *in vacuo*. DCM (~30 mL) and water (~30 mL) were added to the residue and it was extracted with DCM (3x) and washed with brine (1x). The combined organic layer was dried over $MgSO_4$ and concentrated *in vacuo*. The solids were absorbed onto a minimal amount of silica and purified via silica column chromatography (100% hexane → 100% DCM → 20% EtOAc) to afford the product as a dark red/pink solid (90 mg, mmol, 36% yield).

^{1}H NMR (400 MHz, CDCl3) δ 8.78 (d, J = 8.0 Hz, 2H), 8.46 (d, J = 8.2 Hz, 2H), 8.33 (dd, J = 3.9, 1.0 Hz, 2H), 8.20 (d, J = 8.0 Hz, 2H), 8.12 (d, J = 8.2 Hz, 2H), 7.56 (dd, J = 5.0, 1.0 Hz, 2H), 7.47 – 7.41 (m, 4H), 7.21 (dd, J = 4.9, 4.0 Hz, 2H), 7.15 (d, J = 8.3 Hz, 2H), 4.15 – 4.00 (m, 4H), 3.42 – 3.28 (m, 2H), 3.25 – 3.10 (m, 2H), 1.93 (dt, J = 14.3, 5.7 Hz, 4H), 1.71 (q, J = 7.4 Hz, 4H), 1.37 (s, 12H), 0.81 (t, J = 7.4 Hz, 6H). **^{13}C NMR{H}** (176 MHz, CDCl3) δ 164.02, 163.98, 160.79, 151.54, 144.52, 139.33, 134.53, 134.46, 134.38, 132.13, 131.18, 131.06, 129.78, 129.37, 128.69, 128.36, 127.96, 126.41, 126.26, 124.06, 123.52, 123.26, 122.57, 116.90, 106.86, 68.78, 41.57, 37.84, 37.25, 28.64, 28.63, 28.39, 9.38. **HRMS** (ASAP-TOF): Calculated for $C_{66}H_{53}N_4O_8S_2^+$: 1093.3305. Found *m/z*: 1093.3303 $[M]^+$. **Anal. Calcd.** for $C_{66}H_{54}N_4O_8S_2$: C, 72.38; H, 4.97; N, 5.12; O, 11.69; S, 5.85. Found: C, 71.21; H, 4.79; N, 5.04. (average of two runs).

Through-Space Monomer 3 (TS-M3)

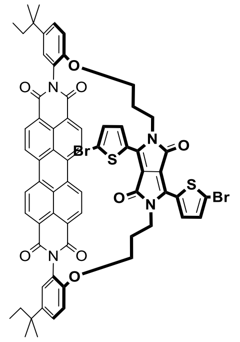

Under air, at -10 °C, NBS (101.1 mg, 0.5680 mmol) was added portionwise over 1 h to a solution of TS-PDI-DPP (293 mg, 0.2675 mmol) in $CHCl_3$ (7 mL). The salt/ice bath was removed, and the reaction was left stirring for 12 h at room temperature. The reaction was quenched using a sodium thiosulfate solution and extracted (2x) with DCM. Next, the organic layer was dried over $MgSO_4$ and concentrated *in vacuo*. The crude product was purified via silica column chromatography (100% hexane → 100% DCM → 3% EtOAc). The product fractions were combined and concentrated *in vacuo*. The resulting solids were sonicated in hexane and collected by filtration. Next, it was further purified using silica column chromatography for a second time (100% DCM → 0.5% Acetone). The product fractions were combined and concentrated *in vacuo*. The less pure fractions containing product were sonicated separately in hot acetone and collected by filtration. Both parts were then combined to afford a dark pink/red coloured solid product (133 mg, 0.1061 mmol, 40% yield).

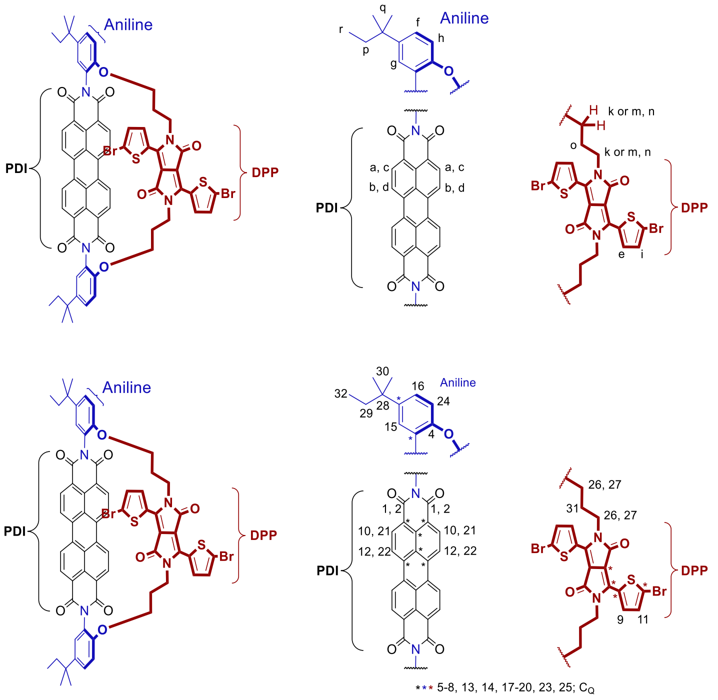

^{1}H NMR (500 MHz, $CDCl_3$) δ 8.76 (d, J = 8.0 Hz, 2H, #a), 8.45 (d, J = 8.2 Hz, 2H, #b), 8.37 (d, J = 8.0 Hz, 2H, #c), 8.21 (d, J = 8.2 Hz, 2H, #d), 7.95 (d, J = 4.2 Hz, 2H, #e), 7.49 – 7.41 (dd, J = 8.7, 2.4

Hz, 2H, #f; d, 2H, #g), 7.12 (dd, J = 8.7, 0.9 Hz, 2H, #h; d, 2H, #i), 4.15 – 3.99 (m, 4H, #k), 3.41 (ddd, J = 14.6, 12.6, 4.9 Hz, 2H, #m), 3.09 (ddd, J = 14.7, 12.3, 5.0 Hz, 2H, #n), 2.03 – 1.77 (p, 4H, #o), 1.71 (q, J = 7.4 Hz, 4H, #p), 1.37 (s, 12H, #q), 0.82 (t, J = 7.4 Hz, 6H, #r). **^{13}C NMR{H}** (126 MHz, $CDCl_3$) δ 163.93 (#1, C=O), 163.90 (#2, C=O), 160.42 (#3, C=O), 151.42 (#4), 144.31 (#5, C_Q), 138.00 (#6, C_Q), 134.60 (#7, C_Q), 134.56 (#8, C_Q), 133.94 (#9), 132.04 (#10), 131.71 (#11), 131.11 (#12), 130.80 (#13, C_Q), 129.93 (#14, C_Q), 128.34 (#15), 127.95 (#16), 126.39 (#17, C_Q), 126.02 (#18, C_Q), 124.06 (#19, C_Q), 123.76 (#20, C_Q), 123.30 (#21), 122.50 (#22), 119.87 (#23, C_Q), 116.09 (#24), 107.00 (#25, C_Q), 67.97 (#26), 41.46 (#27), 37.82 (#28), 37.25 (#29), 28.62 (#30), 28.41 (#31), 9.40 (#32). **HRMS** (ASAP-TOF): Calculated for $C_{66}H_{53}N_4O_8S_2Br_2^+$: 1251.1672. Found m/z: 1251.1686 $[M+H]^+$.

Synthesis of Through-Space Polymers

TSP1

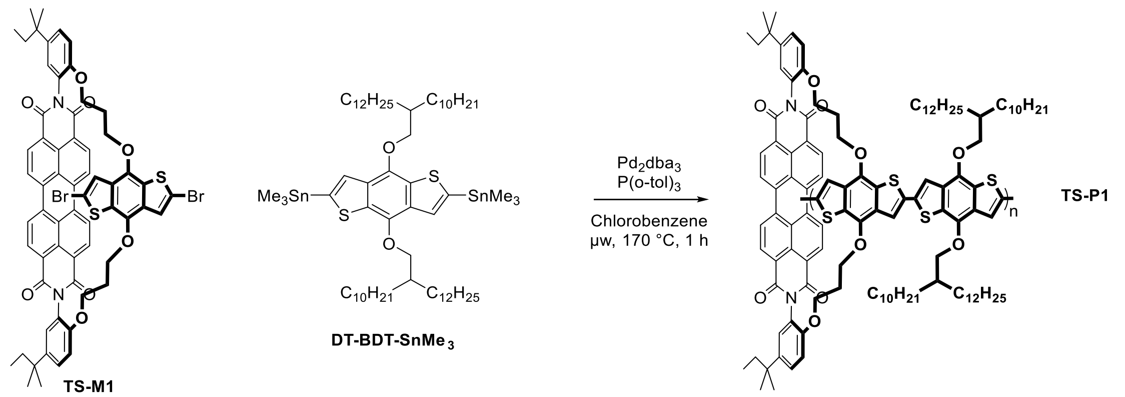

Under argon, dry chlorobenzene (2.0 mL) was added to a mixture of TS-M1 (100 mg, 0.0851 mmol), DT-BDT-$SnMe_3$ (95.4 mg, 0.0851 mmol), Pd_2dba_3 (1.6 mg, 0.0017 mmol) and $P(o\text{-}tol)_3$ (2.1 mg, 0.0068 mmol), and it was stirred under microwave irradiation at 100 °C for 1 min., 120 °C for 5 min., 140 °C for 5 min., and finally 170 °C for 1 h. After cooling down, the reaction mixture was precipitated into stirring methanol (~100 mL) giving small red flakes of polymer. The crude polymer was purified with soxhlet extractions using acetone, hexane, heptane, ethyl acetate and finally chloroform. The chloroform fraction was re-precipitated into stirring methanol and collected by filtration to afford the desired polymer as a dark red/orange plastic (148.4 mg, 0.0777 mmol, 91%). M_n = 19.7 kDa, PDI = 2.30.

TSP2

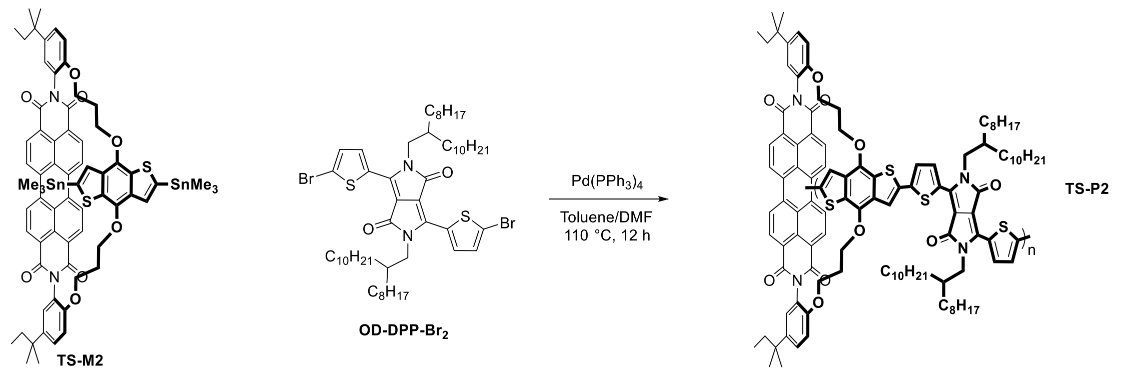

Under argon, dry toluene (1.35 mL) and dry DMF (0.12 mL) were added to a mixture of TS-M2 (80.0 mg, 0.0599 mmol), OD-DPP-Br_2 (95.4 mg, 0.0599 mmol) and $Pd(PPh_3)_4$ (1.6 mg, 0.0014 mmol), and it was stirred at 110 °C for 12 h. After cooling down, the reaction mixture was precipitated into stirring methanol (~100 mL) giving small purple-blue flakes of polymer. The crude polymer was purified with soxhlet extractions using acetone, hexane, heptane, ethyl acetate, 1,2-dimethoxyethane and finally chloroform. The chloroform fraction was re-precipitated into stirring methanol and collected by filtration to afford the desired polymer as a dark blue/purple plastic (71.5 mg, 0.0381 mmol, 64%). M_n = 11.6 kDa, PDI = 1.7.

TSP3

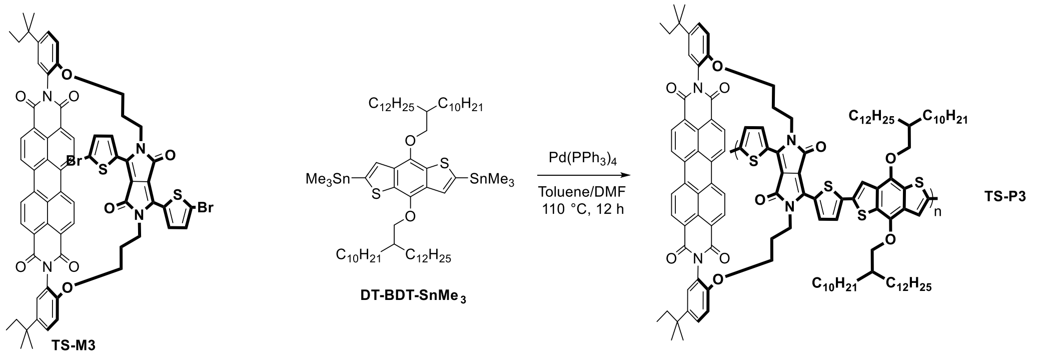

Under argon, dry toluene (1.35 mL) and dry DMF (0.12 mL) were added to a mixture of TS-M3 (80.0 mg, 0.0638 mmol), DT-BDT-$SnMe_3$ (78.0 mg, 0.0638mmol) and $Pd(PPh_3)_4$ (1.6 mg, 0.0014 mmol), and it was stirred at 110 °C for 12 h. After cooling down, the reaction mixture was precipitated into stirring methanol (~100 mL) giving green flakes of polymer. The crude polymer was purified with soxhlet extractions using acetone, hexane and finally chloroform. The chloroform fraction was re-precipitated into stirring methanol and collected by filtration to afford the desired polymer as a green plastic (112 mg, 0.0563 mmol, 88%). M_n = 72.5 kDa, PDI = 3.68.

Synthesis of Reference Polymers

Ref-P1 Synthesis

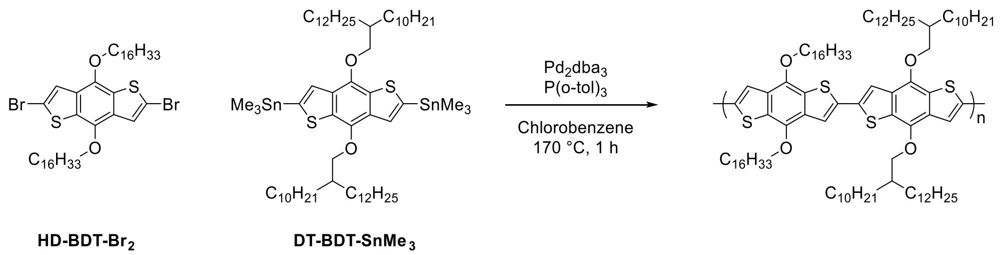

Under argon, dry chlorobenzene (1.8 mL) was added to a mixture of HD-BDT-Br_2 (65 mg, 0.07841 mmol), DT-BDT-$SnMe_3$ (95.8 mg, 0.07841 mmol), Pd_2dba_3 (1.5 mg, 0.0016 mmol) and P(o-tol)$_3$ (1.9 mg, 0.0031 mmol), and it was stirred under microwave irradiation at 100 °C for 1 min., 120 °C for 5 min., 140 °C for 5 min., and finally 170 °C for 1 h. After cooling down, the reaction mixture was precipitated into stirring methanol (~100 mL) giving orange flakes of polymer. The crude polymer was purified with soxhlet extractions using acetone and finally hexane and chloroform. The combined hexane and chloroform fraction was re-precipitated into stirring methanol and collected by filtration to afford the desired polymer as a dark red/orange plastic (126.3 mg, 0.0699 mmol, 89%). M_n = 63.1 kDa, Mw = 232.5 kDa, PDI = 3.68.

Ref-P2 Synthesis

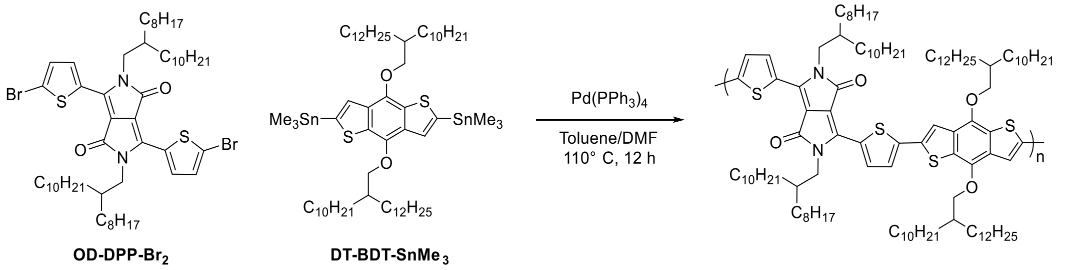

Under argon, dry toluene (2.25 mL) and dry DMF (0.2 mL) were added to a mixture of OD-DPP-Br_2 (100 mg, 0.0981 mmol), DT-BDT-$SnMe_3$ (119.8 mg, 0.0981 mmol) and $Pd(PPh_3)_4$ (2.68 mg, 0.0023 mmol), and it was stirred in an oil bath at 110 °C for 12 h. After cooling down, the reaction mixture was precipitated into stirring methanol (~100 mL) giving green-blue flakes of polymer. The crude polymer was purified with soxhlet extractions using acetone, ethyl acetate and finally hexane. The hexane fraction was re-precipitated into stirring methanol and collected by filtration to afford the desired polymer as a sticky green-blue plastic (151.5 mg, 0.0863 mmol, 88%). M_n = 58.2 kDa, kDa, PDI = 3.78.

2. NMR Spectra

2,9-bis(2-hydroxy-5-(tert-pentyl)phenyl)perylene-3,4,9,10-tetracarboxylic diimide (PDI-Diol)

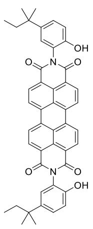

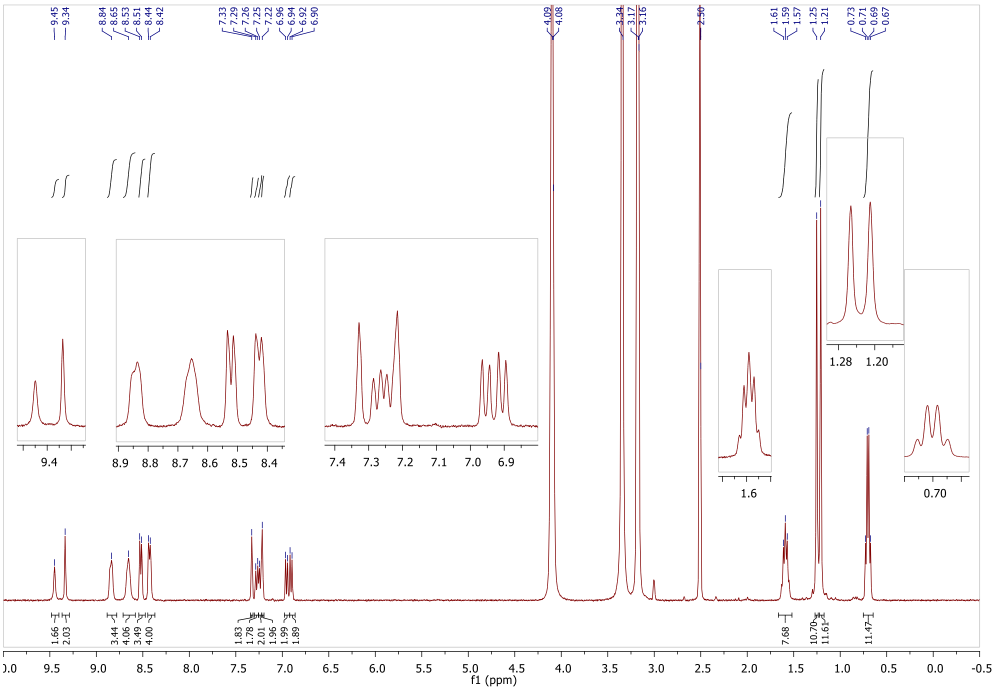

2,9-bis(2-methoxy-5-(tert-pentyl)phenyl)perylene-3,4,9,10-tetracarboxylic diimide (PDI-OMe)

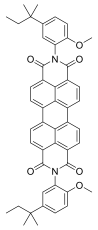

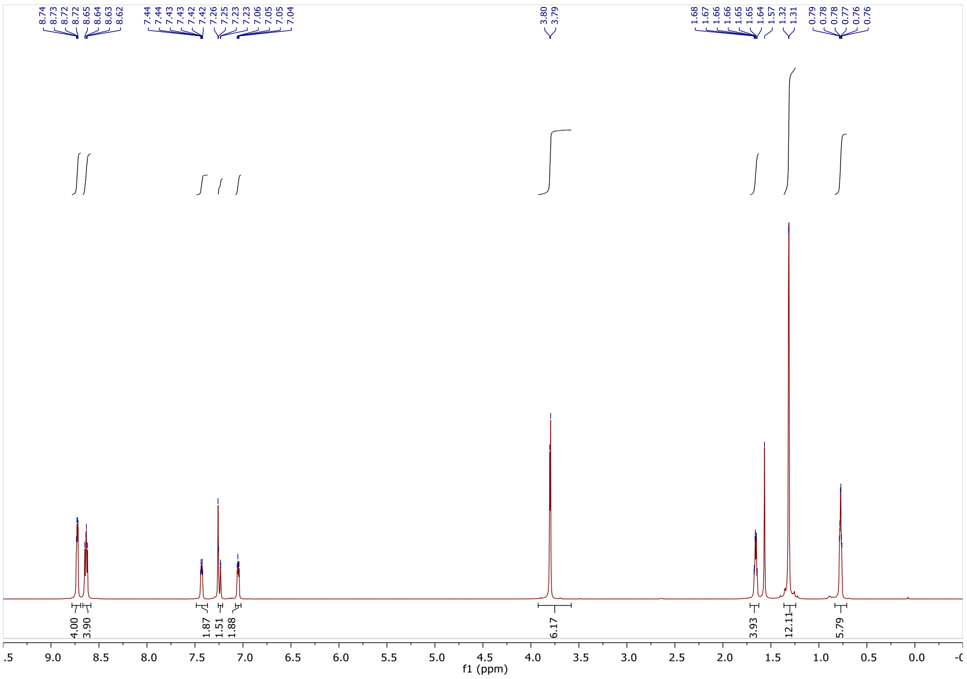

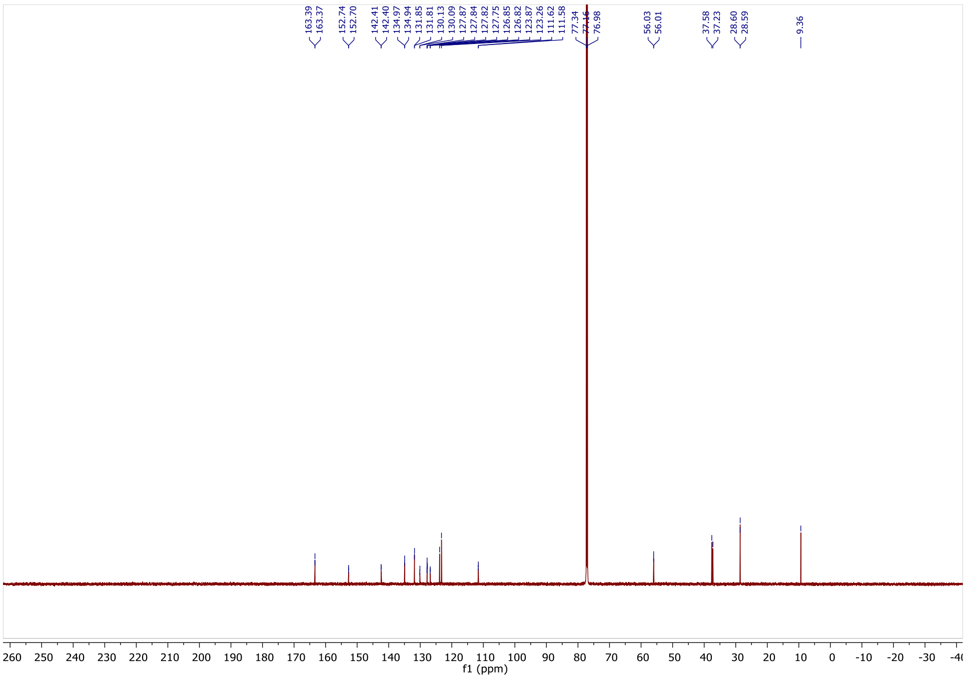

4,8-bis(2-((oxy)propoxy)tetrahydro-2H-pyran)benzo[1,2-b:4,5-b']dithiophene (BDT-THP)

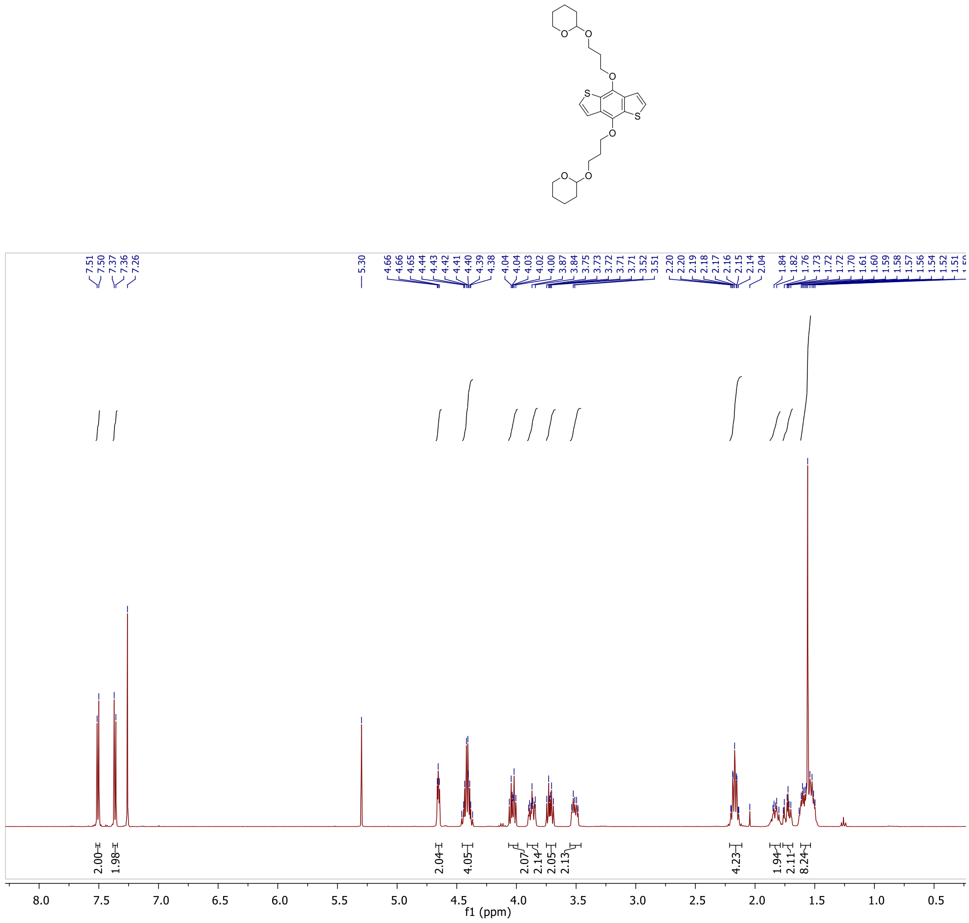

S
O
2.00
1.98
2.04
4.05
2.07
2.14
2.05
2.13
4.23
1.94
2.11
8.24
8.0 7.5 7.0 6.5 6.0 5.5 5.0 4.5 4.0 3.5 3.0 2.5 2.0 1.5 1.0 0.5
f1 (ppm)

2,6-dibromo(4,8-bis(2-((oxy)propoxy)tetrahydro-2H-pyran))benzo[1,2-b:4,5-b']dithiophene (Br_2-BDT-THP)

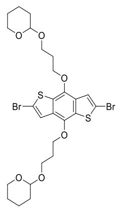

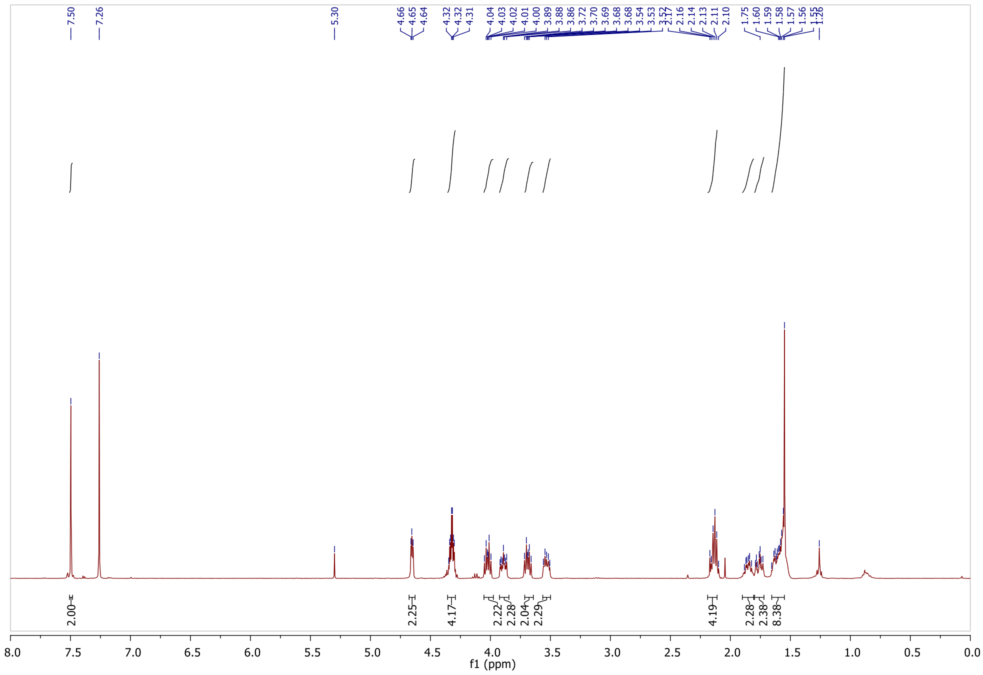

2,6-dibromo(4,8-bis((oxy)propan-1-ol)tetrahydro-2H-pyran)benzo[1,2-b:4,5-b']dithiophene (Br_2-BDT-OH)

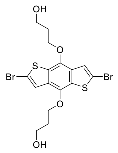

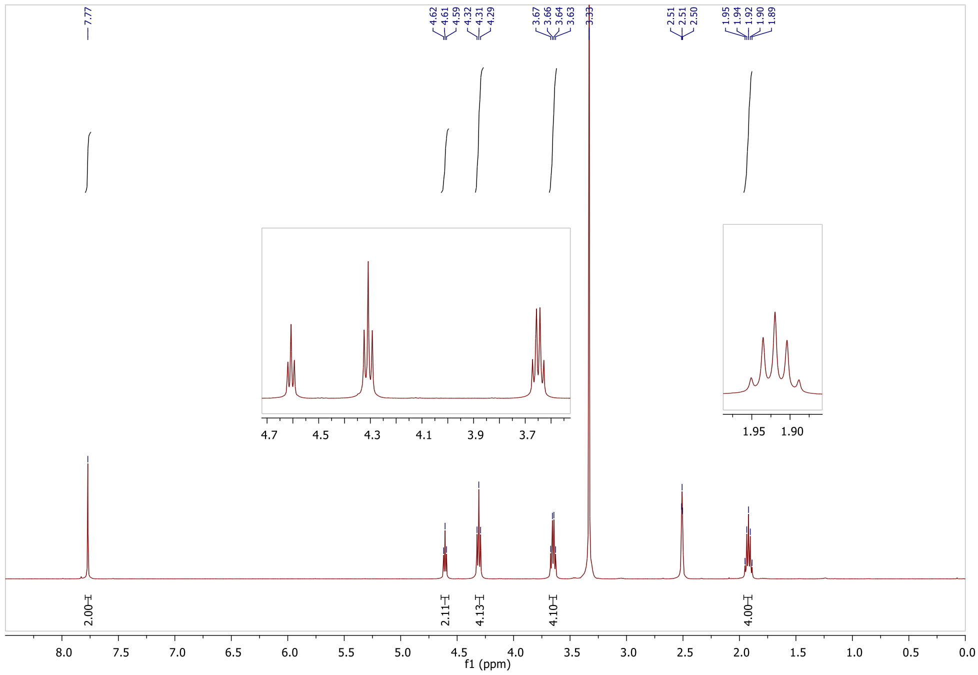

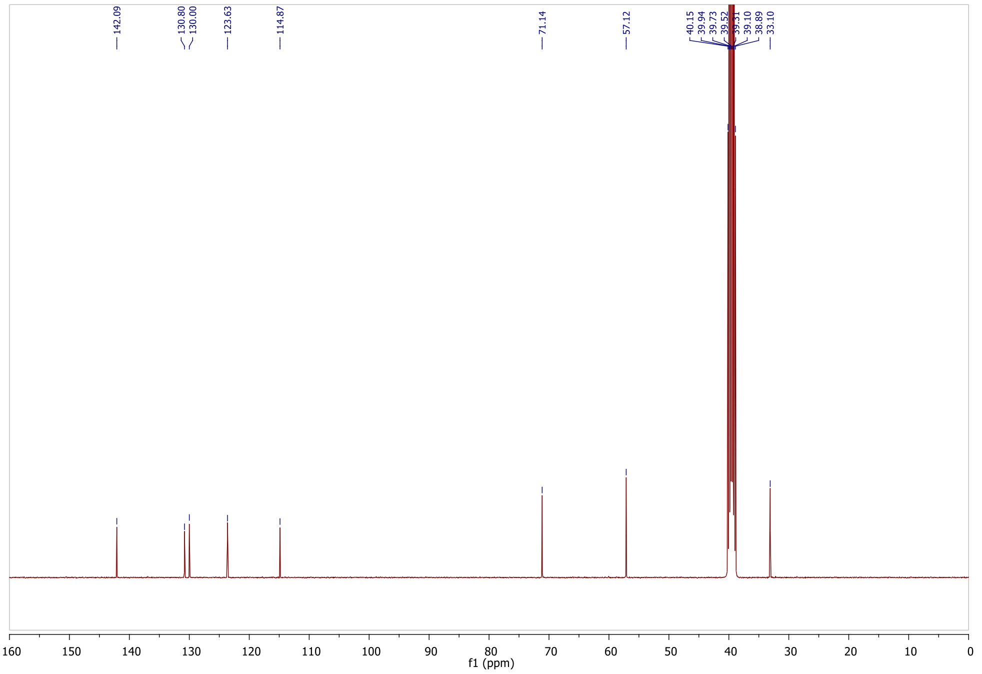

2,6-dibromo(4,8-bis((oxy)propyl(*p*-toluenesulfonate)))benzo[1,2-b:4,5-b']dithiophene (Br_2-BDT-OTs)

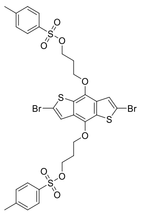

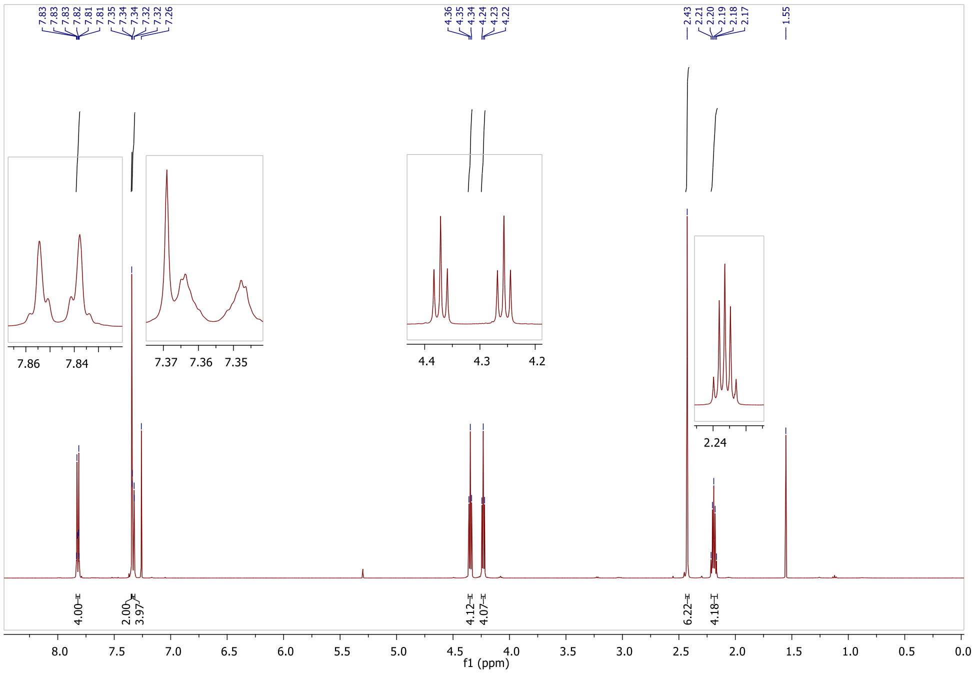

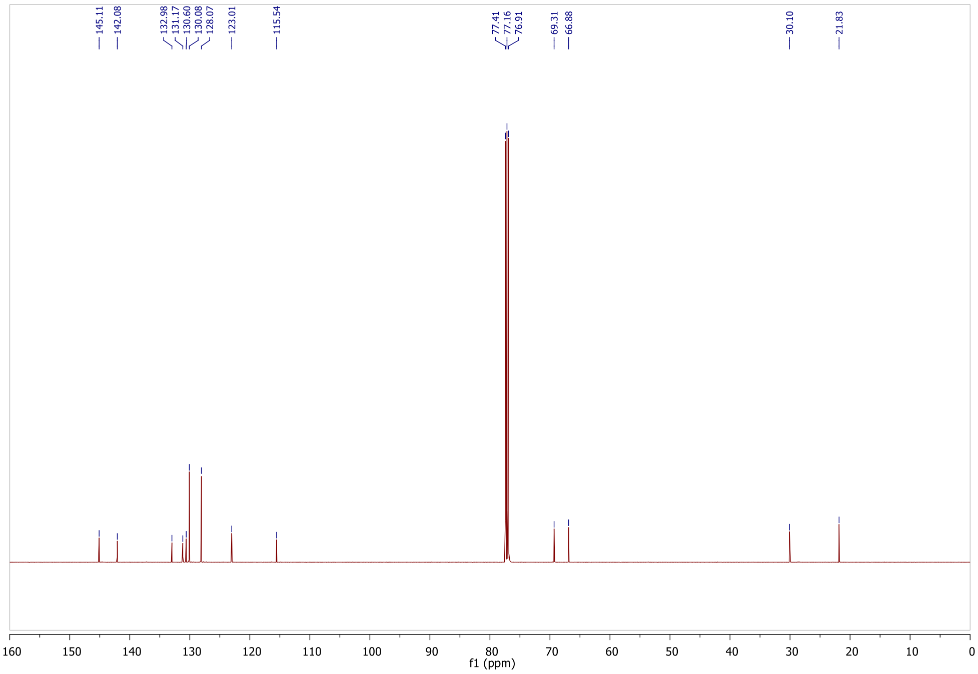

Through-Space Monomer 1 (TS-M1)

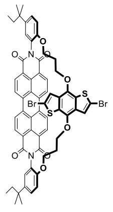

O
N
Br
S
S
Br

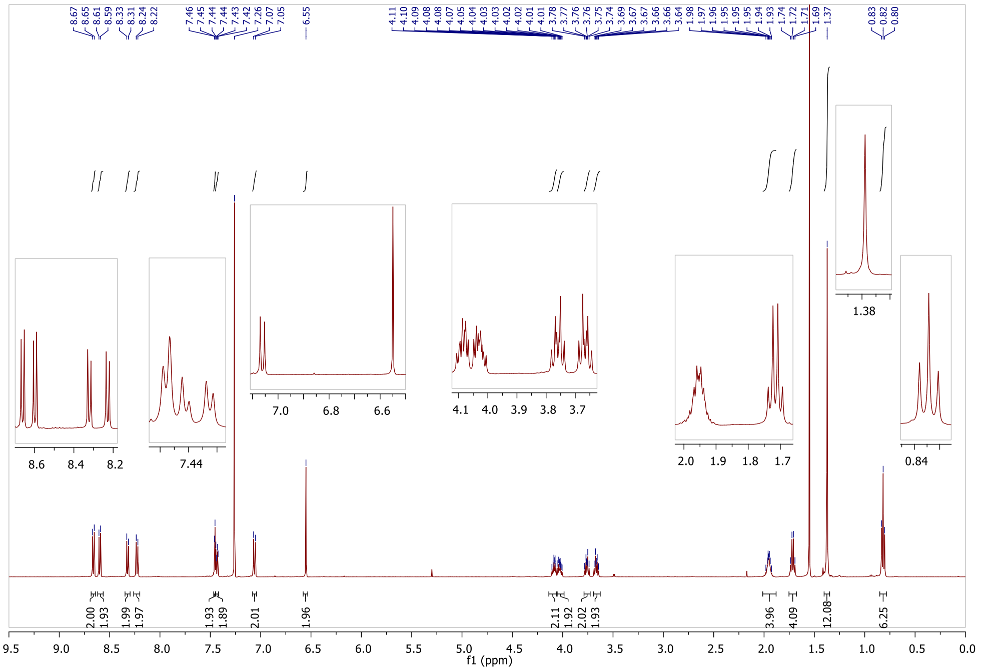

8.67
8.65
8.61
8.59
8.33
8.31
8.24
8.22
7.46
7.45
7.44
7.44
7.43
7.42
7.26
7.07
7.05
6.55
1.37
0.83
0.82
0.80
2.00
1.93
1.99
1.97
1.93
1.89
2.01
1.96
2.11
1.92
2.02
1.93
3.96
4.09
12.08
6.25
f1 (ppm)

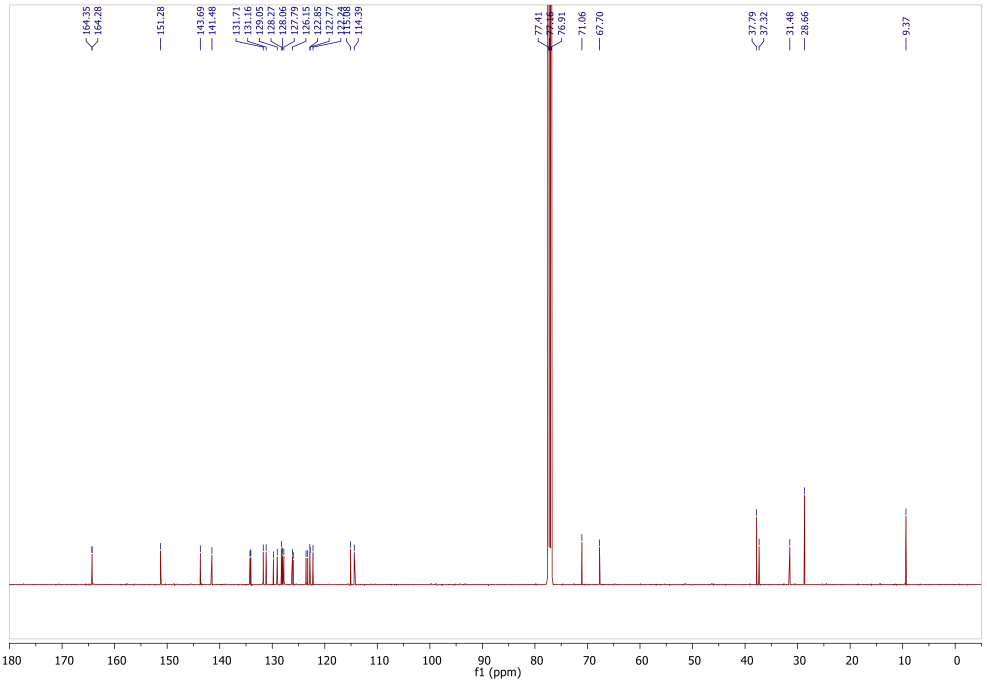

164.35
164.28
151.28
143.69
141.48
71.06
67.70
37.79
37.32
31.48
28.66
9.37
f1 (ppm)

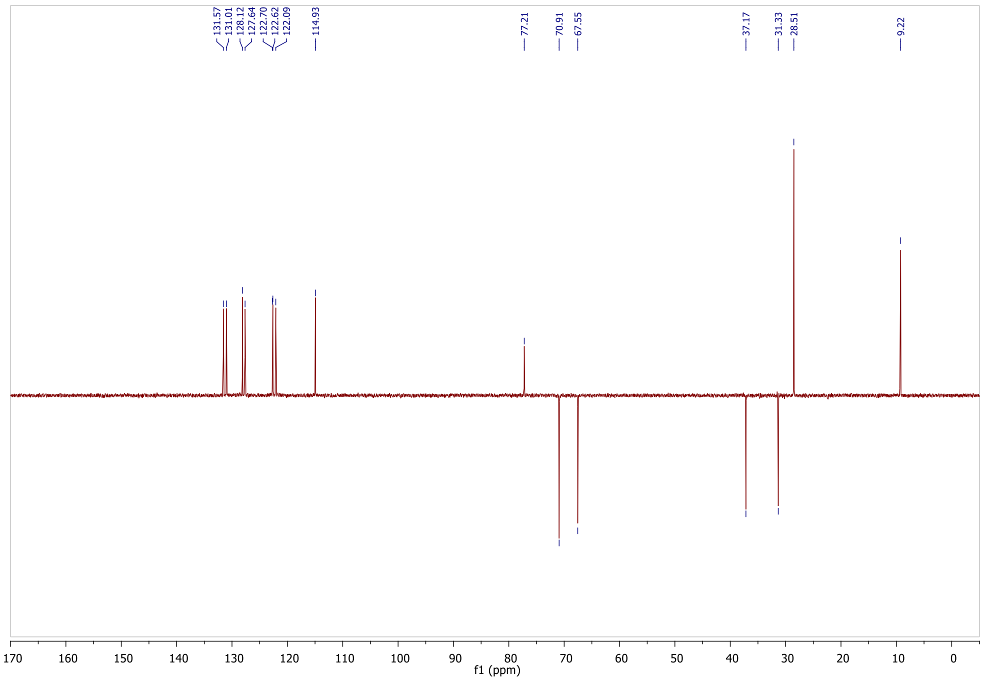

^{13}C DEPT spectrum of TS-M1.

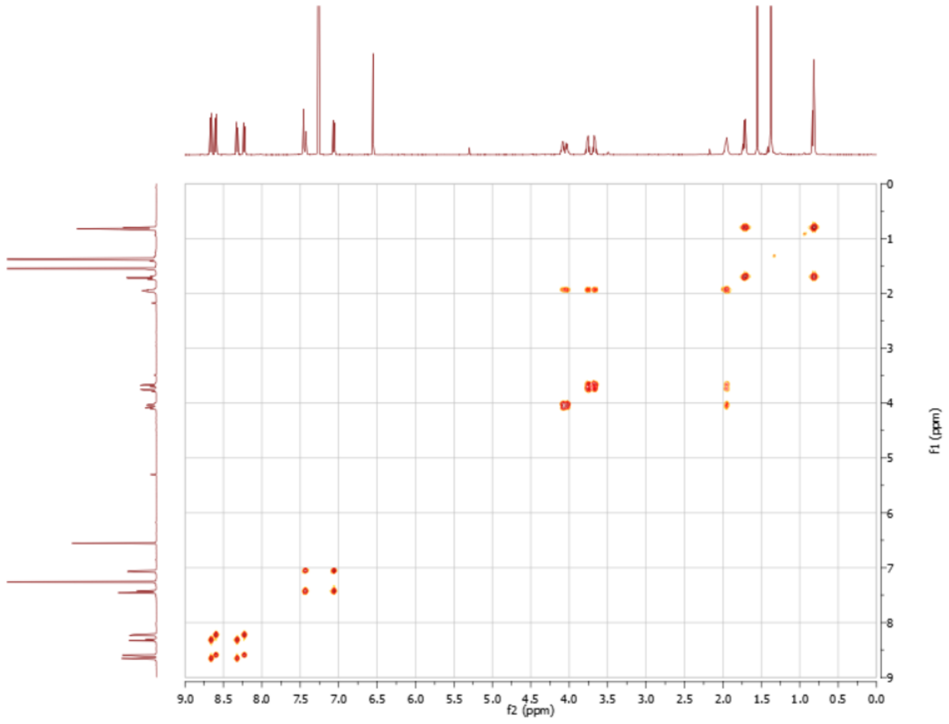

^{1}H-^{1}H COSY spectrum of TS-M1.

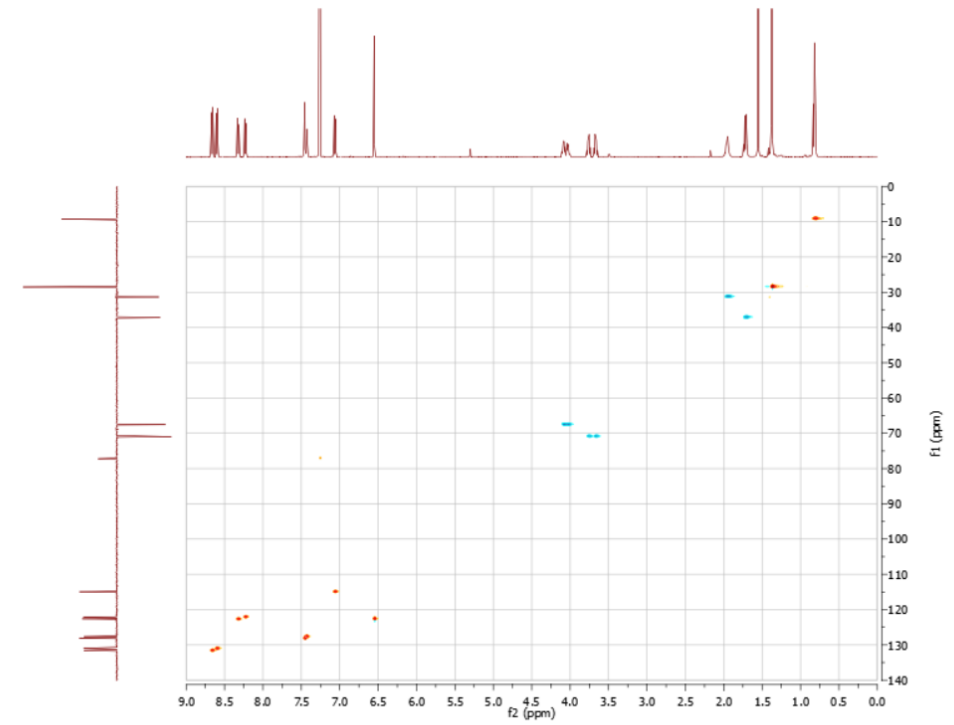

HSQC spectrum of TS-M1, with ^{13}C DEPT spectrum on y-axis.

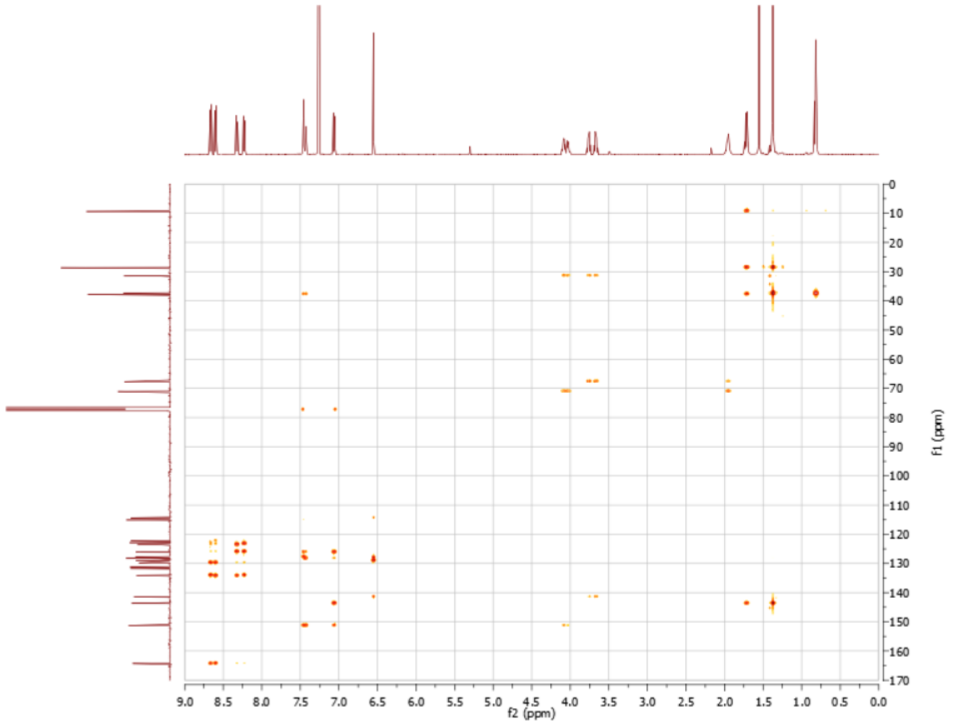

HMBC spectrum of TS-M1.

Through-Space Monomer 2 (TS-M2)

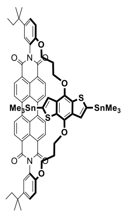

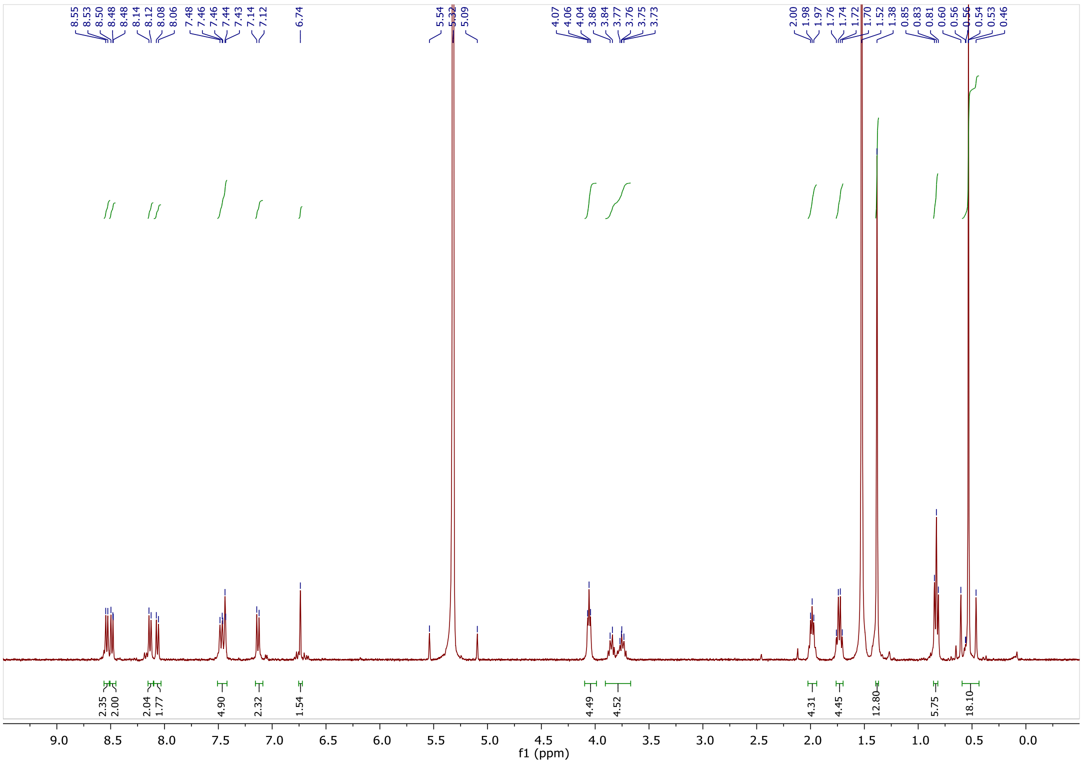

2,5-bis(2-(oxy)propan-1-ol)-3,6-di(thiophen-2-yl)pyrrolo[3,4-c]pyrrole-1,4-dione (DPP-OH)

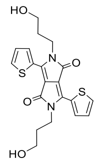

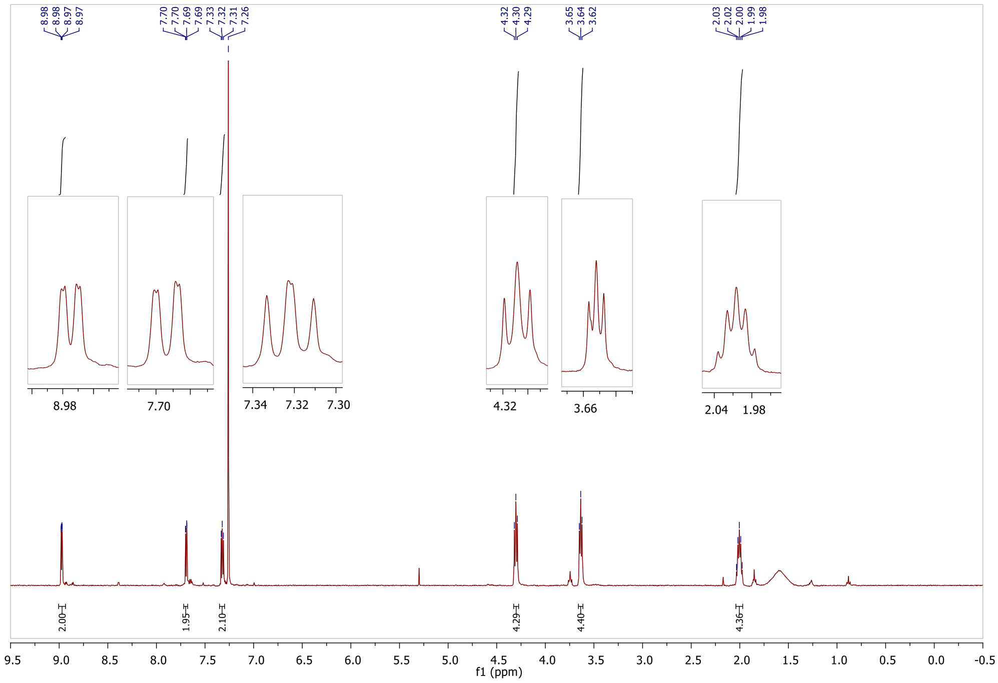

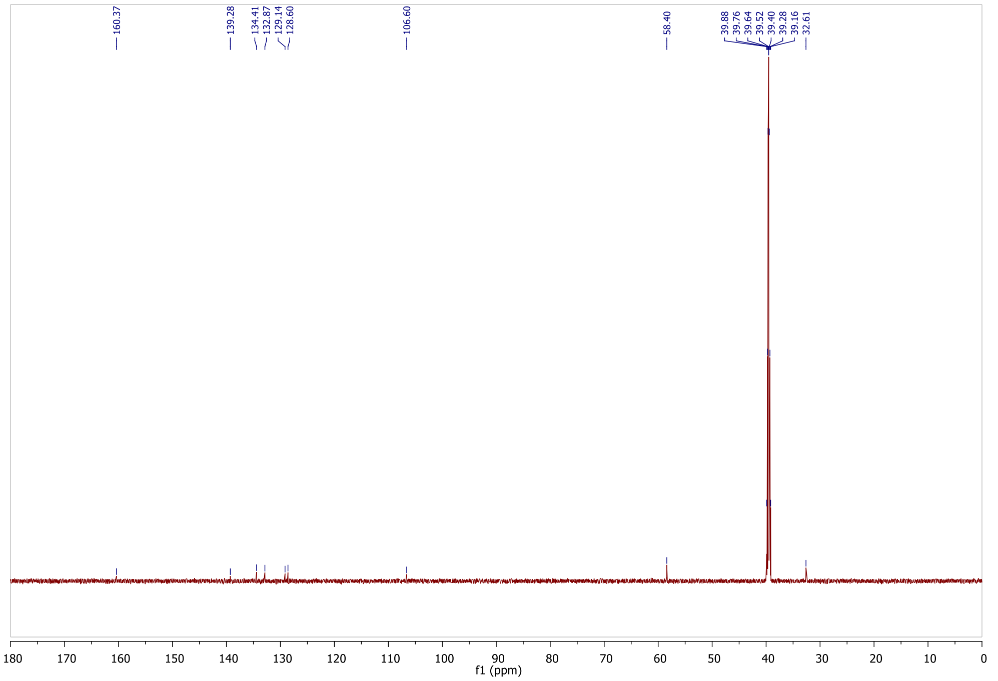

2,5-bis(2-(oxy)propyl(*p*-toluenesulfonate))-3,6-di(thiophen-2-yl)pyrrolo[3,4-c]pyrrole-1,4-dione (DPP-OTs)

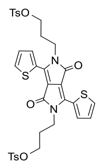
TsO
N
O
S
S
O
N
TsO

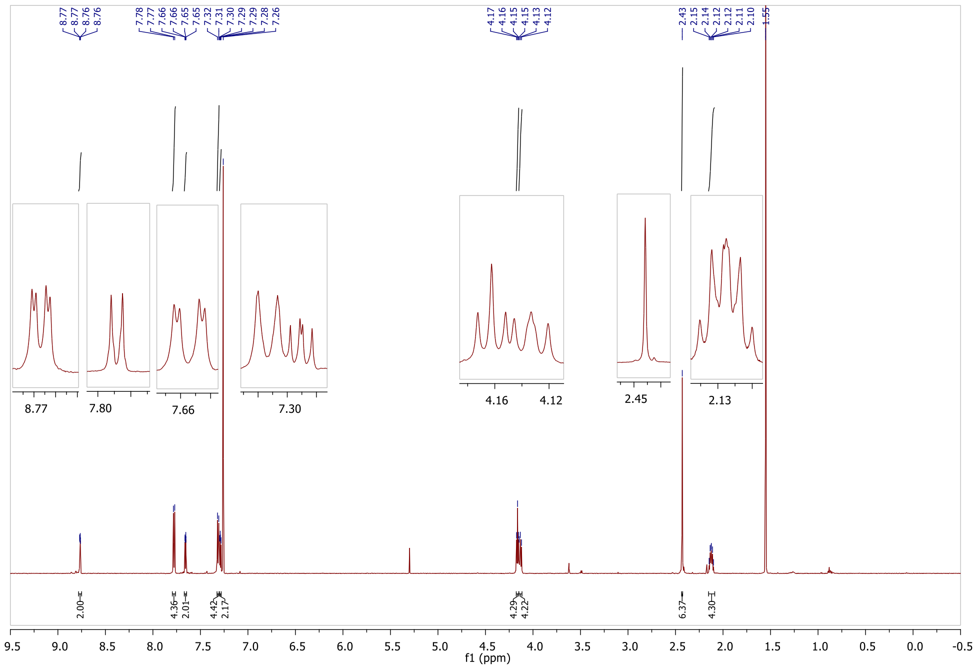
8.77
8.77
8.76
8.76
7.78
7.77
7.66
7.66
7.65
7.65
7.32
7.31
7.30
7.29
7.29
7.28
7.26
4.17
4.16
4.15
4.15
4.13
4.12
2.43
2.15
2.14
2.12
2.12
2.11
2.10
1.55
8.77
7.80
7.66
7.30
4.16
4.12
2.45
2.13
2.00
4.36
2.01
4.42
2.17
4.29
4.22
6.37
4.30
9.5
9.0
8.5
8.0
7.5
7.0
6.5
6.0
5.5
5.0
4.5
4.0
3.5
3.0
2.5
2.0
1.5
1.0
0.5
0.0
-0.5
f1 (ppm)

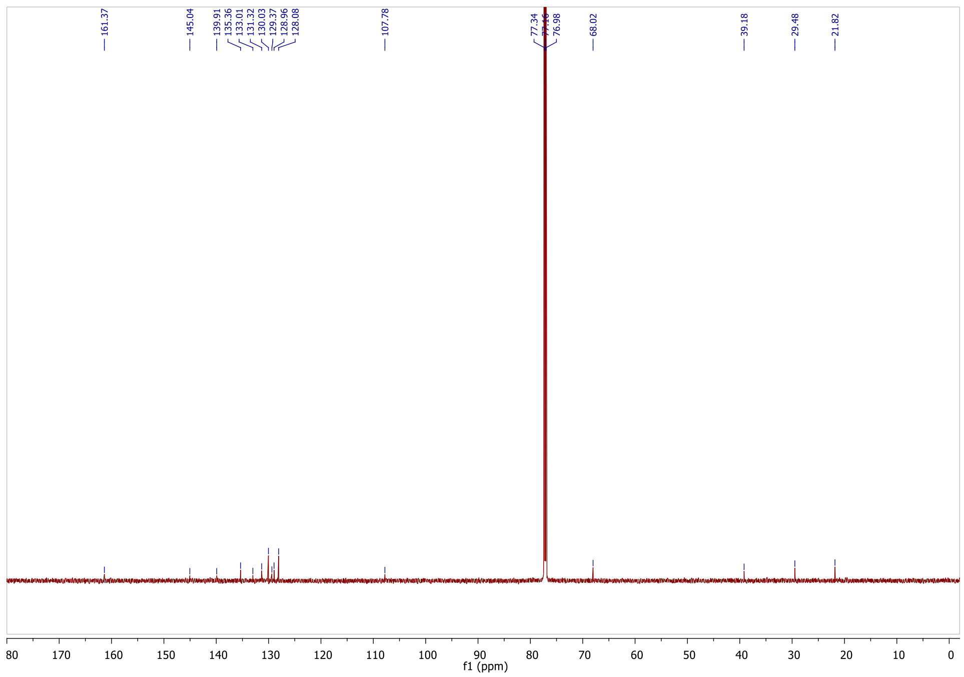
161.37
145.04
139.91
135.36
133.01
131.32
130.03
129.37
128.96
128.08
107.78
77.34
76.98
68.02
39.18
29.48
21.82
180
170
160
150
140
130
120
110
100
90
80
70
60
50
40
30
20
10
0
f1 (ppm)

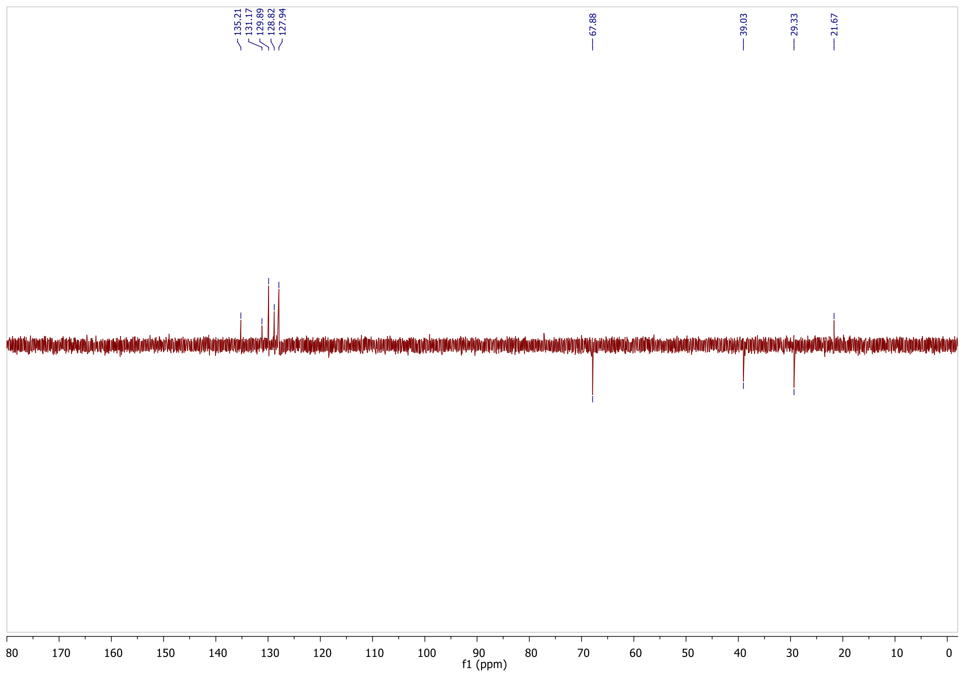

^{13}C DEPT spectrum of DPP-OTs.

TS-PDI-DPP

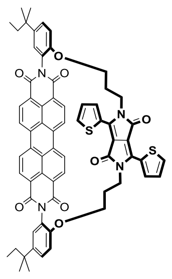

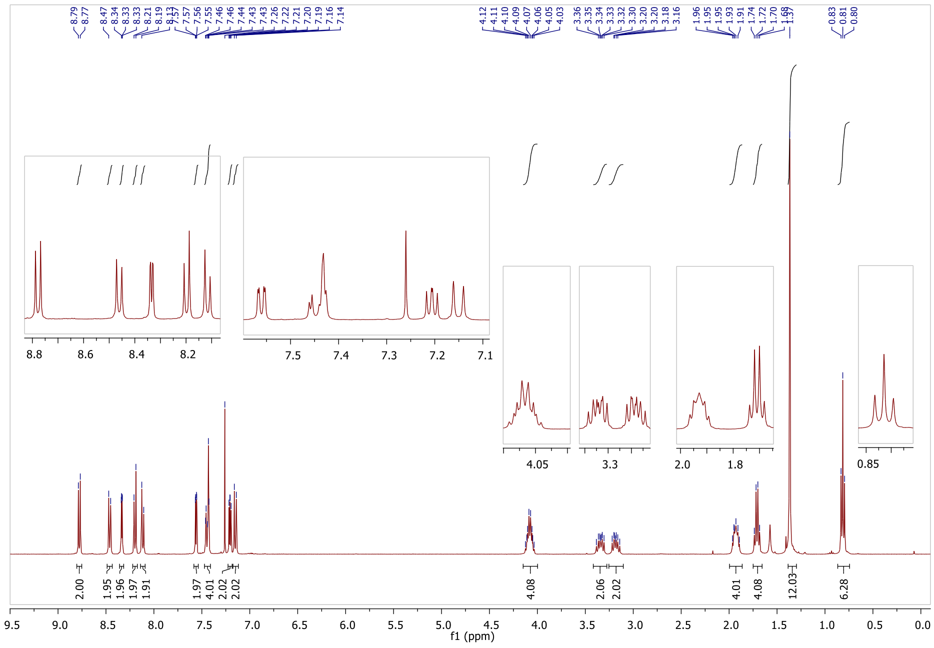

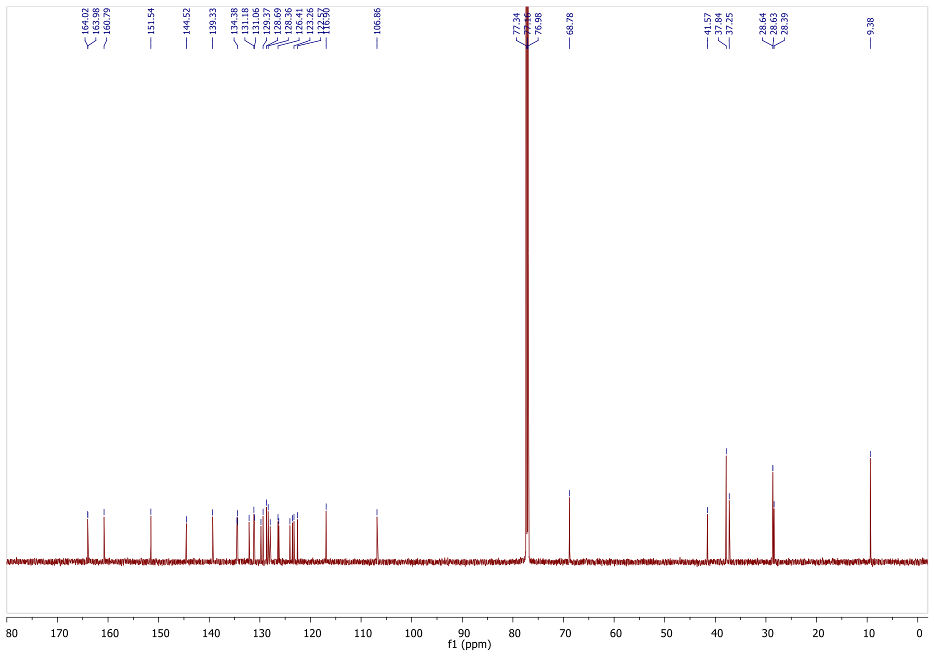

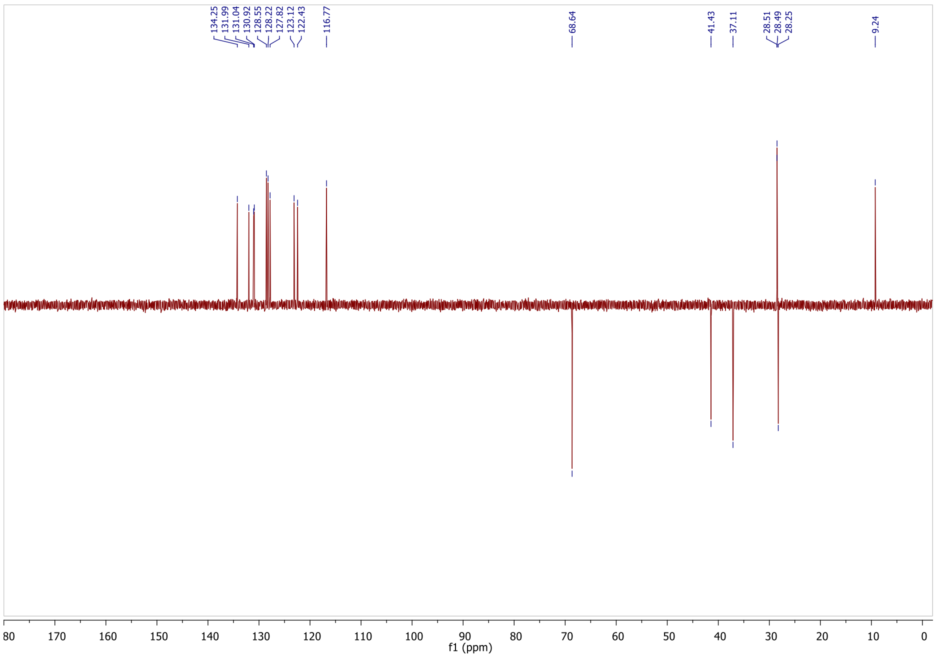

^{13}C DEPT spectrum of TS-PDI-DPP.

Through-Space monomer 3 (TS-M3)

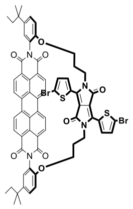

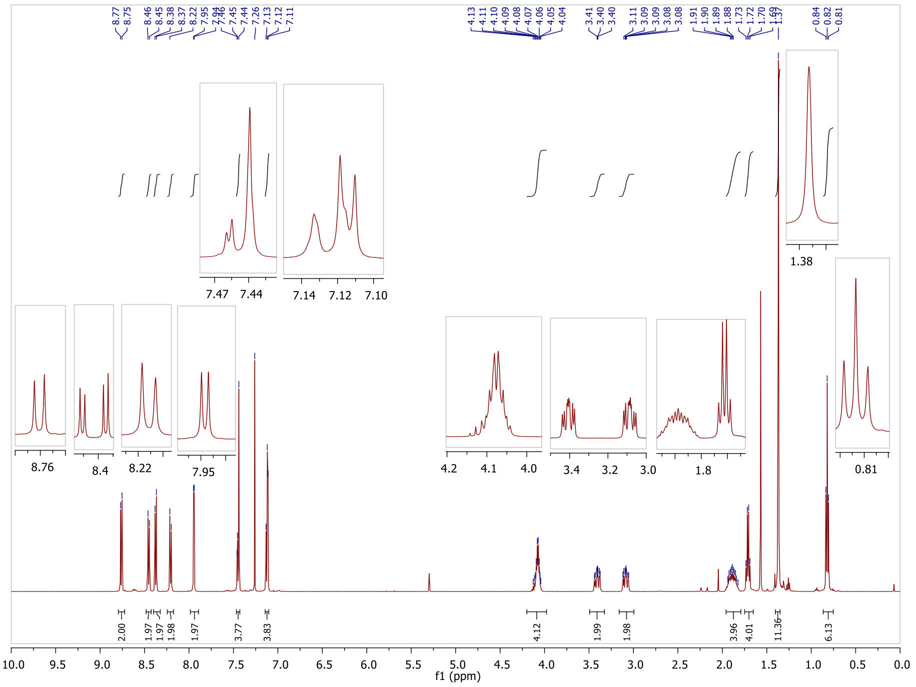

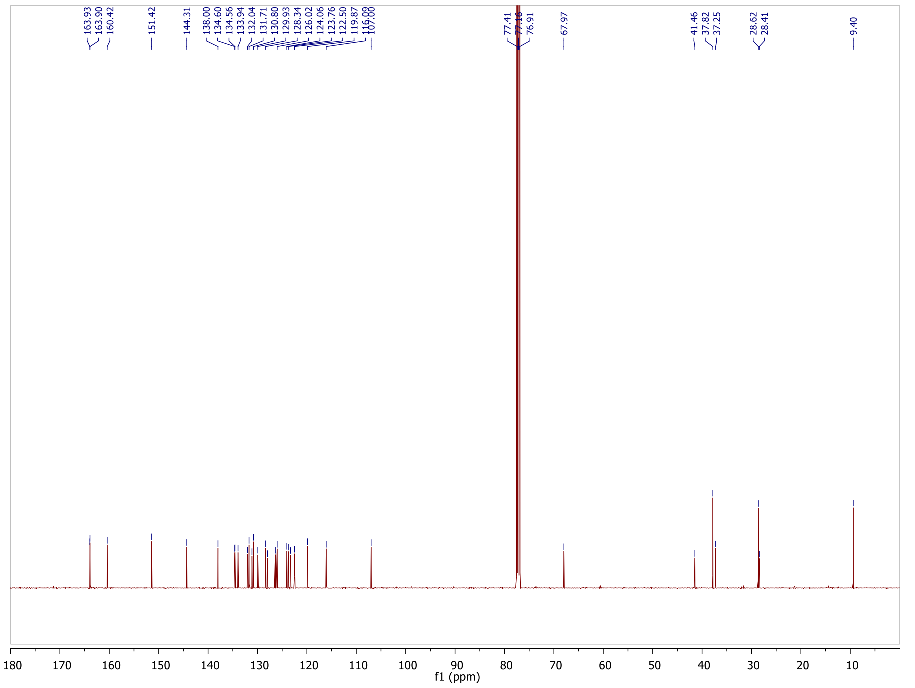

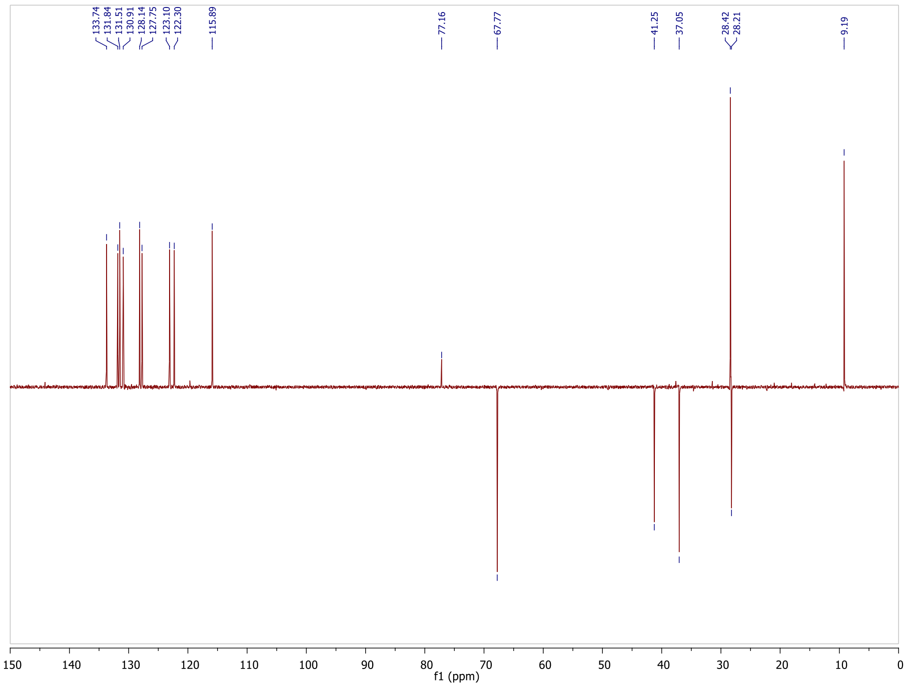

^{13}C DEPT spectrum of TS-M3.

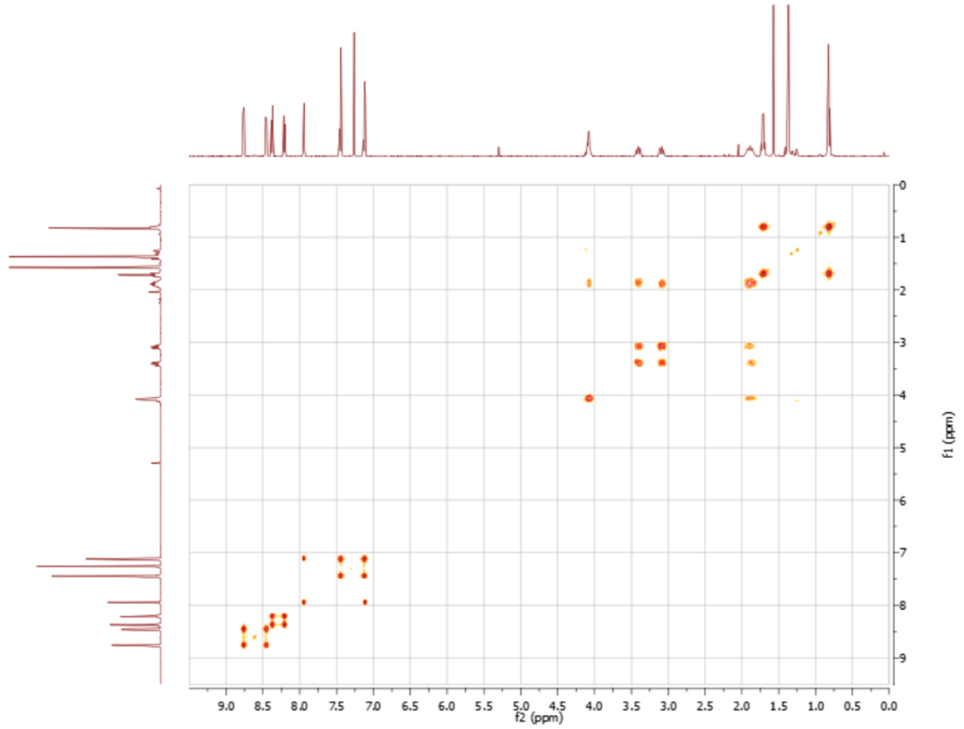

^{1}H-^{1}H COSY spectrum of TS-M3.

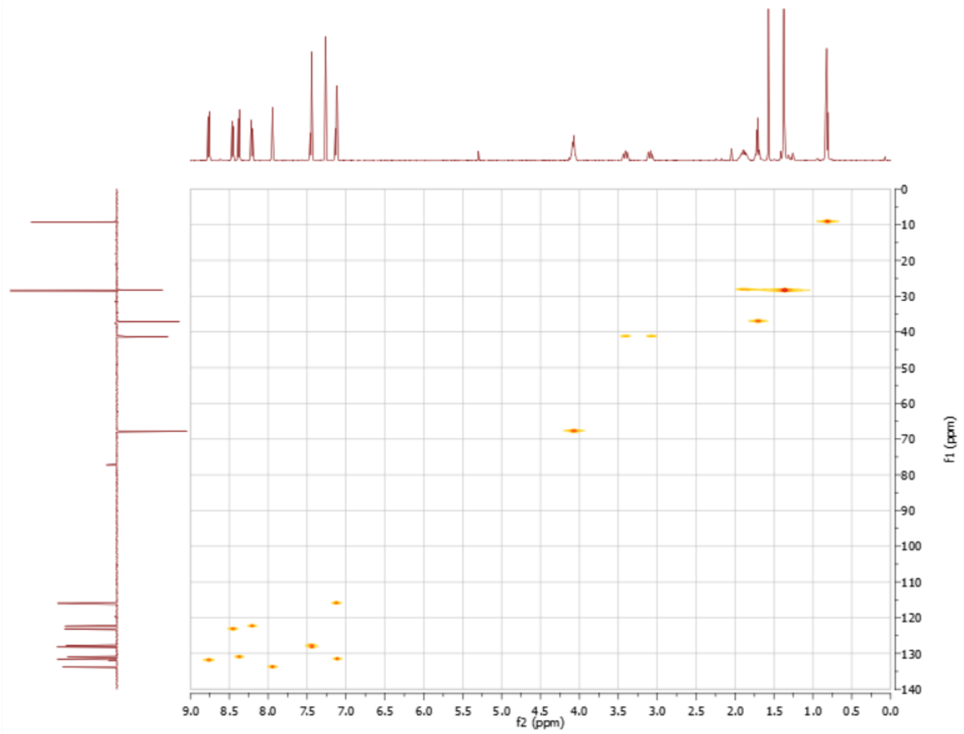

HSQC spectrum of TS-M3, with 13C DEPT spectrum on y-axis.

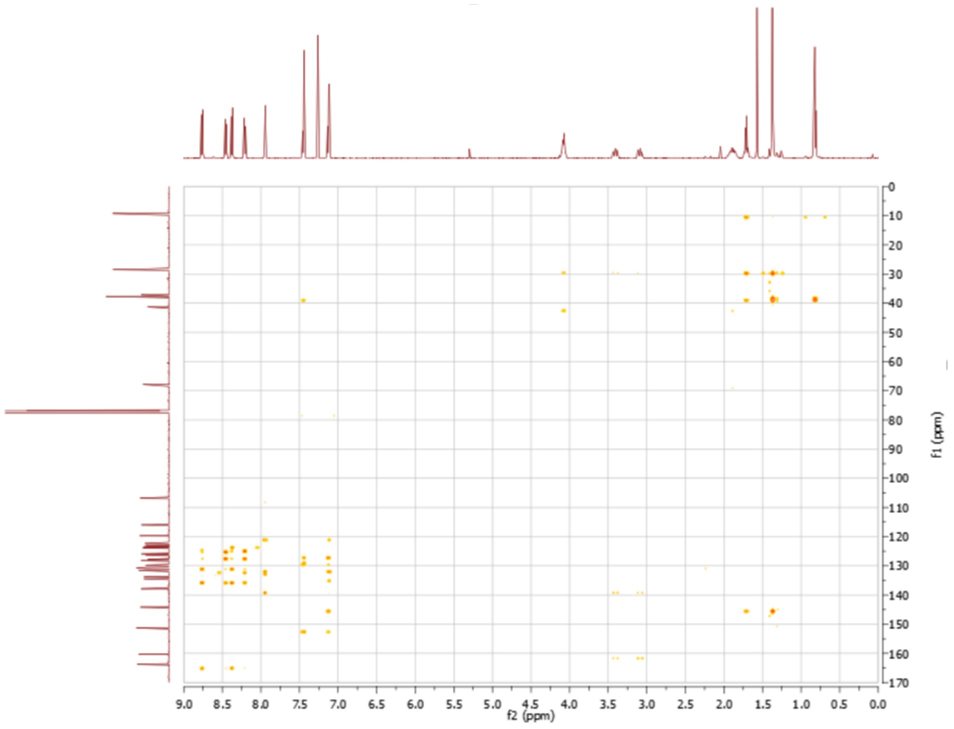

HMBC spectrum of TS-M3.

3. Additional optical measurements

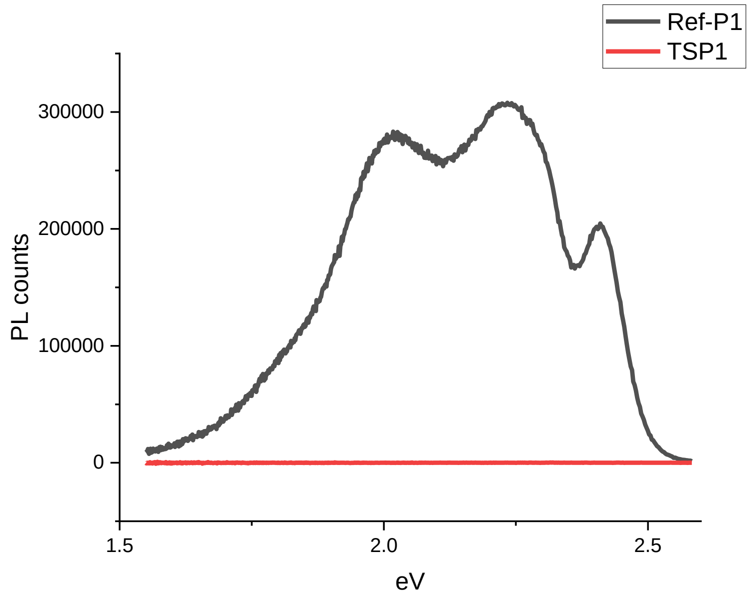

Figure 1. Solution ($CHCl_3$) photoluminescence spectra of Ref-P1 and TSP1

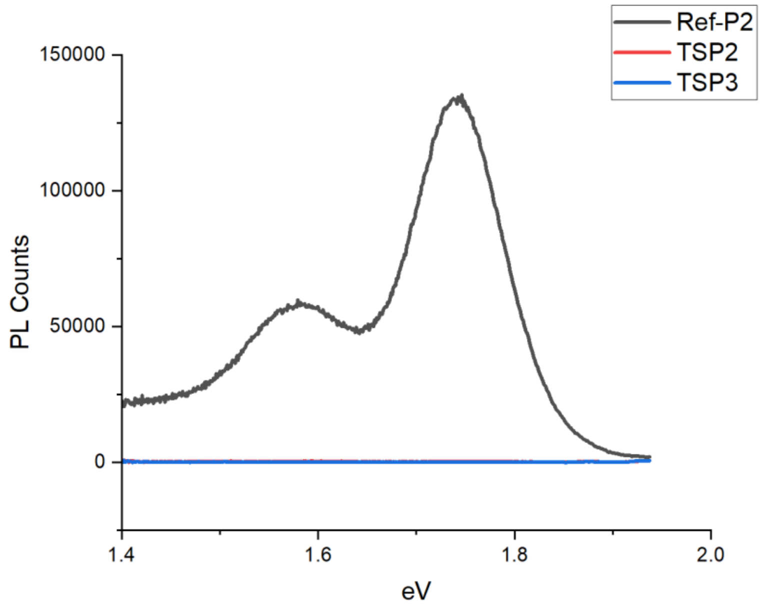

Figure 2. Solution ($CHCl_3$) photoluminescence spectra of Ref-P2 and TSP2 and TSP3

Compound	PLQY (%)
Ref–P1[a]	27
Ref–P2[b]	4
TSP1[a]	-
TSP2[b]	-
TSP3[b]	-

[a]exc 460 nm, [b]exc 630 nm. Measured in $CHCl_3$. No emission was observed for TSP1, TSP2 and TSP3

Table 1. Solution photoluminescence quantum yields of all polymers determined with an integrating spehere

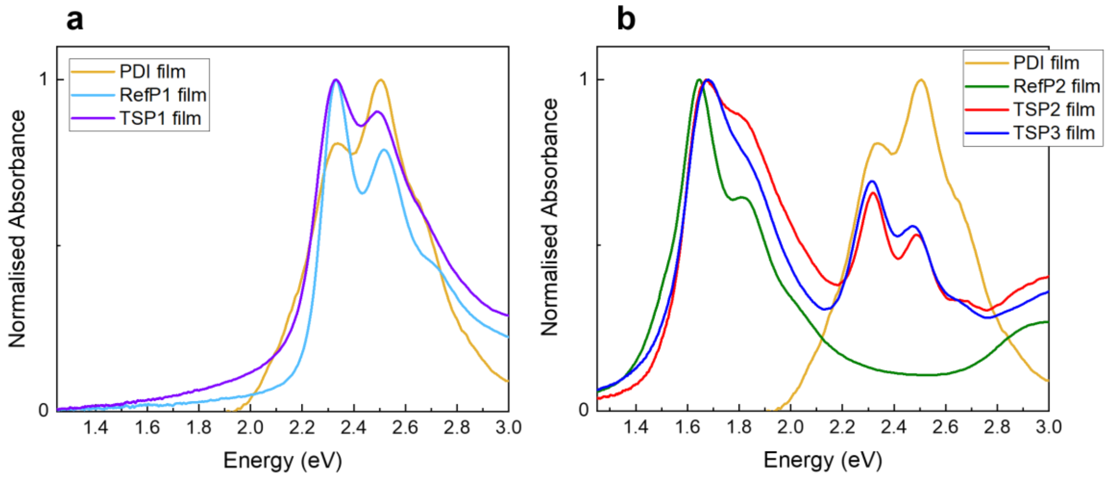

Figure 3. Normalised absorbance of (a) PDI, Ref-P1, TSP1 films and (b) PDI, Ref-P2, TSP2 and TSP3 films.

Despite essentially having the same polymer backbone, TSP2 and TSP3 are clearly spectrally different and the difference in UV-Vis spectra may therefore be a result of the structural differences (i.e. whether the PDI is attached to the DPP or BDT unit). To clarify whether these differences are intramolecular or intermolecular in nature, we measured concentration dependent absorption spectra (Figure S4), which showed little concentration-dependent spectral change. This suggests the differences in spectral shape between TSP2 and TSP3 are likely caused by intramolecular effects. It should also be noted that TSP3 has a substantially higher molecular weight than TSP2, which may affect chain conformation.

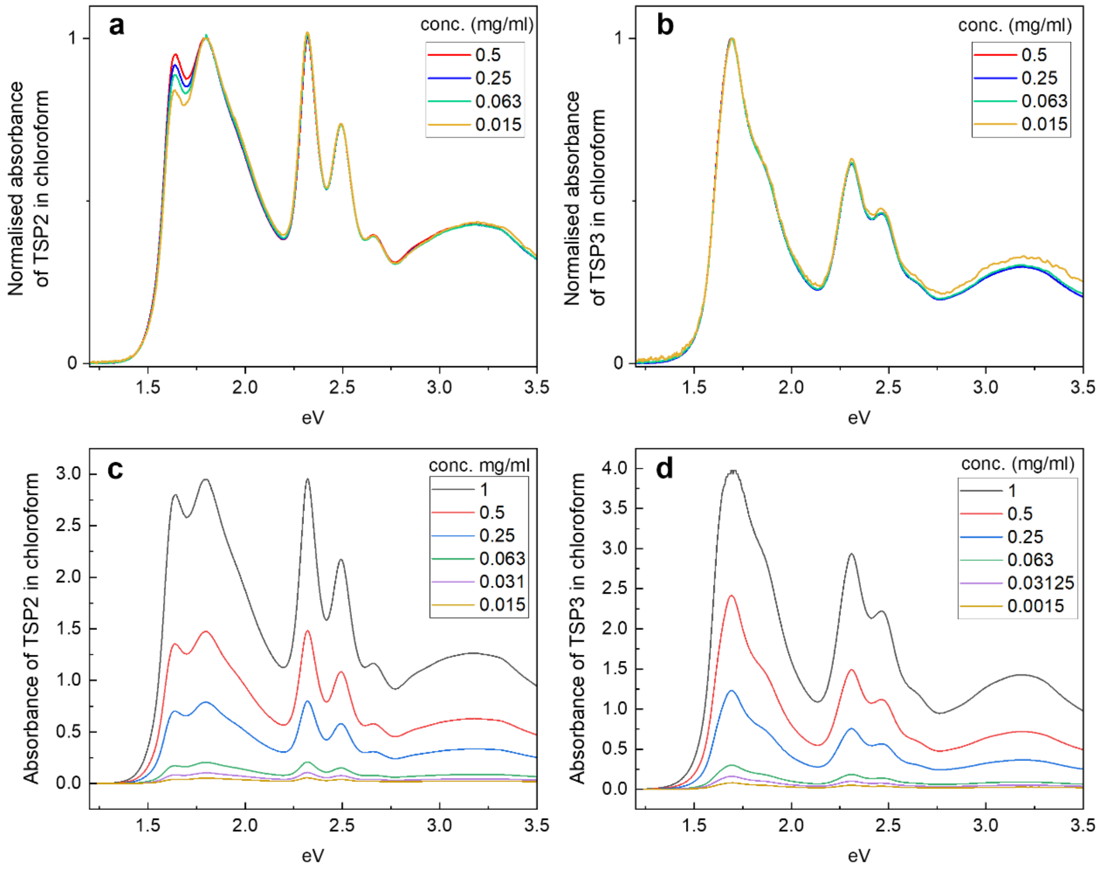

Figure 4. Normalised absorbance of (a) TSP2 and (b) TSP3 in chloroform. The unnormalized absorbance of (c) TSP2 and (d) TSP3 in chloroform are also shown.

4. Comparison of experimental and calculated absorption spectra

The calculated absorption spectra of the ensemble were found to correlate well with the shape of experimental absorption spectra in solution with a stronger shoulder feature also represented in TSP2 (1.84 eV), validating the choice of exchange-correlation functional as being representative of the correct physics. We also note that the differences in intensities of the 0-0 and 0-1 peaks observed in the experimental absorption spectra of TSP2 and TSP3 (Main text Figure 2b) are qualitatively reproduced in the excited-state absorption spectra calculated from the thermal ensemble of conformers, which includes some vibronic effects (Supplementary Fig. 5 and 6), supporting the proposal that the observed spectral differences are intramolecular in nature.

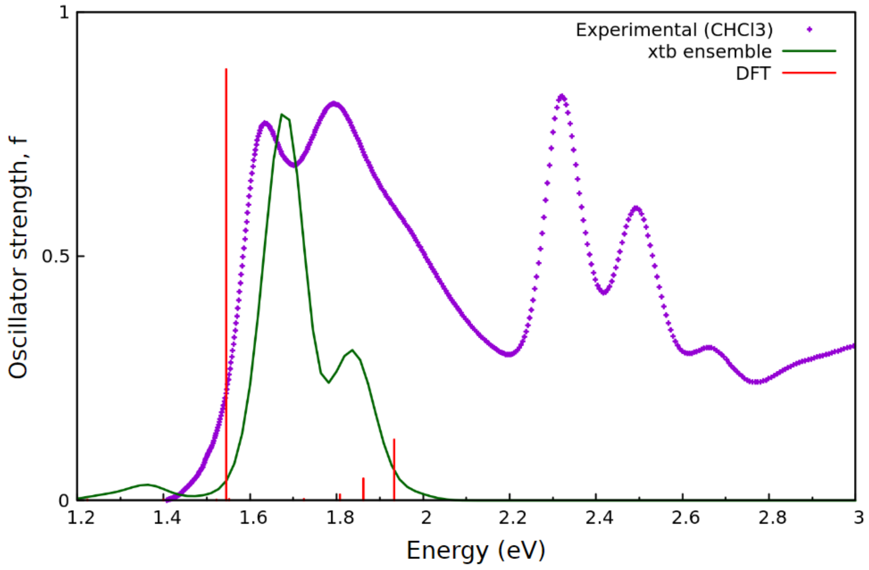

Figure 5. Simulated absorption spectrum of TSP2 obtained from TDDFT calculations on the conformations extracted from the thermal xtb ensemble (plotted with kdensity bandwidth=0.025) (green) in comparison with experimental data (in chloroform solution) (purple). The TDDFT excited state spectrum for the geometry optimised trimer is also shown (red). Note that the xtb and TDDFT spectra stop at 2eV since excited states were only calculated up to this energy.

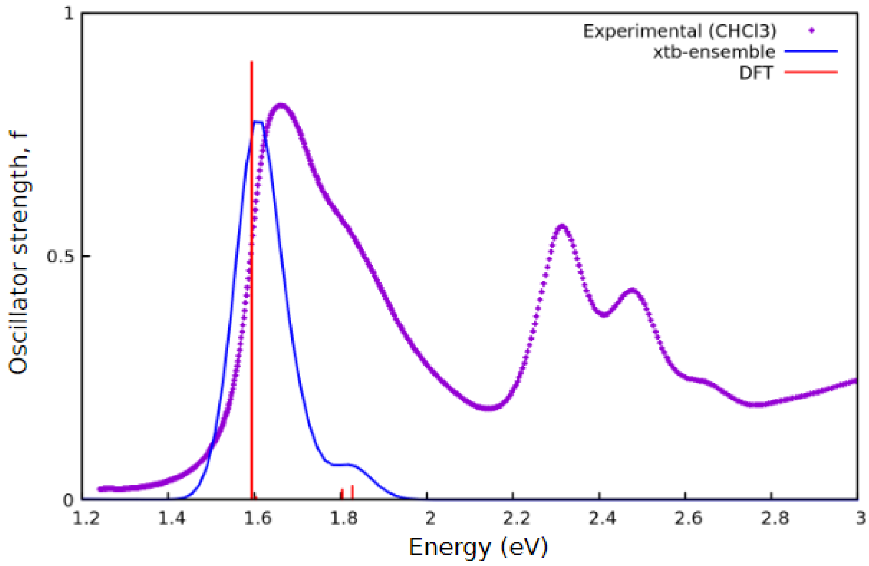

Figure 6. Simulated absorption spectrum of TSP3 obtained from TDDFT calculations on the conformations extracted from the thermal xtb ensemble (plotted with kdensity bandwidth=0.025) (blue) in comparison with experimental data (in chloroform solution) (purple). The TDDFT excited state spectrum for the geometry optimised trimer is also shown (red). Note that the xtb and TDDFT spectra stop at 2 eV since excited states were only calculated for the first 20 singlet states.

5. Analysis of D:A orientations.

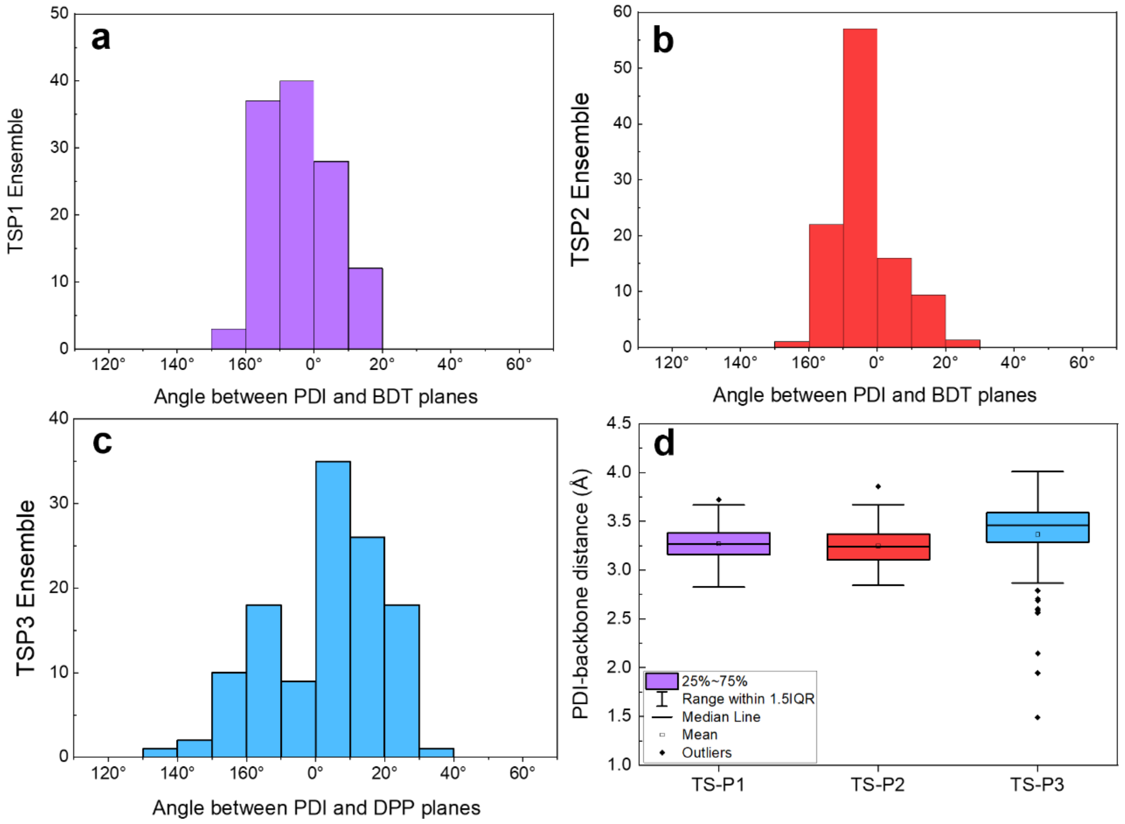

Figure 7. Histograms showing all counts (n=120) of relative orientations between PDI and its bonded (a) BDT plane in the TSP1 trimer ensemble, (b) BDT plane in the TSP2 trimer ensemble and (c) DPP plane in the TSP3 trimer. The relative orientations are calculated from the dot product of the molecular planes' normal vectors. The statistics of the distances between the two molecules are summarised in (d) (n=120 for each TS molecule).

6. Thin-film photo and electroluminescence

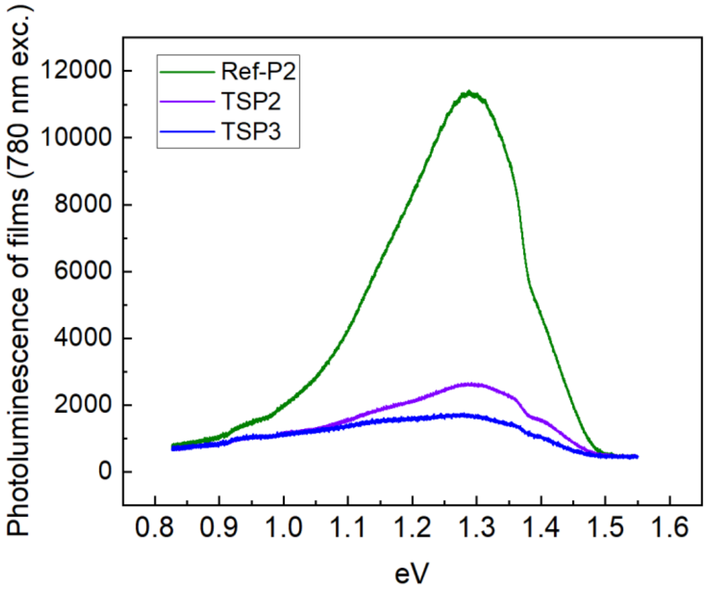

Figure 8. Thin film photoluminescence spectra of Ref-P2 and TSP2 and TSP3

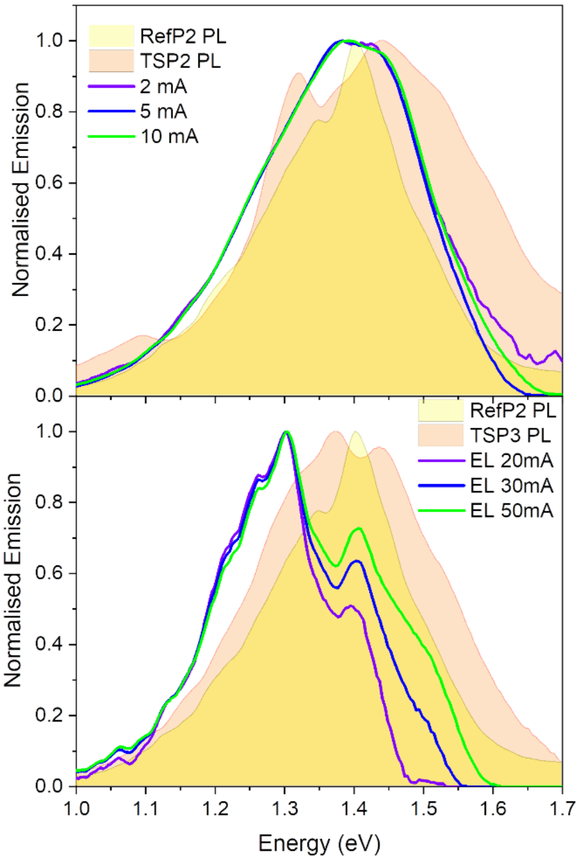

Figure 9. Normalized electroluminescence and photoluminescence data of TSP2 (top) and TSP3 (bottom) at different injection currents.

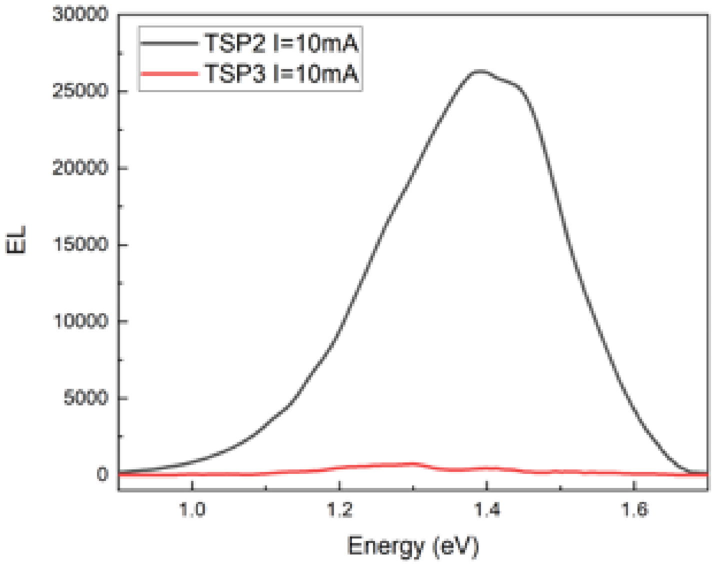

Figure 10. Absolute electroluminescence counts of TSP2 and TSP3 devices at 10 mA injection current.

7. Excited state analysis

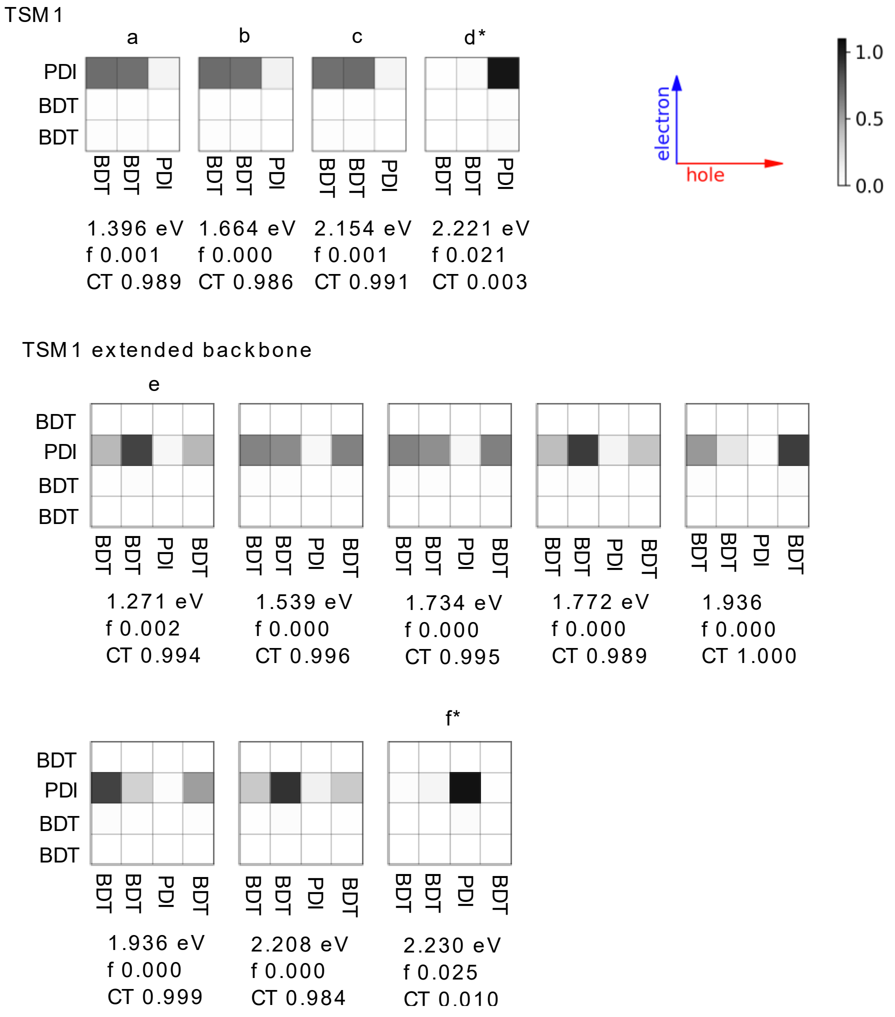

**the BDT label in TSM1 extended backbone represents two BDT units.*

Figure 11. Correlation-plots showing full electron-hole distribution in TSM1 and TSM1 with an extended backbone*. The greyscale shows the strength of charge transfer character and is represented in square-root. The energy of the states, as well as their respective oscillator strengths, f and charge transfer characters, CT are shown below each box.

TSP1 trimer

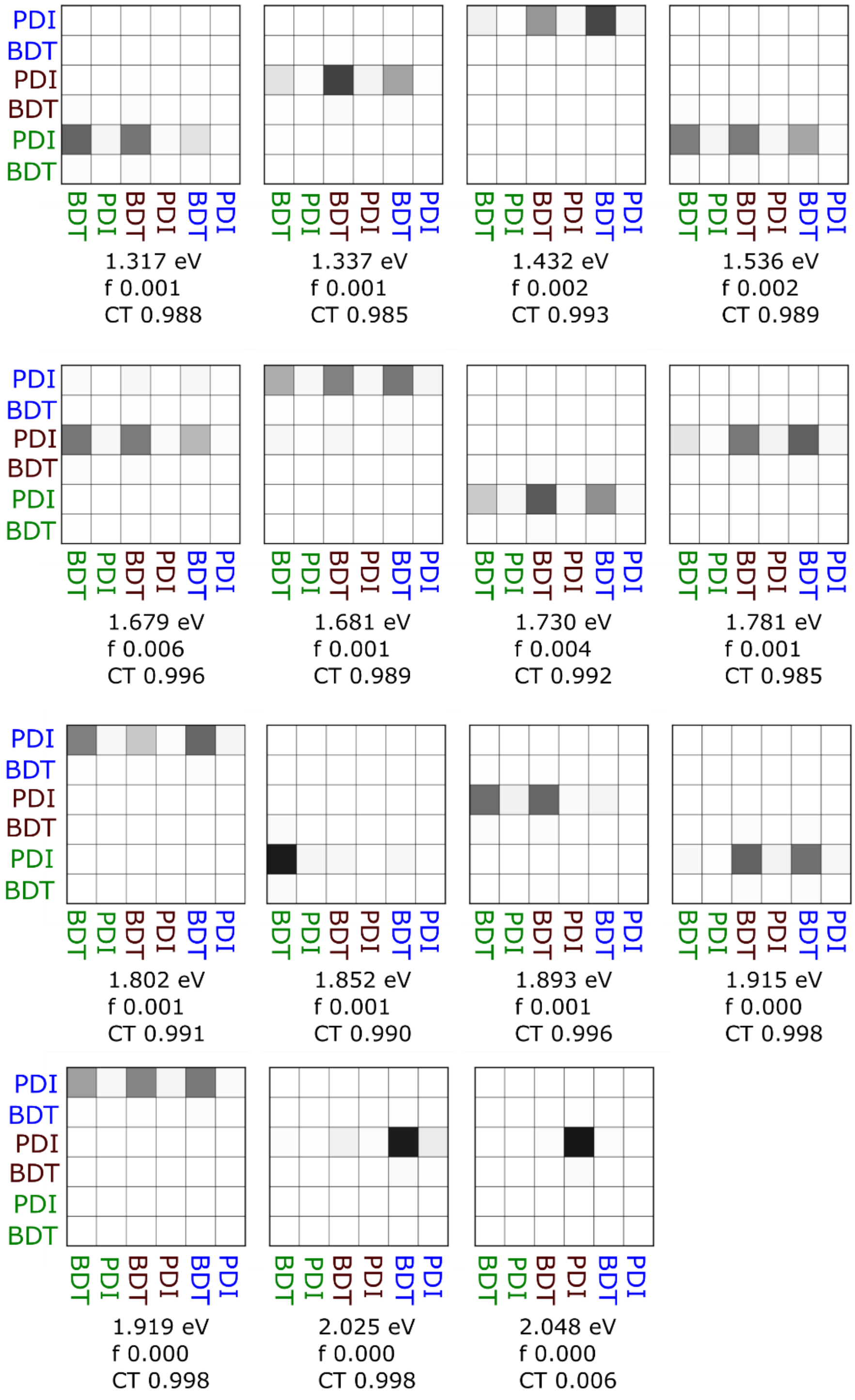
PDI
BDT
PDI
BDT
PDI
BDT
1.317 eV
f 0.001
CT 0.988
1.337 eV
f 0.001
CT 0.985
1.432 eV
f 0.002
CT 0.993
1.536 eV
f 0.002
CT 0.989
1.679 eV
f 0.006
CT 0.996
1.681 eV
f 0.001
CT 0.989
1.730 eV
f 0.004
CT 0.992
1.781 eV
f 0.001
CT 0.985
1.802 eV
f 0.001
CT 0.991
1.852 eV
f 0.001
CT 0.990
1.893 eV
f 0.001
CT 0.996
1.915 eV
f 0.000
CT 0.998
1.919 eV
f 0.000
CT 0.998
2.025 eV
f 0.000
CT 0.998
2.048 eV
f 0.000
CT 0.006

TSP1 trimer cont.

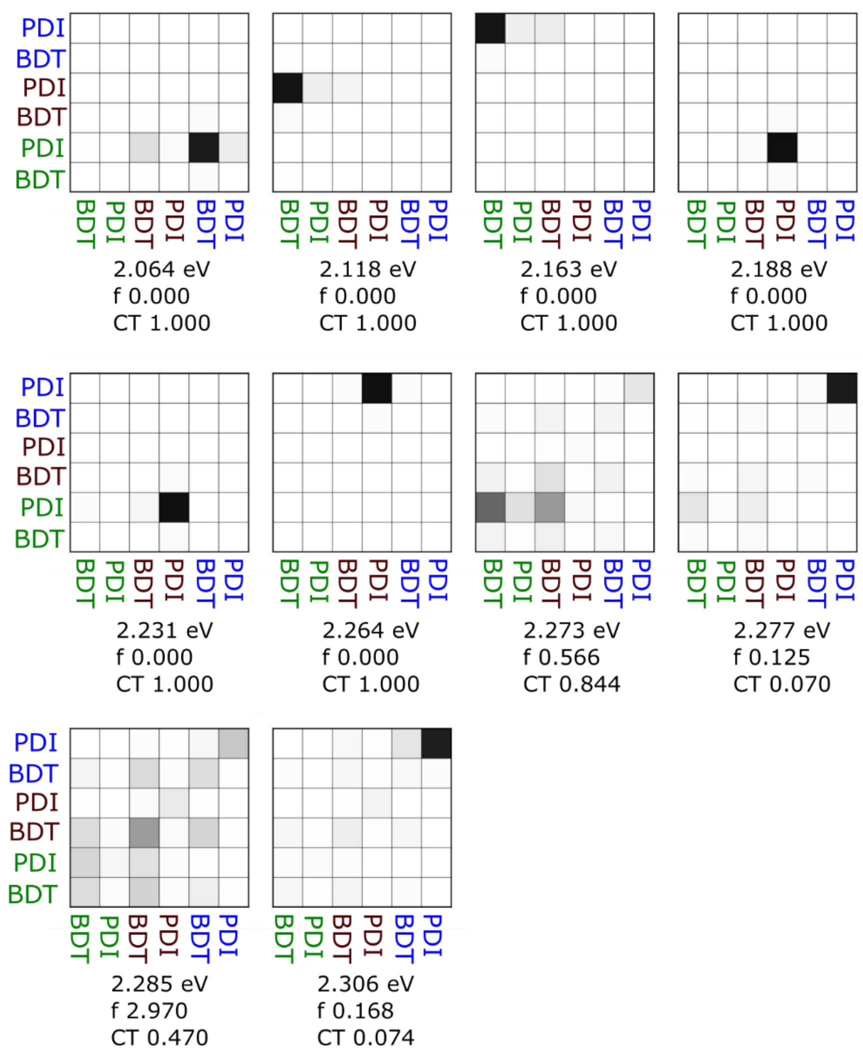

Figure 12. Correlation-plots showing electron-hole distribution in TSP1 trimer (each BDT label represents two BDT units). The greyscale shows the strength of charge transfer character and is represented in square-root.

TSP2 trimer

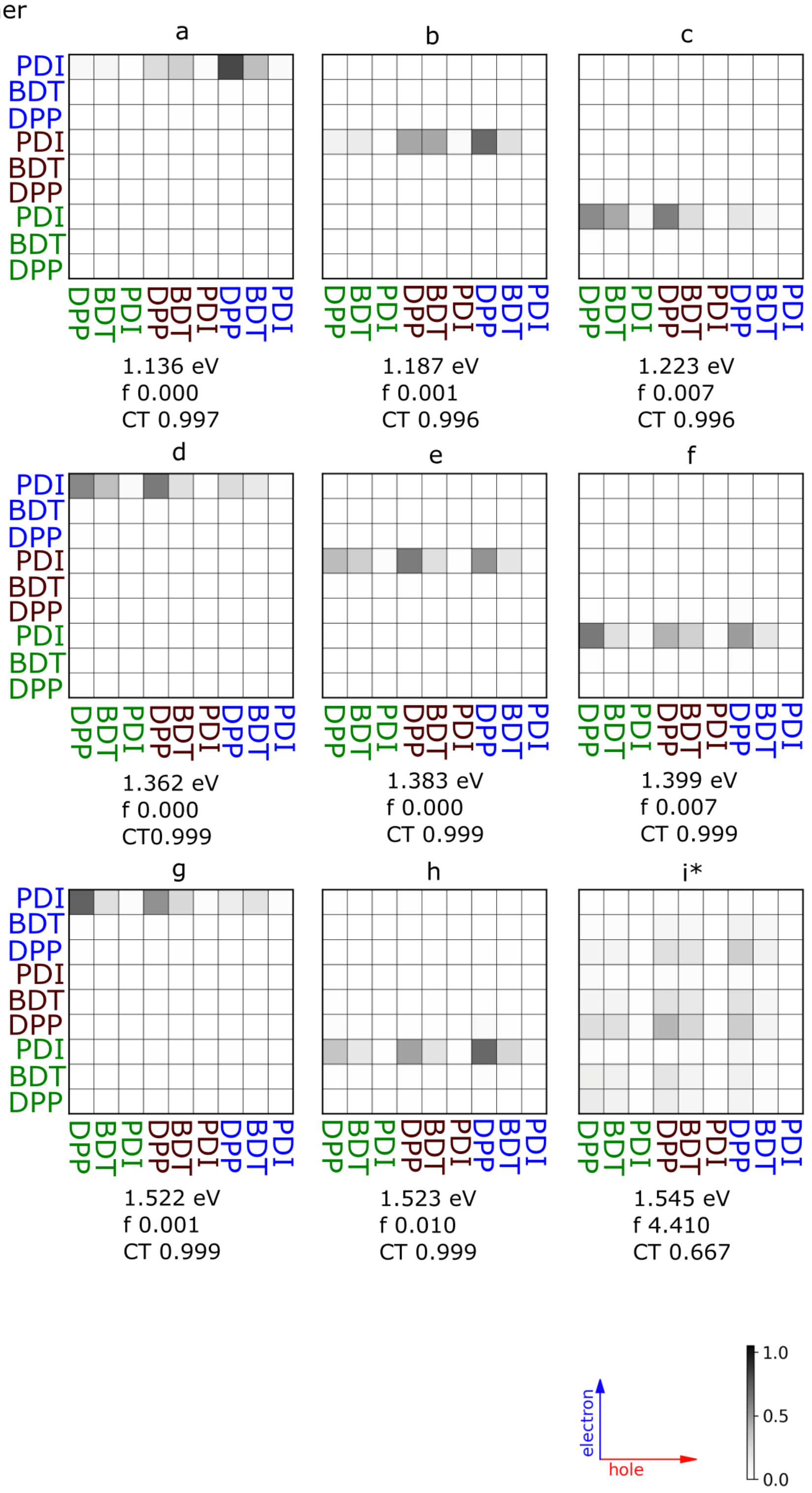

Figure 13. Correlation-plots showing electron-hole distribution in TSP2 trimer. The tricolours represent the three fragments on the trimer. The greyscale shows the strength of charge transfer character and is represented in square-root.

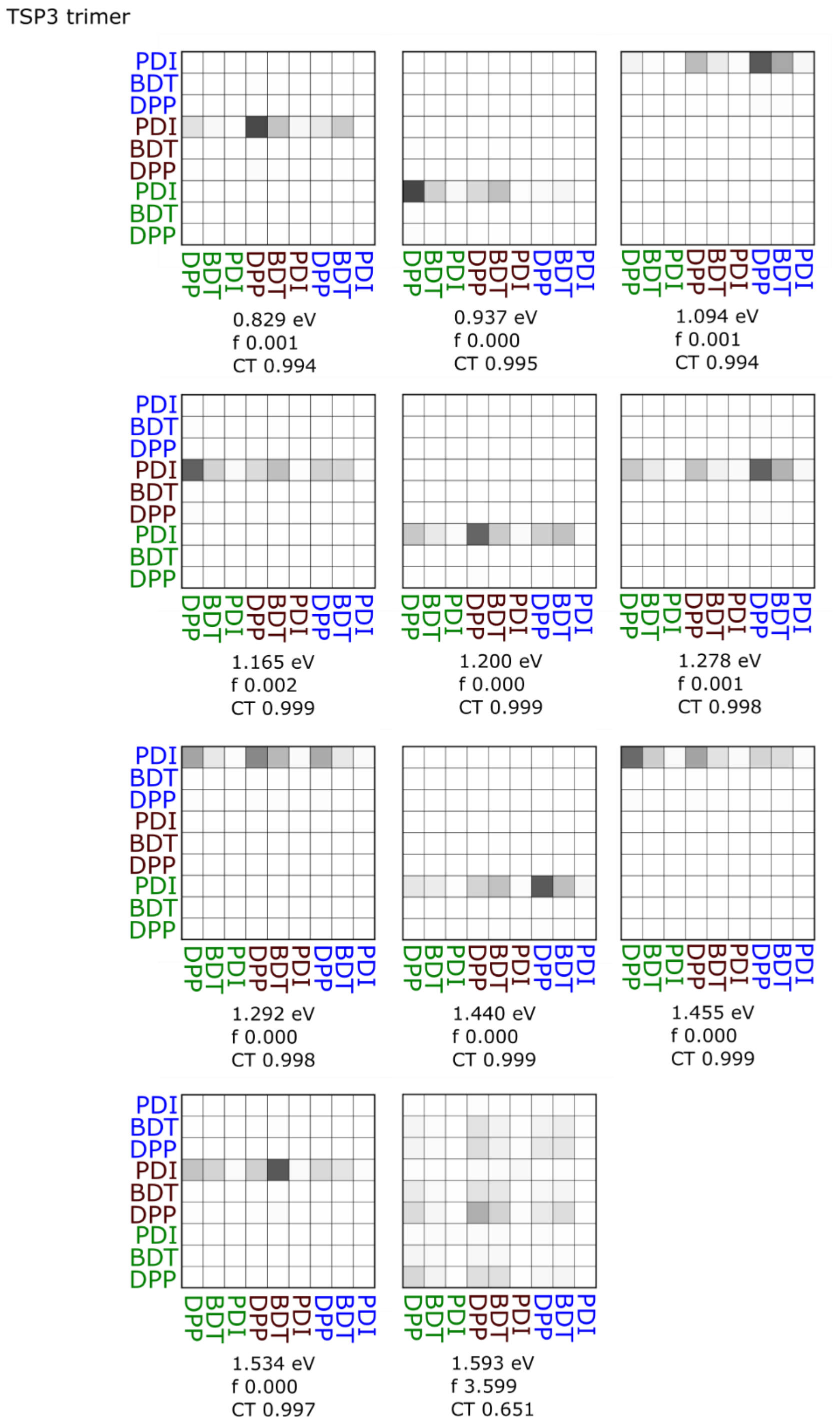

Figure 14. Correlation-plots showing electron-hole distribution in TSP3 trimer. The tricolours represent the three fragments on the trimer. The greyscale shows the strength of charge transfer character and is represented in square-root.

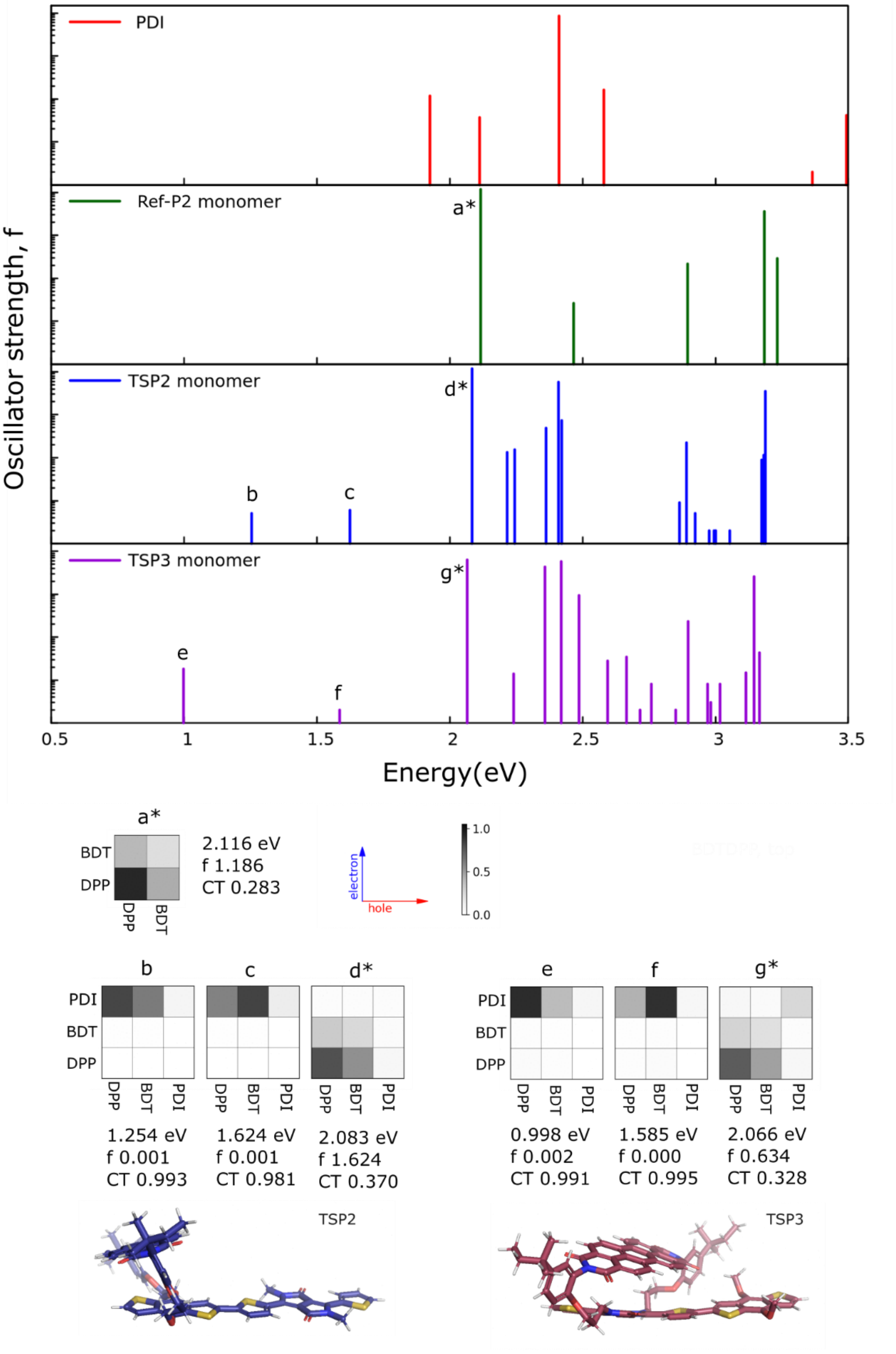

Figure 15. Excited state calculation using time-dependent density functional theory (TDDFT, B3LYP/6-31g*) on the geometry optimised structures of PDI, Ref-P2 monomer, TSP2 monomer and TSP3 monomer. Their first excitonic state and charge transfer state are shown in correlation-plots as well as their energies, oscillator strengths, f and charge transfer characters, CT. The bright excitonic states are labelled with an Asterix (*).

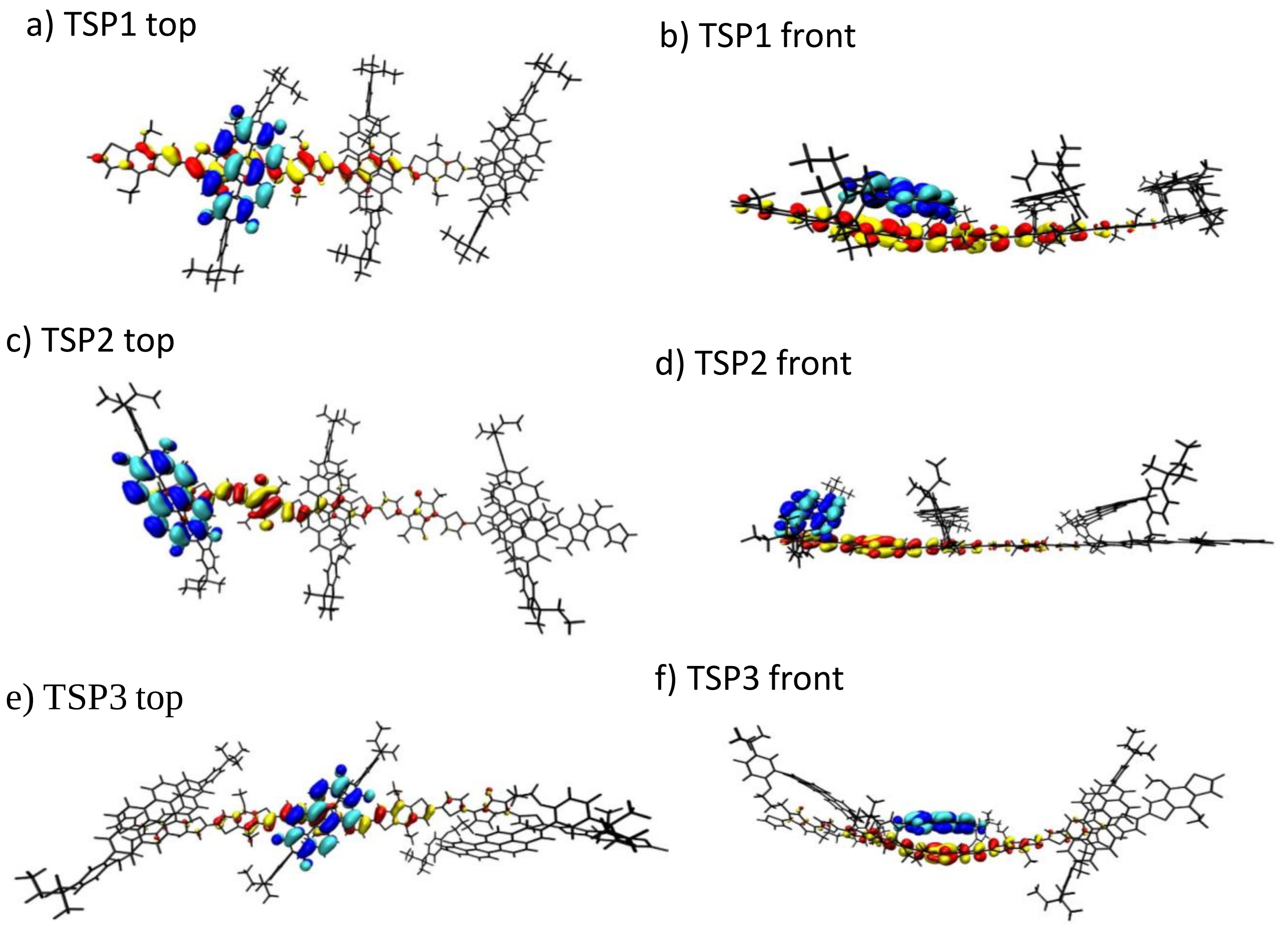

Figure 16. Natural transition orbitals of the first excited singlet of TSP1 (a, b), TSP2 (c, d) and TSP3 (e, f). Isovalues for the electron (blue and cyan) and hole (red and yellow) are +/- 0.02 electrons per Bohr^3.

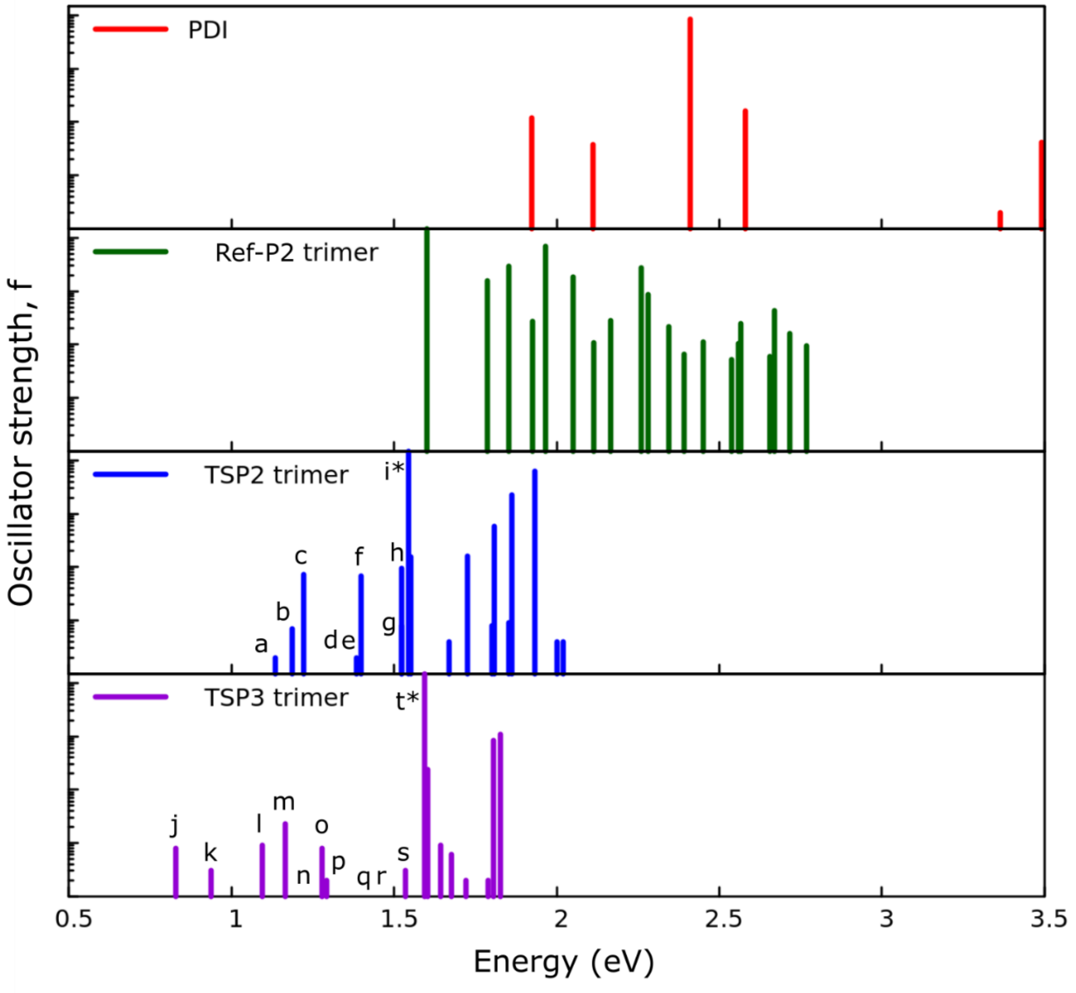

Figure 17. Simulated absorption spectra of Ref-P2 trimer, TSP2 and TSP3 trimers. The charge transfer states are labelled with letters and each lowest excitonic state is specified with an Asterix (*).

TSP2			TSP3		
States (eV)	Oscillator strength	Participation Ratio	States (eV)	Oscillator strength	Participation Ratio
1.136	0	1.504	0.829	0.001	1.513
1.187	0.001	2.031	0.937	0	1.49
1.223	0.007	2.234	1.094	0.001	1.716
1.362	0	2.235	1.165	0.002	1.988
1.383	0	2.414	1.2	0	2.073
1.399	0.007	2.399	1.278	0.001	1.941
1.522	0.001	1.853	1.292	0	2.642
1.523	0.01	2.065	1.44	0	1.816
1.545	4.41	4	1.455	0	2.121
1.551	0.015	1.548	1.534	0	1.789
1.67	0	1.362	1.593	3.599	4.012
1.725	0.016	3.29	1.604	0.024	2.518
1.8	0.001	1.667	1.642	0.001	1.671
1.808	0.058	4.136	1.676	0.001	1.976
1.853	0.001	1.559	1.722	0	1.731
1.862	0.226	4.053	1.789	0	1.294
1.934	0.623	3.677	1.805	0.085	3.479
1.971	0	1.716	1.828	0.111	3.808
2.002	0	1.005	1.878	0	1.998
2.021	0	1.006	1.879	0	1.498

Table 2. TSP2 and TSP3's oscillator strengths and participation ratio (PR) for the first 20 singlet states.

8. Additional transient absorption measurements

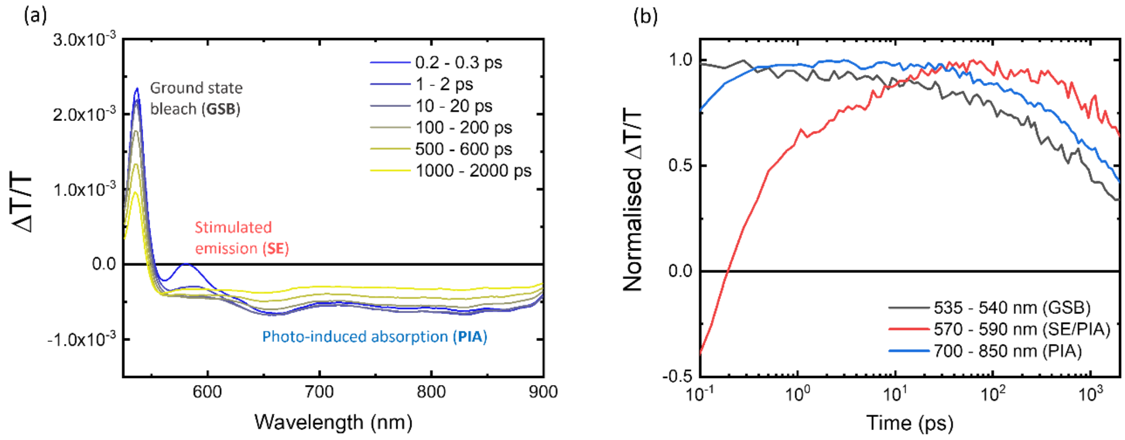

Figure 18. TAS spectra (a) and extracted kinetics (b) of Ref-P1-reference polymer, measured 0.1mg/ml in toluene with a 490nm pulse at a fluence of 0.98 $\mu J/cm^{-2}$.

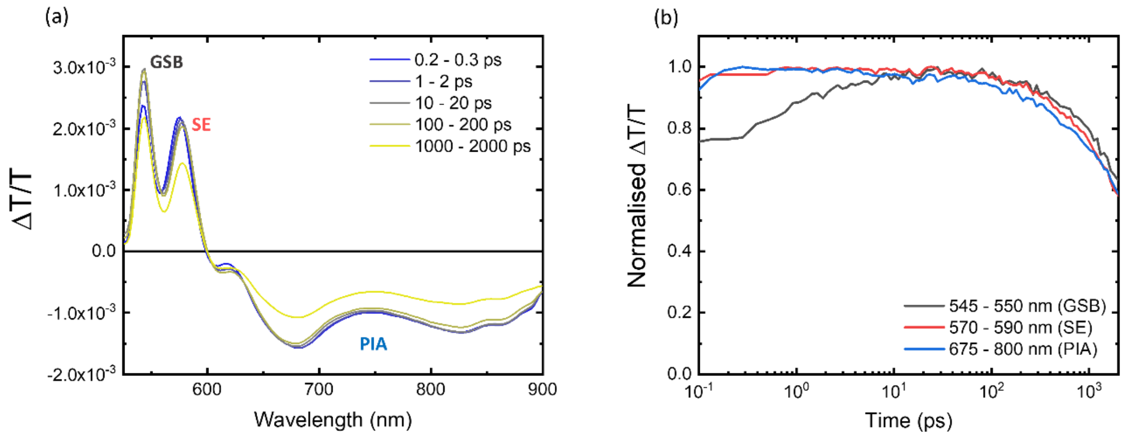

Figure 19. TAS spectra (a) and extracted kinetics (b) of PDI, measured 0.1mg/ml in toluene with a 490nm pulse at a fluence of 3.75 $\mu J/cm^{-2}$.

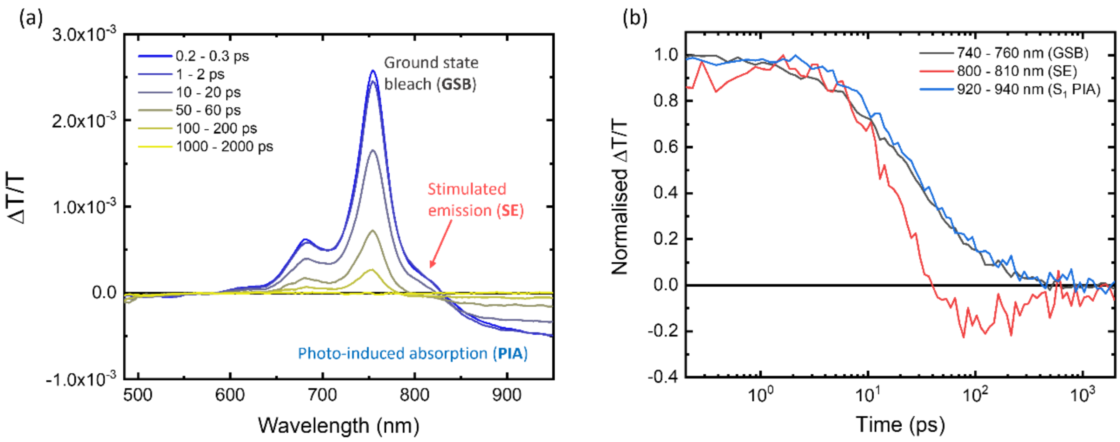

Figure 20. TAS spectra (a) and extracted kinetics (b) of Ref-P2 reference polymer, measured 0.1mg/ml in toluene with a 700 nm pulse at a fluence of 1.41 $\mu J/cm^{-2}$.

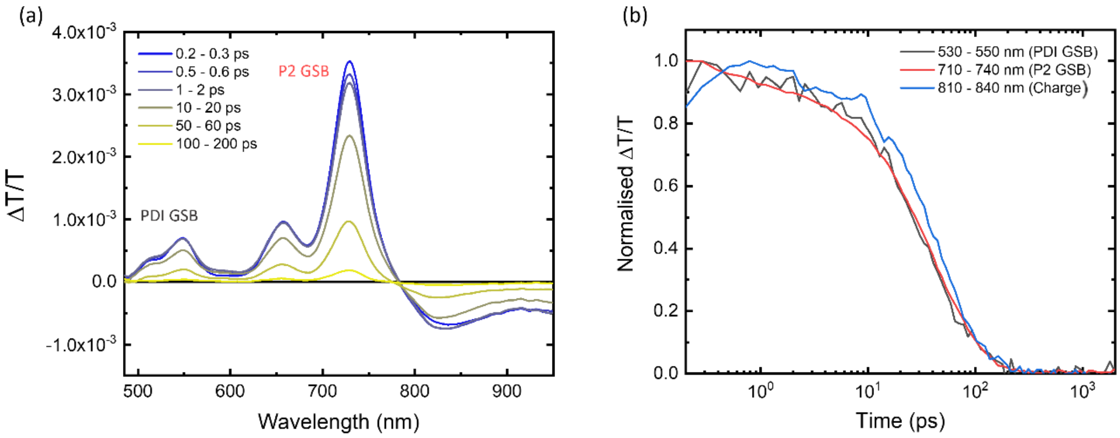

Figure 21. TAS spectra (a) and extracted kinetics (b) of TSP3, measured 0.1mg/ml in toluene with a 495 nm pulse at a fluence of 8.94 $\mu J/cm^{-2}$

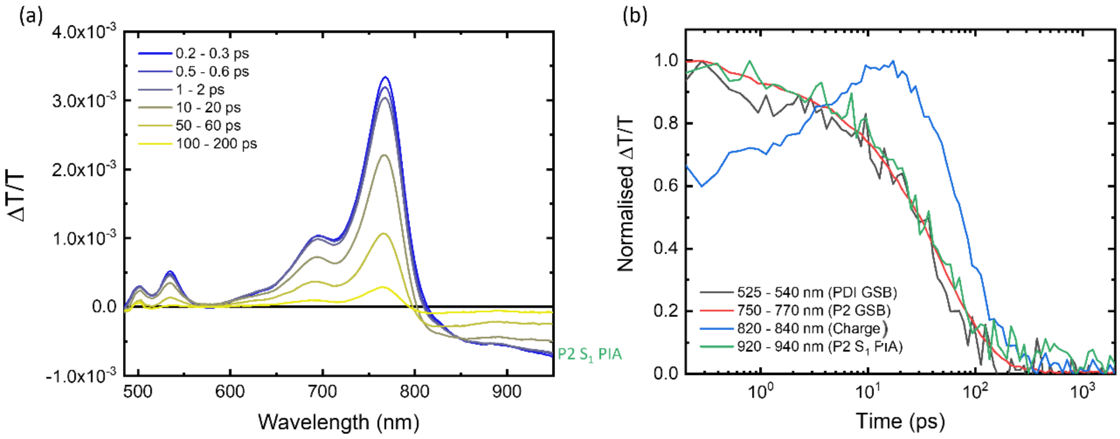

Figure 22. TAS spectra (a) and extracted kinetics (b) of TSP2, measured 0.1mg/ml in toluene with a 495 nm pulse at a fluence of 8.94 $\mu J/cm^{-2}$

9. Effect of geometry relaxation on excited state properties

To probe excited state evolution, we relaxed 1.5-mers of TSP2 and TSP3 in their excited states and found that in both cases the CT state is stabilised by several tenths of an eV while the charge distribution remains unchanged with electron localised on PDI and hole localised on DPP fragments (Supplementary Table 3-4). TSP3 shows a lower relaxed CT state energy and a similar or slightly larger reorganisation energy for the CT to ground transition than TSP2.

TSP3: (BDT-DPP(-PDI-)-BDT Ground state geometry												
state	Energy (eV)	Oscillator strength	Hole Distributions					Electron distribution				
			BDT1	DPP	BDT2	PDI	Other	BDT1	DPP	BDT2	PDI	Other
CT	0.82	0.00	0.11	0.76	0.11	0.00	0.02	0.00	0.00	0.00	1.00	0.00
LE	1.84	1.39	0.17	0.62	0.17	0.03	0.03	0.10	0.77	0.10	0.04	0.01
TSP3: (BDT-DPP(-PDI-)-BDT S1 Geometry												
state	Energy (eV)	Oscillator strength	Hole Distribution					Electron distribution				
			BDT1	DPP	BDT2	PDI	Other	BDT1	DPP	BDT2	PDI	Other
CT	0.29	0.00	0.10	0.78	0.11	0.00	0.01	0.00	0.00	0.00	0.99	0.00
LE	1.70	1.08	0.17	0.60	0.18	0.05	0.02	0.10	0.62	0.10	0.20	0.01
TSP2: (DPP-BDT(-PDI-)-DPP Ground state geometry												
state	Energy (eV)	Oscillator strength	Hole Distribution					Electron distribution				
			DPP1	BDT	DPP2	PDI	Other	DPP1	BDT	DPP2	PDI	Other
CT	1.163	0.001	0.32	0.21	0.44	0.00	0.02	0.00	0.00	0.00	1.00	0.00
LE	1.777	2.179	0.29	0.18	0.52	0.00	0.02	0.48	0.22	0.30	0.00	0.02
TSP2: (DPP-BDT(-PDI-)-DPP S1 geometry												
state	Energy (eV)	Oscillator strength	Hole Distribution					Electron distribution				
			DPP1	BDT	DPP2	PDI	Other	DPP1	BDT	DPP2	PDI	Other
CT	0.695	0	0.18	0.22	0.58	0.00	0.02	0.00	0.00	0.00	1.00	0.00
LE	1.606	1.969	0.15	0.18	0.66	0.00	0.02	0.54	0.22	0.24	0.00	0.02

Table 3. Calculated energies, oscillator strengths, and electron and hole distributions (in fractions of an electron) for the lowest energy state (CT) and the first excitonic state of TSP3 and TSP2 in their ground state geometry (S0) and the relaxed geometry of its first excited state (S1). The electron and hole distribution analysis is done using the TheoDORE package. The rows are colored from red to yellow to light blue to dark blue in a scale from 0 (no charge in fragment) to 1 (all charge in fragment).

s	Reorganisation energy (eV)
TSP2: (DPP-BDT(-PDI-)-DPP	0.43
TSP3: (BDT-DPP(-PDI-)-BDT	0.51

Table 4. Reorganization energy calculated using functional/basis set B3LYP/6-31g* for transition from relaxed first CT state to ground for 1.5-mers of TSP2 and TSP3. Calculated using the Dushin program developed by Reimers et al. (J. Chem. Phys. 2001, 115 (20), 9103–9109. https://doi.org/10.1063/1.1412875) We note that TDDFT can be highly inaccurate to capture the change in the geometry with the change in the excitation state for large molecular systems. Hence these results can have a large error (>0.05 eV) which is hard to estimate without a better method to calculate this property.

10. Kinetic modelling

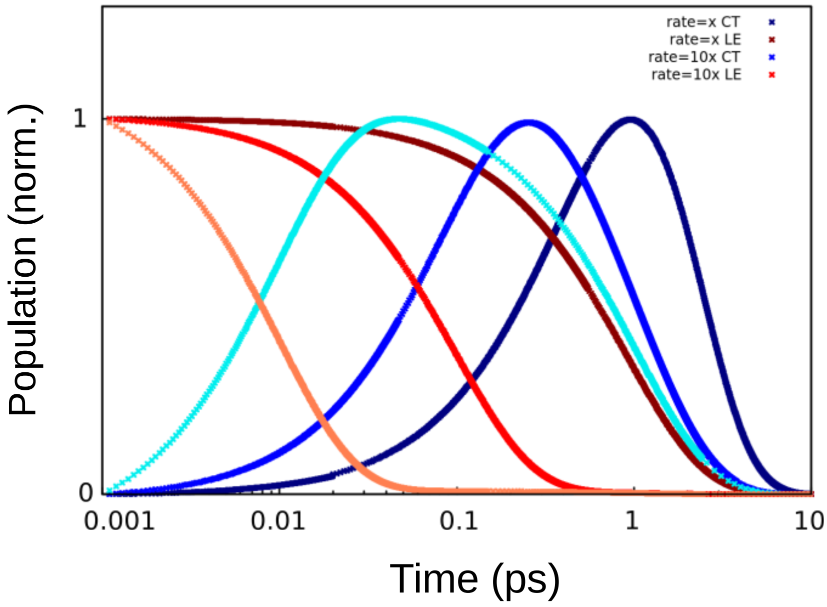

Figure 23. Kinetic model of a three-state system (ground state (GS), local exciton (LE), charge transfer (CT)) where the rate of electron transfer from LE to CT is varied, i.e. through changing the LE to CT electronic coupling. An earlier appearance of CT states (blue) can be observed as well as a faster LE population decrease (red) when the electronic coupling is increased by 100-fold

11. Ground state dipole moments

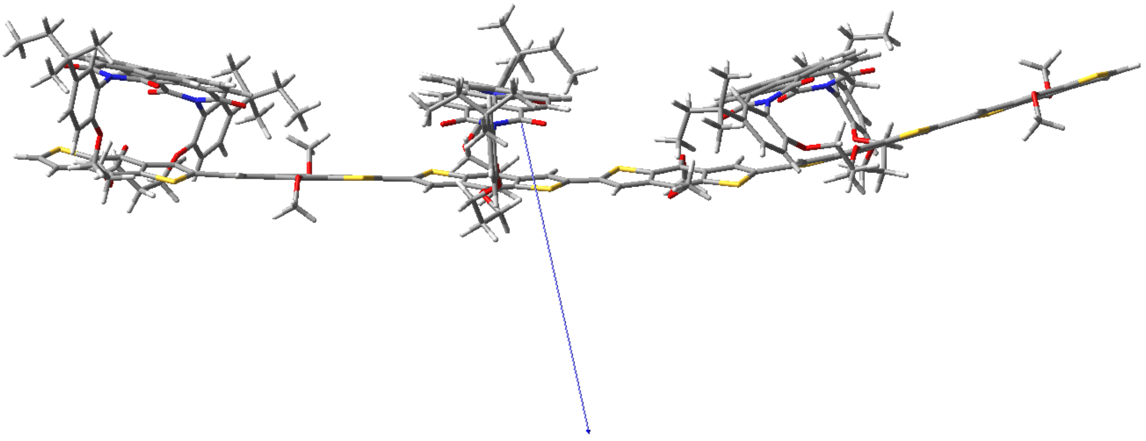

Figure 24. TSP1 ground state geometry with dipole moment, g=6.8391 (blue arrow) from the centre of mass.

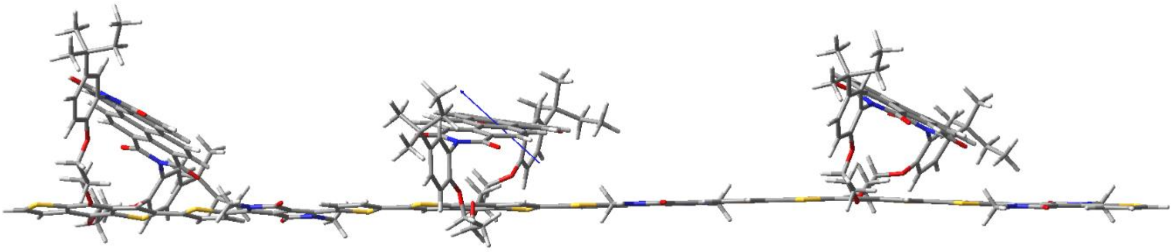

Figure 25. TSP2 ground state geometry with dipole moment, g=4.4000 (blue arrow) from the centre of mass.

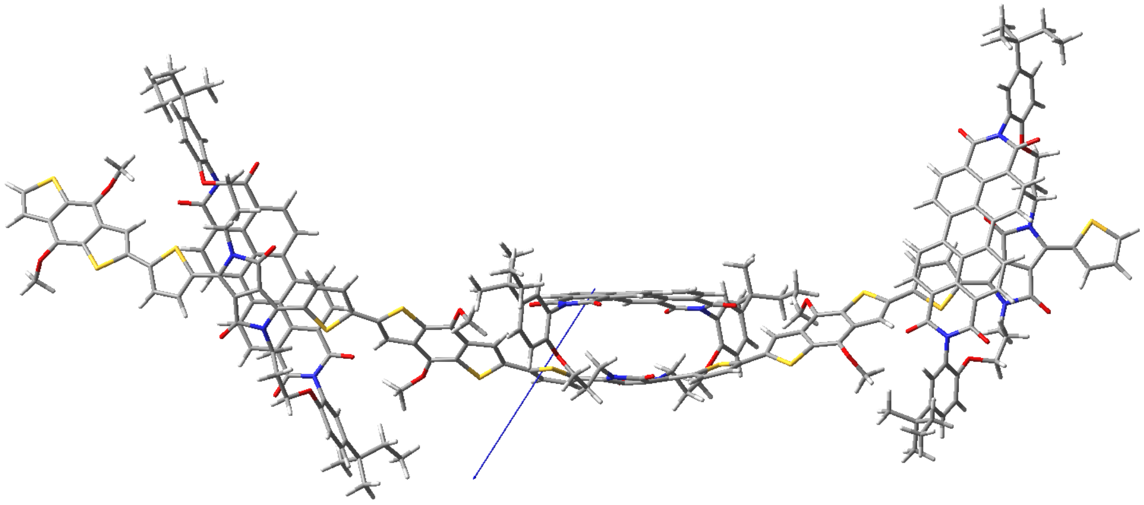

Figure 26. TSP ground state geometry with dipole moment, g=3.9744 Debye (blue arrow) from the centre of mass.

12. Frontier molecular orbital energy levels

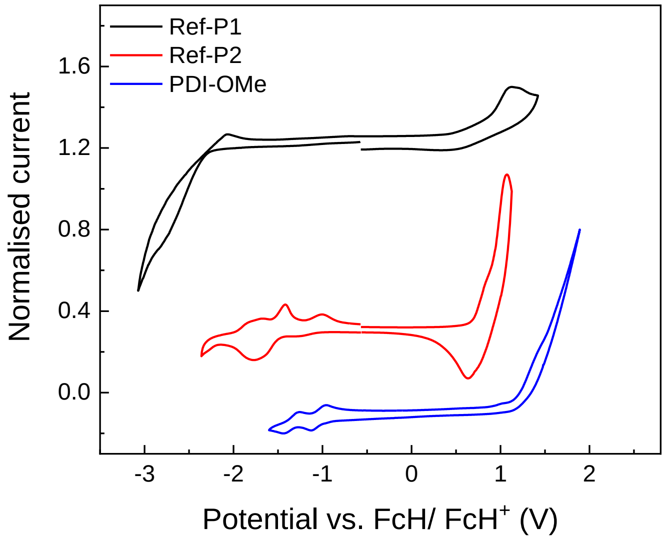

Figure 27. Cyclic voltammograms of BDT-BDT and BDT-DPP films vs. PDI-OMe in solution

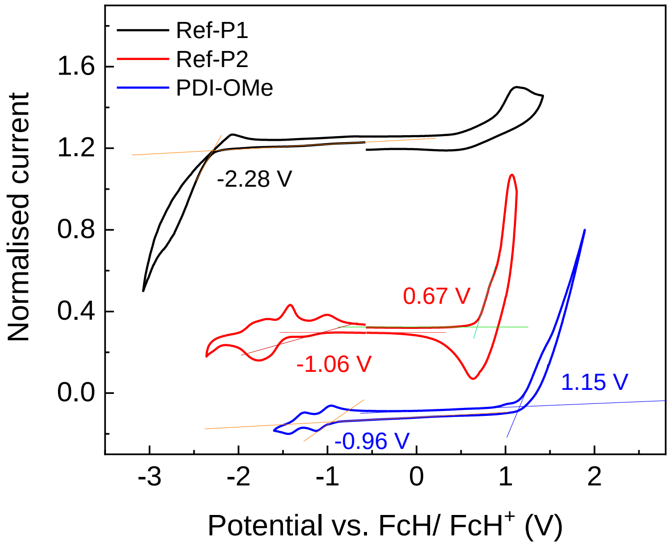

Figure 28. Cyclic voltammograms of BDT-BDT and BDT-DPP films vs. PDI-OMe with oxidation and reduction onsets.

	Ox_{onset} (V)	Red_{onset} (V)	HOMO (eV)[a]	LUMO (eV)[b]
**Ref-P1**	-[c]	−2.28	-5.16[c]	−2.82
**Ref-P2**	0.67	−1.06	-5.77	−4.04
**PDI-OMe**	1.15	−0.96	-6.25	−4.14

[a]HOMO levels calculated from CV potentials by HOMO = −5.1 + (−Ox_{onset}) using ferrocene as the standard; [b]LUMO levels calculated from CV potentials by LUMO = −5.1 + (−Red_{onset}), using ferrocene as the standard; [c]A sensible oxidation onset for Ref-P1 could not be observed based on its optical gap (*ca.* 2.25 eV), therefore the HOMO of Ref-P1 is estimated based on that previously reported for the *n*-$C_{12}H_{25}$ analogue in the literature (Hou, J. *et al.* Bandgap and Molecular Energy Level Control of Conjugated Polymer Photovoltaic Materials Based on Benzo [1 , 2-b : 4 , 5-b ′] dithiophene. *Macromolecules* **41,** 6012–6018 (2008).)

Table 5. Electrochemical data

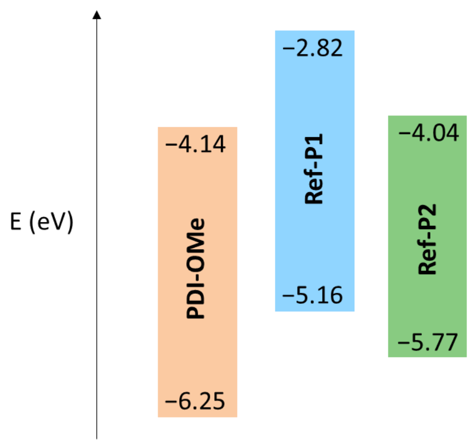

Figure 29. Energy level diagram of Ref polymers vs PDI acceptor

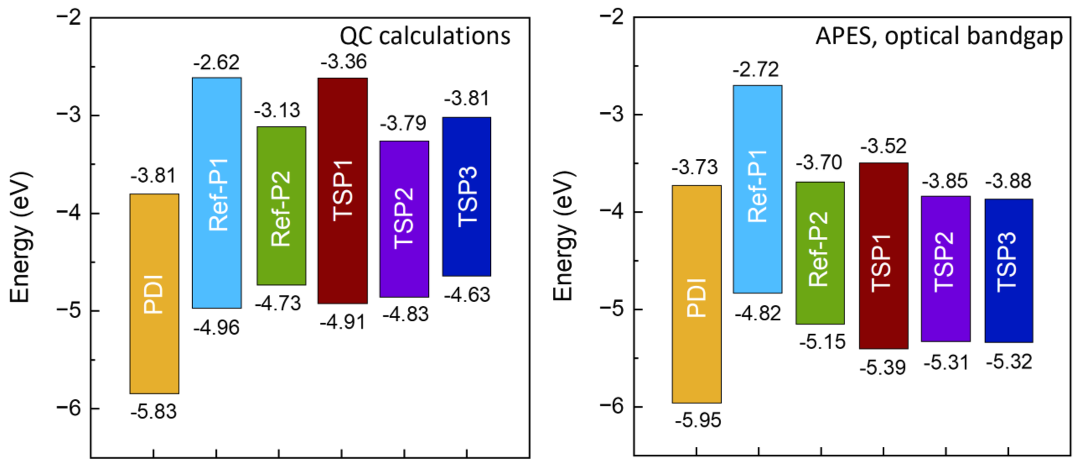

Figure 30. Energy level diagram of (*left*) quantum chemical calculations of PDI, Ref-P1, Ref-P2, TSP1, TSP2 and TSP3 trimers. The LUMOs are obtained by adding the first bright (i.e. excitonic) excited state energy to the HOMO energy. (*right*) Measured ionization potentials (approximated as HOMOs) of PDI, Ref-P1, Ref-P2, TSP1, TSP2 and TSP3 films from air photoemission spectroscopy. The LUMOs are calculated from HOMO energy plus measured optical bandgap (calculated from the cross-over between absorption and emission) of the films.
